# Supplementary material for: The Mineral Oil Hydrocarbon Paradox in Olive Pomace Oils
Source: Foods. 2023 Jan 17;12(3):434. doi: 10.3390/foods12030434 (PMC9914016; doi:10.3390/foods12030434)
Supplement: Supplementary file 1 [file foods-12-00434-s001.zip › Figure S2.pdf]

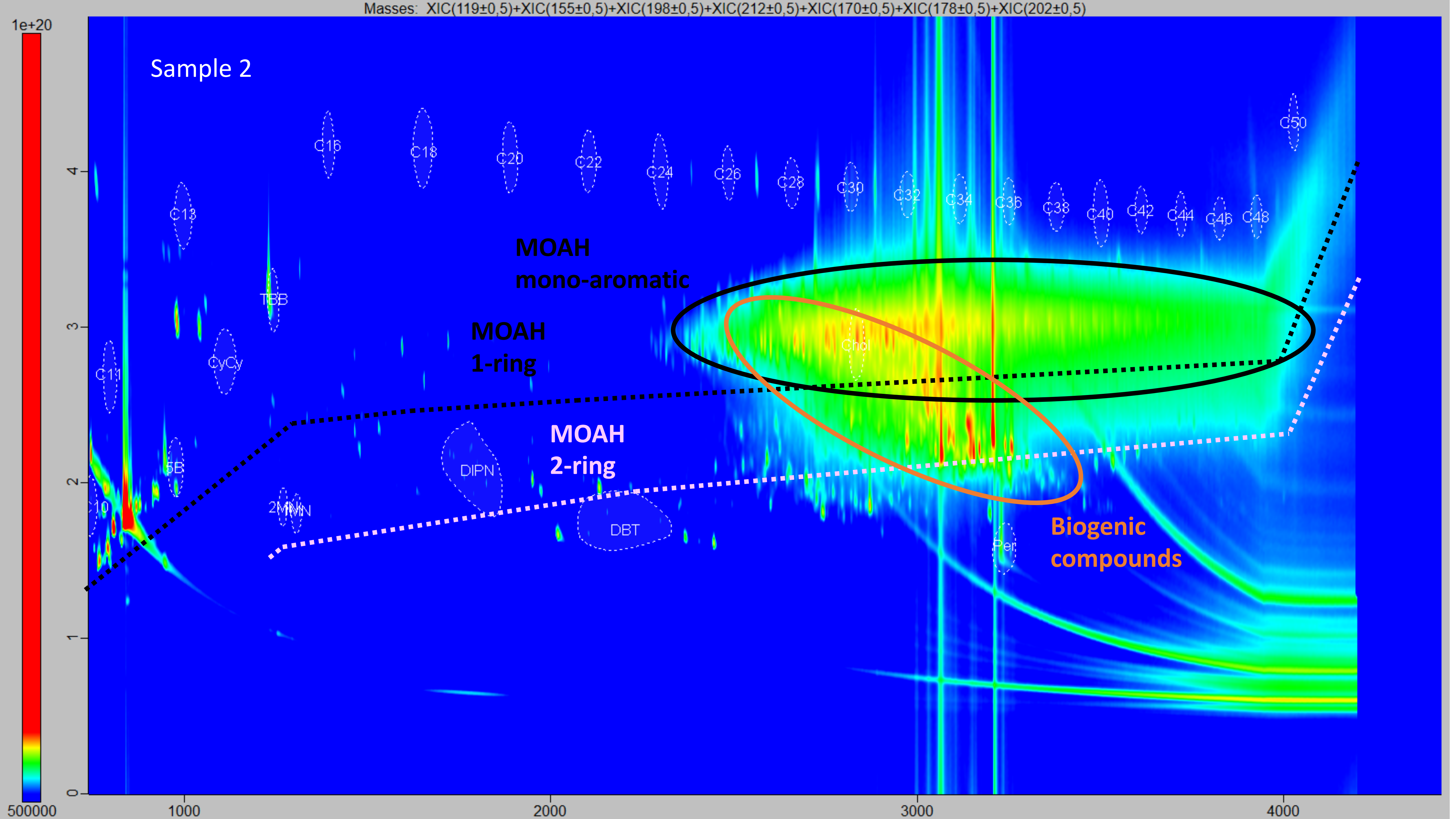

Masses: XIC(119±0,5)+XIC(155±0,5)+XIC(198±0,5)+XIC(212±0,5)+XIC(170±0,5)+XIC(178±0,5)+XIC(202±0,5)
1e+20
500000
Sample 2
C16
C18
C20
C22
C24
C26
C28
C30
C32
C34
C36
C38
C40
C42
C44
C46
C48
C50
C13
C11
C10
TBB
CyCy
5B
2MN
1MN
DIPN
DBT
Chol
Per
MOAH
mono-aromatic
MOAH
1-ring
MOAH
2-ring
Biogenic
compounds
0
1
2
3
4
1000
2000
3000
4000

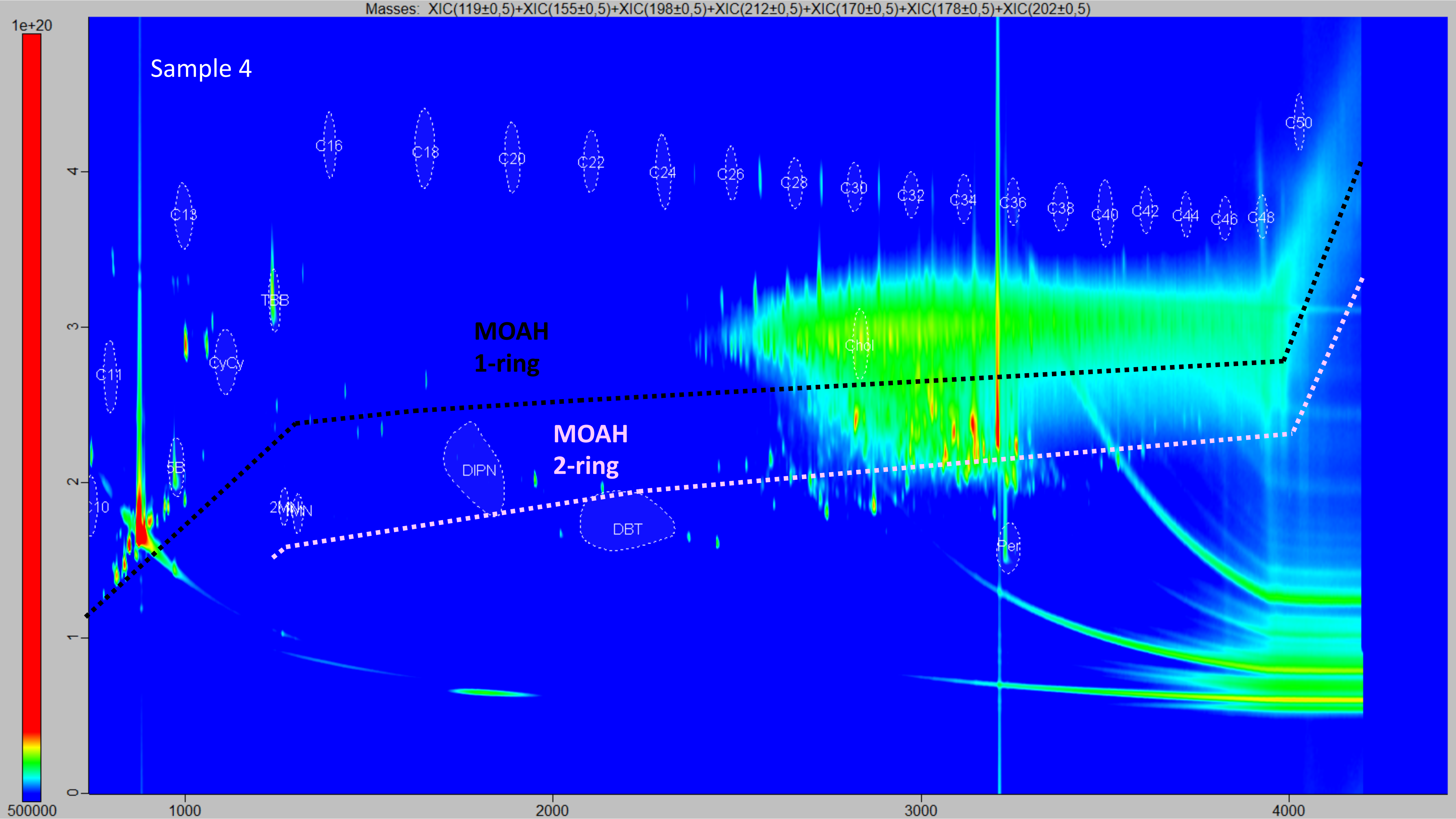

Masses: XIC(119±0,5)+XIC(155±0,5)+XIC(198±0,5)+XIC(212±0,5)+XIC(170±0,5)+XIC(178±0,5)+XIC(202±0,5)
1e+20
500000
Sample 4
C16
C18
C20
C22
C24
C26
C28
C30
C32
C34
C36
C38
C40
C42
C44
C46
C48
C50
C13
C11
C10
TBB
CyCy
5B
DIPN
DBT
Chol
Per
MOAH
1-ring
MOAH
2-ring
0
1
2
3
4
1000
2000
3000
4000

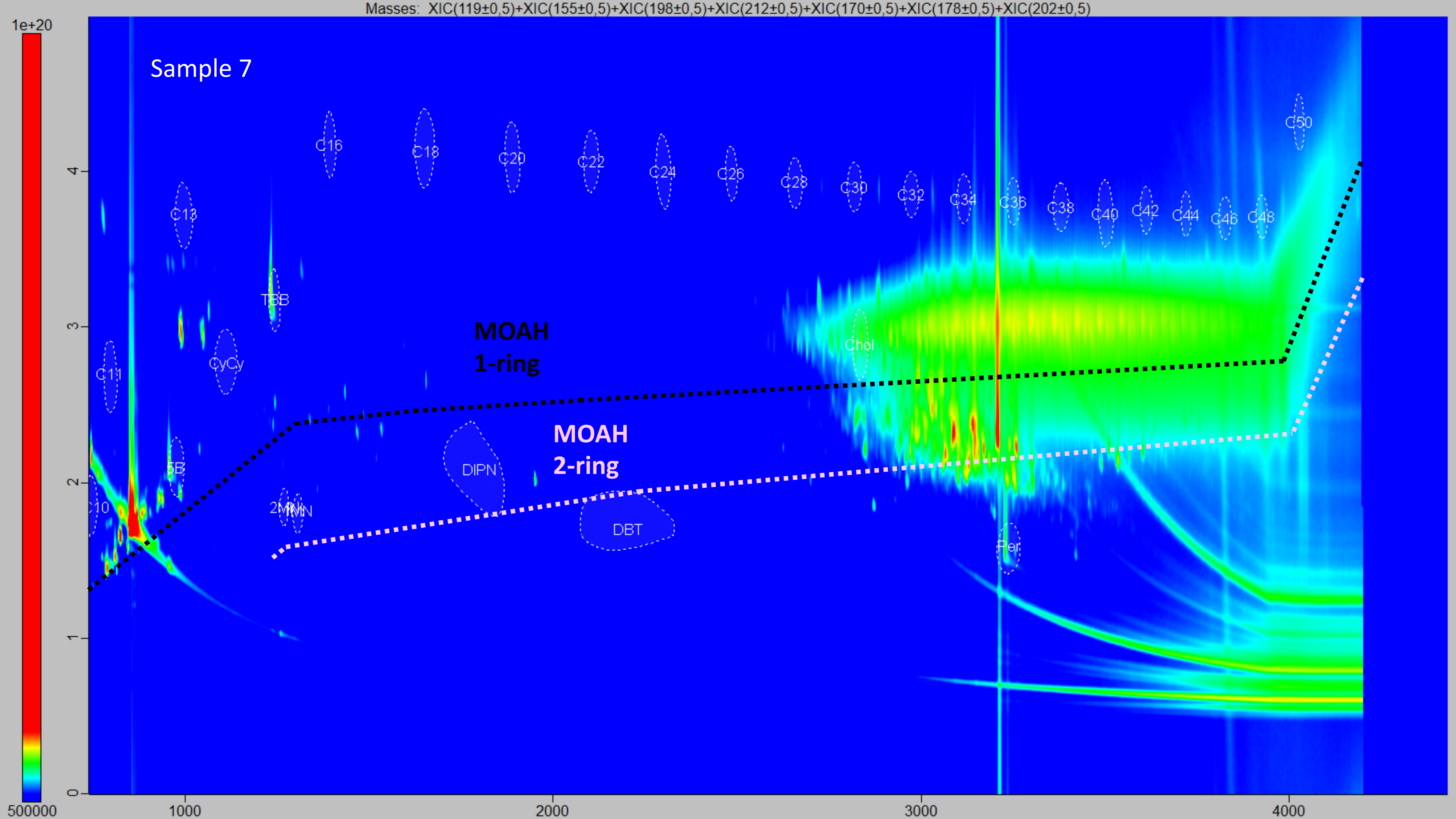

Masses: XIC(119±0,5)+XIC(155±0,5)+XIC(198±0,5)+XIC(212±0,5)+XIC(170±0,5)+XIC(178±0,5)+XIC(202±0,5)
Sample 7
MOAH 1-ring
MOAH 2-ring
C10
C11
C13
C16
C18
C20
C22
C24
C26
C28
C30
C32
C34
C36
C38
C40
C42
C44
C46
C48
C50
TBB
CyCy
5B
DIPN
DBT
Chol
Per
1e+20
500000
1000
2000
3000
4000

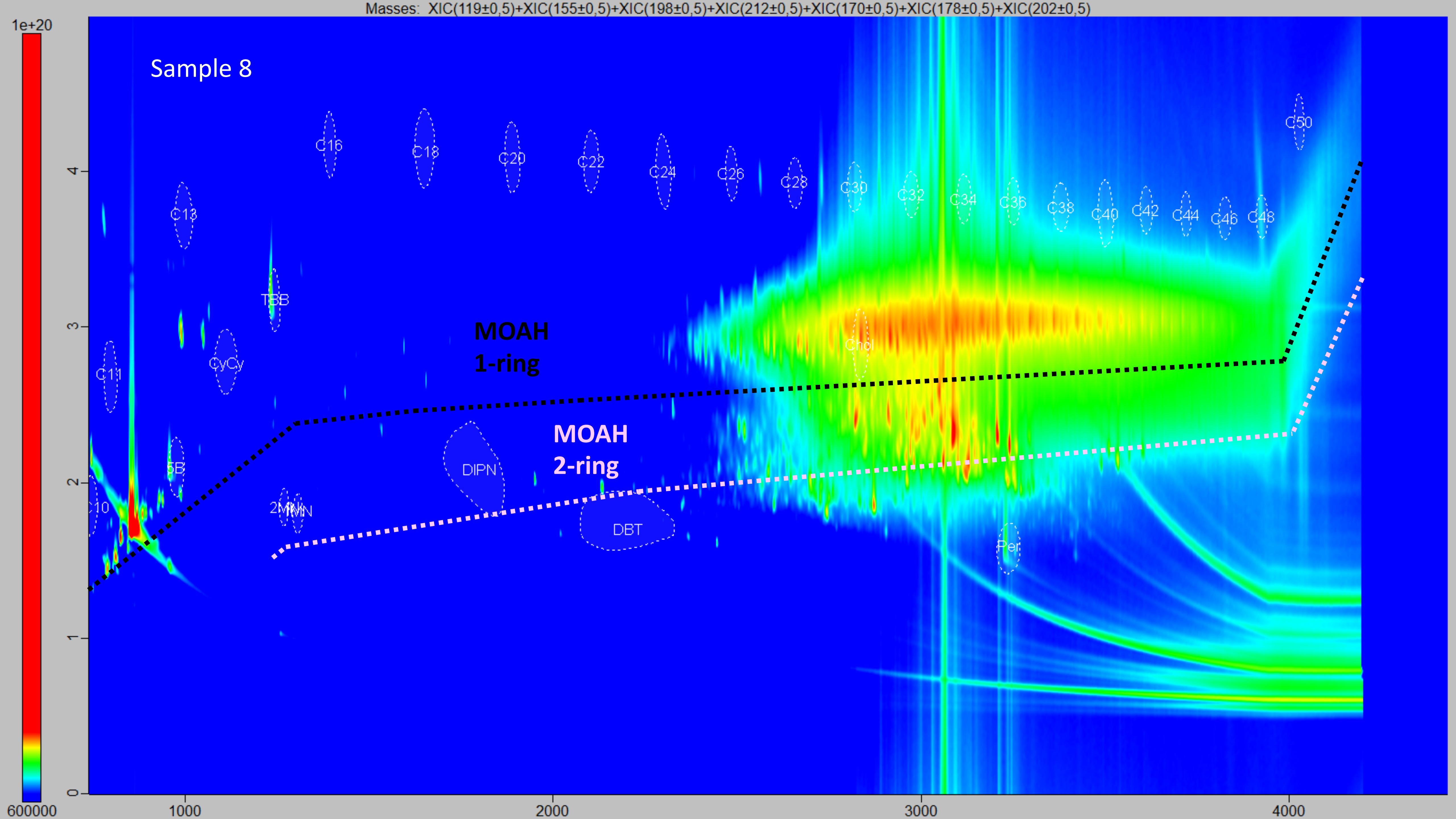
Masses: XIC(119±0,5)+XIC(155±0,5)+XIC(198±0,5)+XIC(212±0,5)+XIC(170±0,5)+XIC(178±0,5)+XIC(202±0,5)
1e+20
600000
Sample 8
MOAH
1-ring
MOAH
2-ring
C10
C11
C13
C16
C18
C20
C22
C24
C26
C28
C30
C32
C34
C36
C38
C40
C42
C44
C46
C48
C50
TBB
CyCy
5B
DIPN
DBT
Chol
Per
0
1
2
3
4
1000
2000
3000
4000

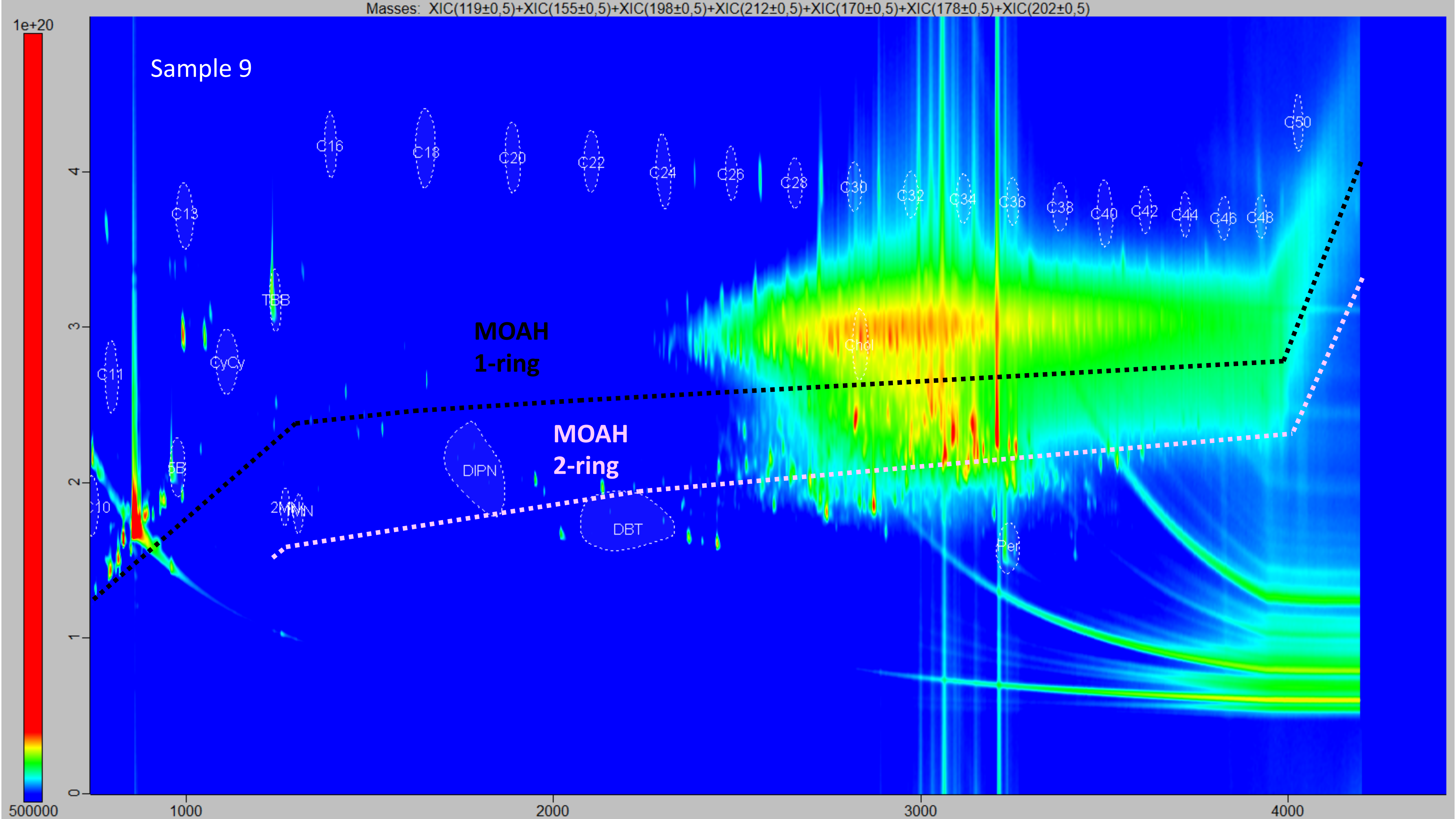

Masses: XIC(119±0,5)+XIC(155±0,5)+XIC(198±0,5)+XIC(212±0,5)+XIC(170±0,5)+XIC(178±0,5)+XIC(202±0,5)
1e+20
500000
Sample 9
MOAH
1-ring
MOAH
2-ring
C10
C11
C13
C16
C18
C20
C22
C24
C26
C28
C30
C32
C34
C36
C38
C40
C42
C44
C46
C48
C50
TBB
CyCy
5B
2MN
1MN
DIPN
DBT
Chol
Per
0
1
2
3
4
1000
2000
3000
4000

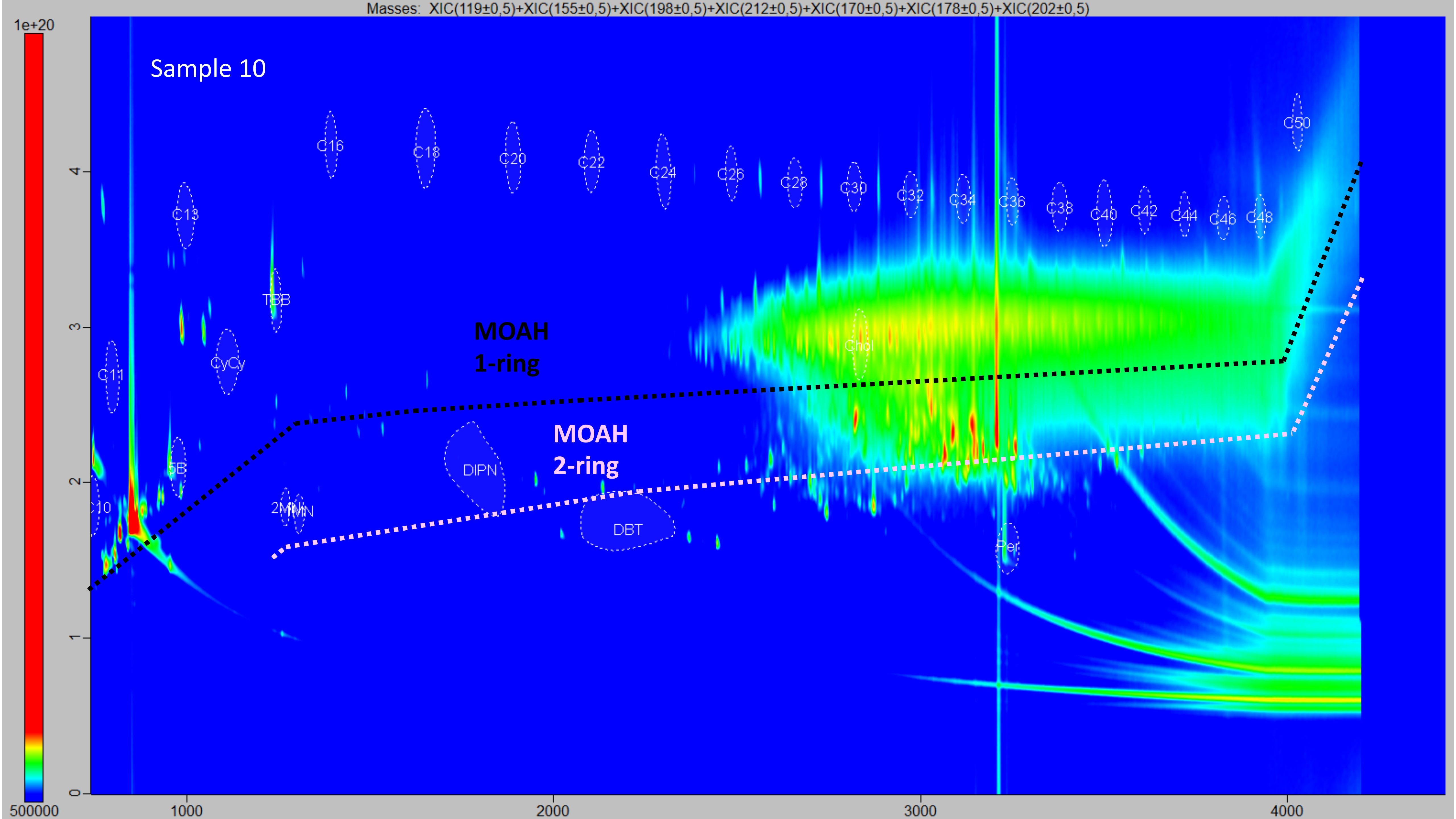

Masses: XIC(119±0,5)+XIC(155±0,5)+XIC(198±0,5)+XIC(212±0,5)+XIC(170±0,5)+XIC(178±0,5)+XIC(202±0,5)
1e+20
500000
Sample 10
C16
C18
C20
C22
C24
C26
C28
C30
C32
C34
C36
C38
C40
C42
C44
C46
C48
C50
C13
C11
TBB
CyCy
5B
2MN
DIPN
DBT
Chol
Per
MOAH 1-ring
MOAH 2-ring
0
1
2
3
4
1000
2000
3000
4000

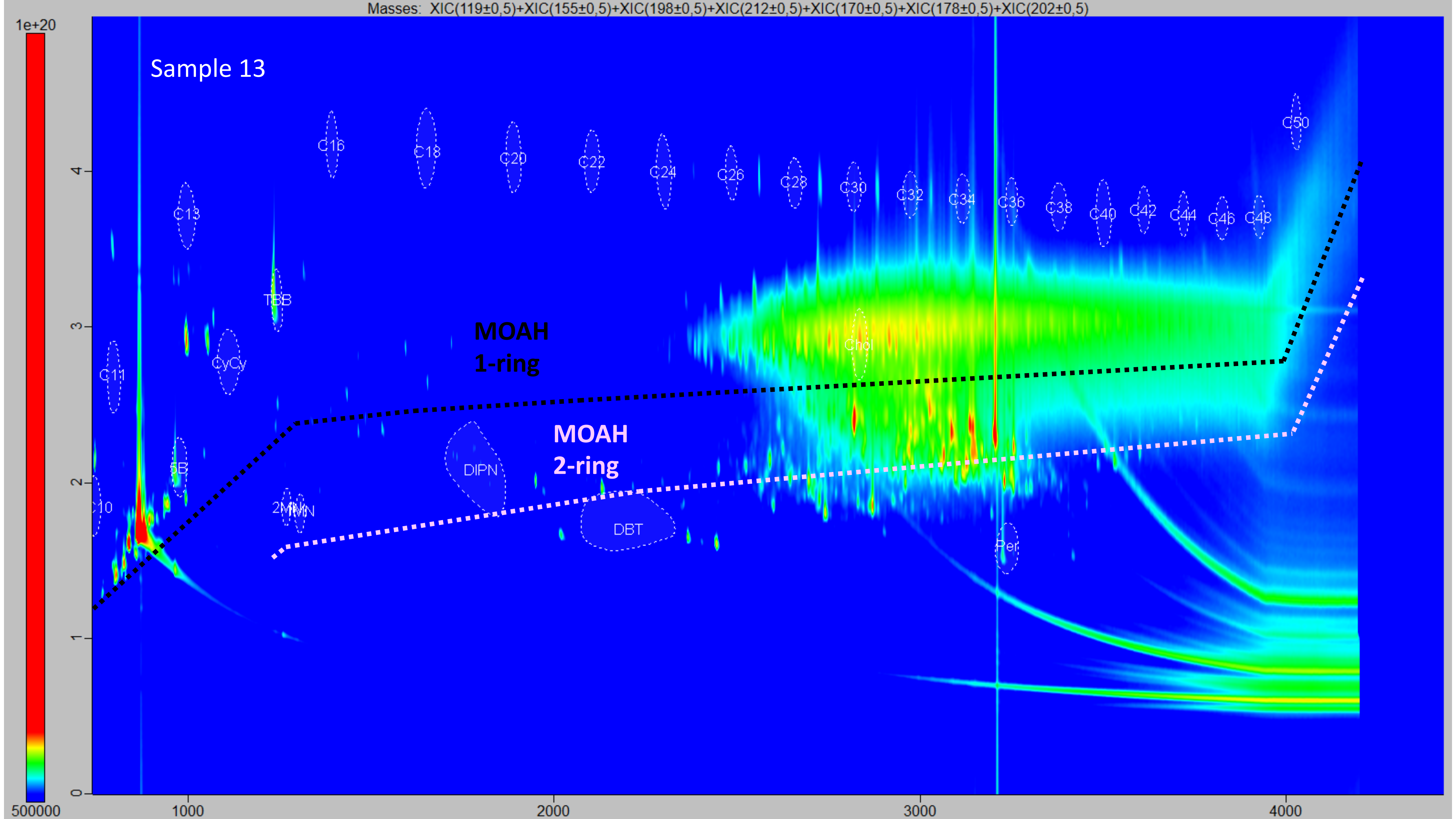
Masses: XIC(119±0,5)+XIC(155±0,5)+XIC(198±0,5)+XIC(212±0,5)+XIC(170±0,5)+XIC(178±0,5)+XIC(202±0,5)
1e+20
500000
Sample 13
MOAH
1-ring
MOAH
2-ring
C11
C13
C16
C18
C20
C22
C24
C26
C28
C30
C32
C34
C36
C38
C40
C42
C44
C46
C48
C50
TBB
CyCy
5B
DIPN
DBT
Chol
Per
0
1
2
3
4
1000
2000
3000
4000

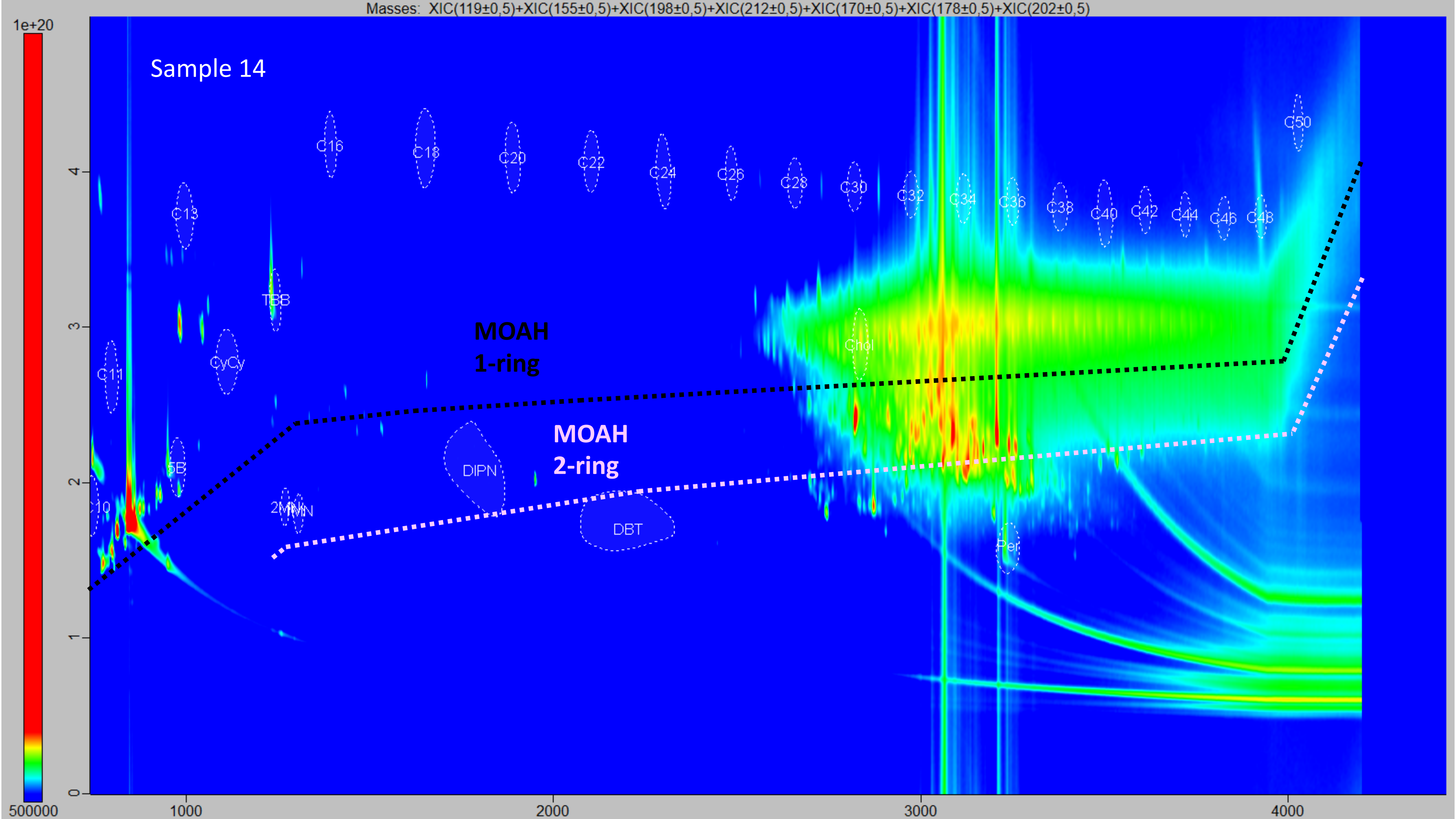
Masses: XIC(119±0,5)+XIC(155±0,5)+XIC(198±0,5)+XIC(212±0,5)+XIC(170±0,5)+XIC(178±0,5)+XIC(202±0,5)
1e+20
500000
Sample 14
MOAH
1-ring
MOAH
2-ring
C11
C13
C16
C18
C20
C22
C24
C26
C28
C30
C32
C34
C36
C38
C40
C42
C44
C46
C48
C50
TBB
CyCy
5B
DIPN
DBT
Chol
Per
0
1
2
3
4
1000
2000
3000
4000

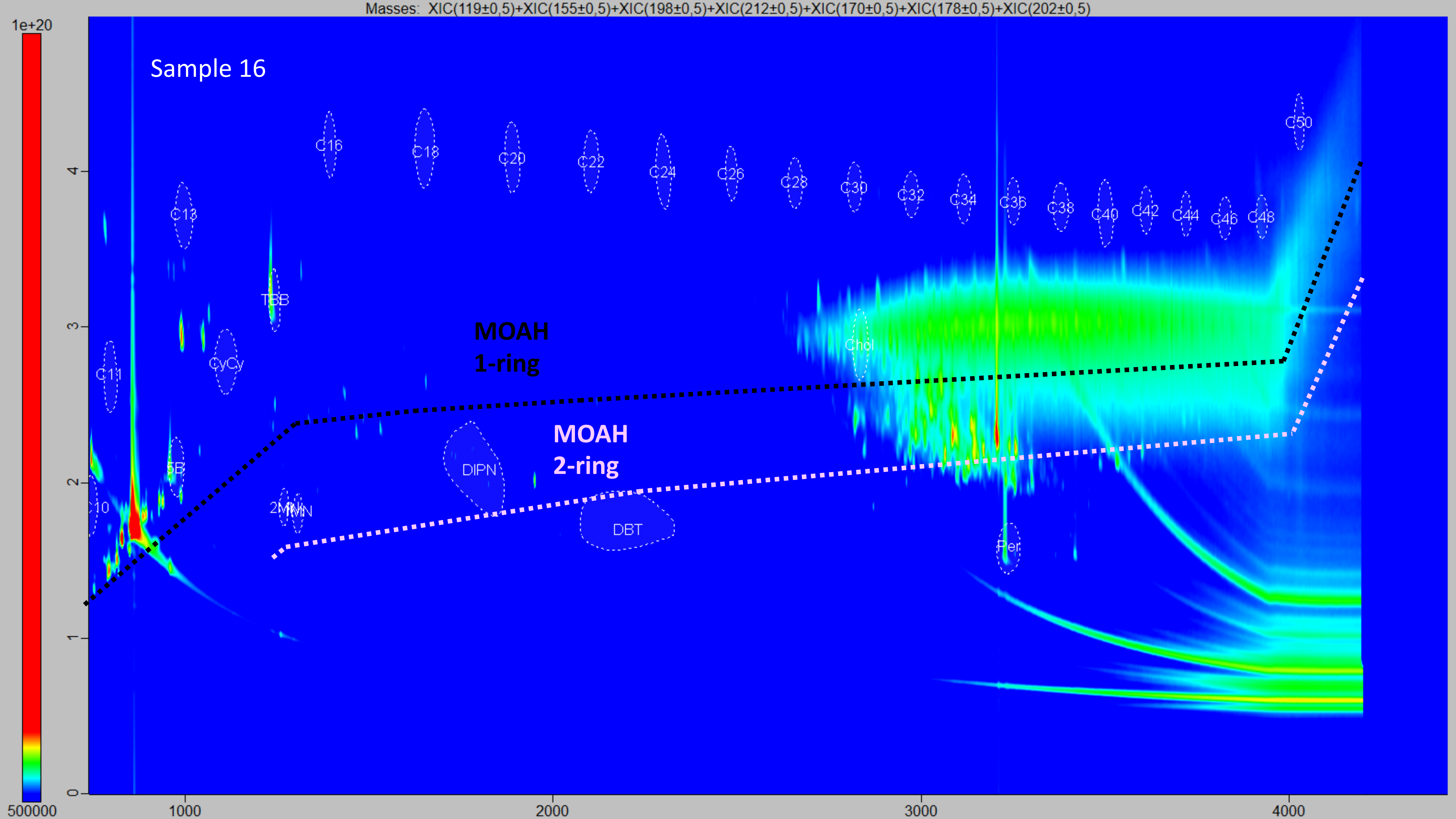
Masses: XIC(119±0,5)+XIC(155±0,5)+XIC(198±0,5)+XIC(212±0,5)+XIC(170±0,5)+XIC(178±0,5)+XIC(202±0,5)
1e+20
500000
Sample 16
C16
C18
C20
C22
C24
C26
C28
C30
C32
C34
C36
C38
C40
C42
C44
C46
C48
C50
C13
C11
C10
CyCy
TBB
5B
2MN
DIPN
DBT
Chol
Per
MOAH
1-ring
MOAH
2-ring
0
1
2
3
4
1000
2000
3000
4000

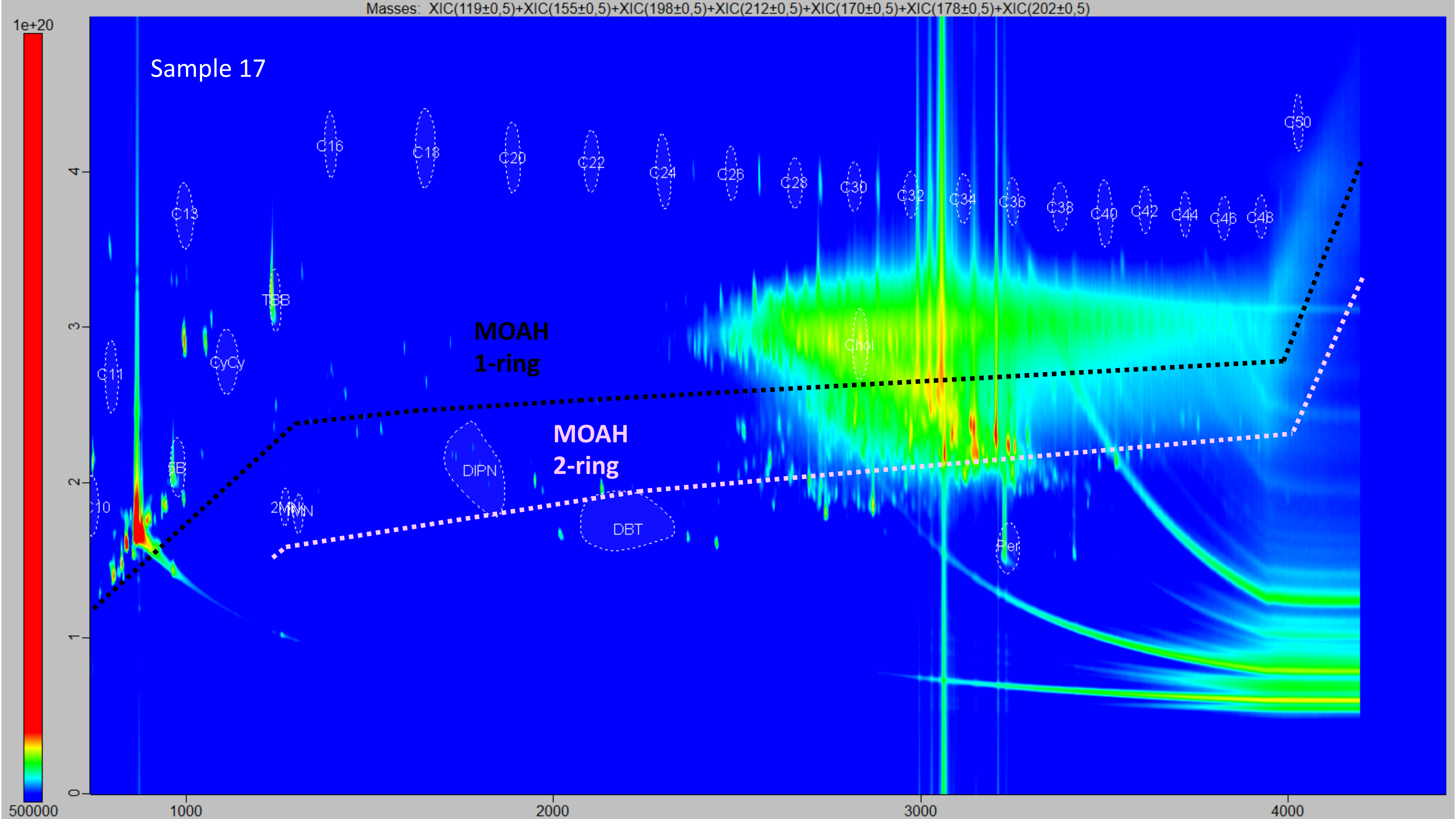

Masses: XIC(119±0,5)+XIC(155±0,5)+XIC(198±0,5)+XIC(212±0,5)+XIC(170±0,5)+XIC(178±0,5)+XIC(202±0,5)
Sample 17
1e+20
500000
MOAH
1-ring
MOAH
2-ring
C11
C13
C16
C18
C20
C22
C24
C26
C28
C30
C32
C34
C36
C38
C40
C42
C44
C46
C48
C50
TBB
CyCy
DIPN
DBT
Chol
Per
0
1
2
3
4
1000
2000
3000
4000

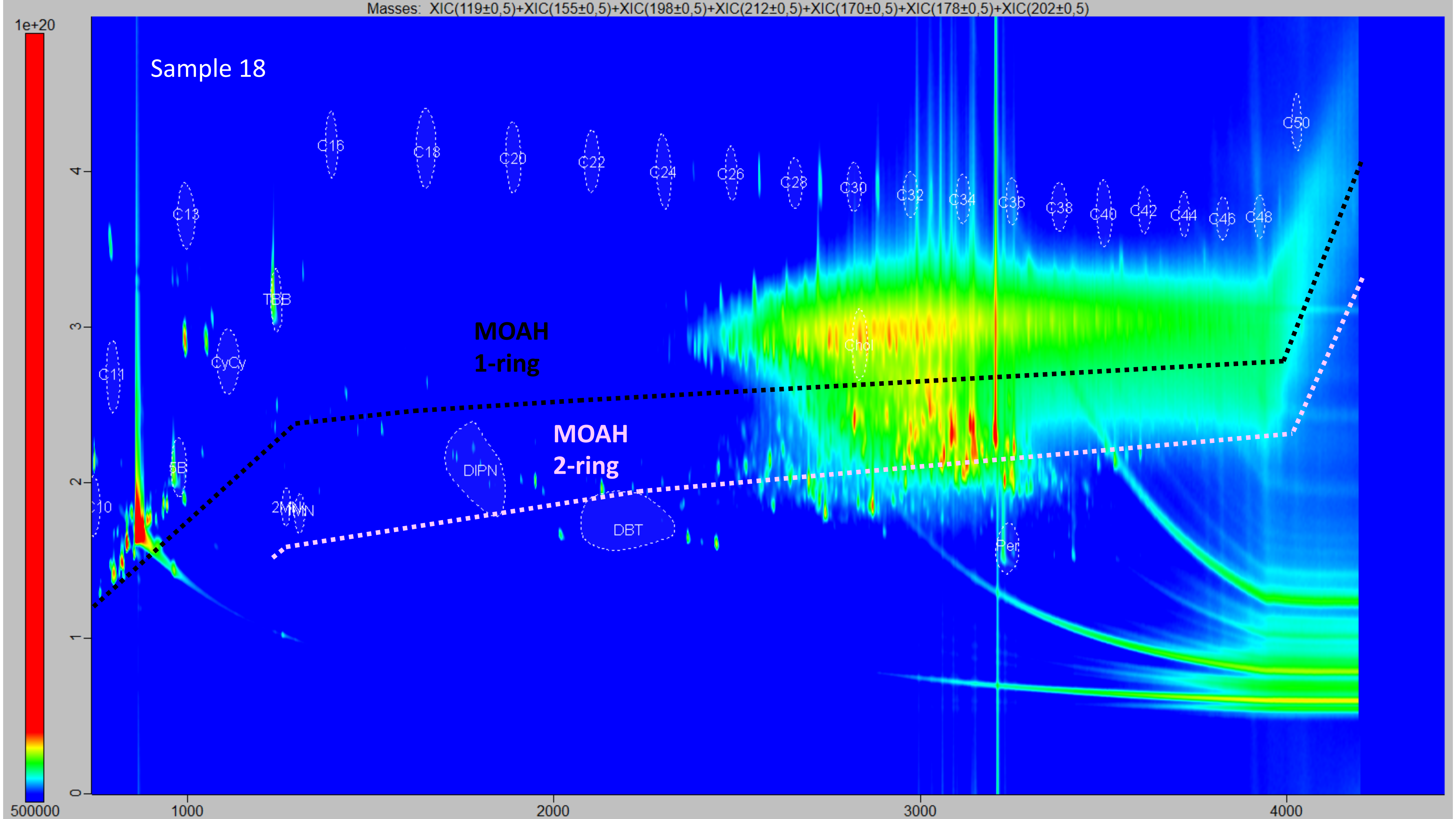
Masses: XIC(119±0,5)+XIC(155±0,5)+XIC(198±0,5)+XIC(212±0,5)+XIC(170±0,5)+XIC(178±0,5)+XIC(202±0,5)
1e+20
500000
Sample 18
MOAH
1-ring
MOAH
2-ring
C10
C11
C13
C16
C18
C20
C22
C24
C26
C28
C30
C32
C34
C36
C38
C40
C42
C44
C46
C48
C50
TBB
CyCy
5B
DIPN
DBT
Chol
Per
0
1
2
3
4
1000
2000
3000
4000

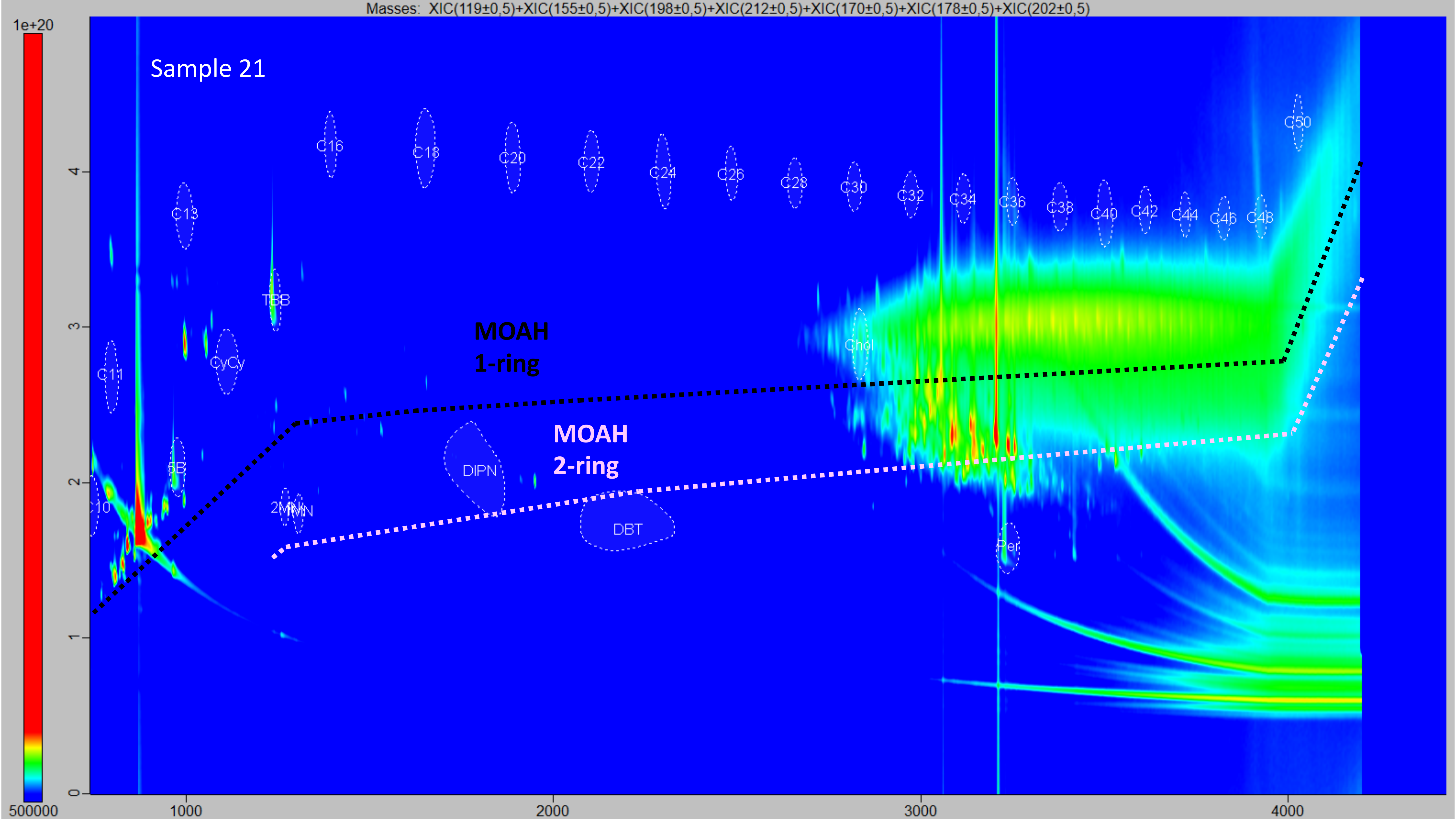

Masses: XIC(119±0,5)+XIC(155±0,5)+XIC(198±0,5)+XIC(212±0,5)+XIC(170±0,5)+XIC(178±0,5)+XIC(202±0,5)
1e+20
500000
Sample 21
C16
C18
C20
C22
C24
C26
C28
C30
C32
C34
C36
C38
C40
C42
C44
C46
C48
C50
C13
C11
C10
TBB
CyCy
5B
2MN
1MN
DIPN
DBT
Chol
Per
MOAH
1-ring
MOAH
2-ring
0
1
2
3
4
1000
2000
3000
4000

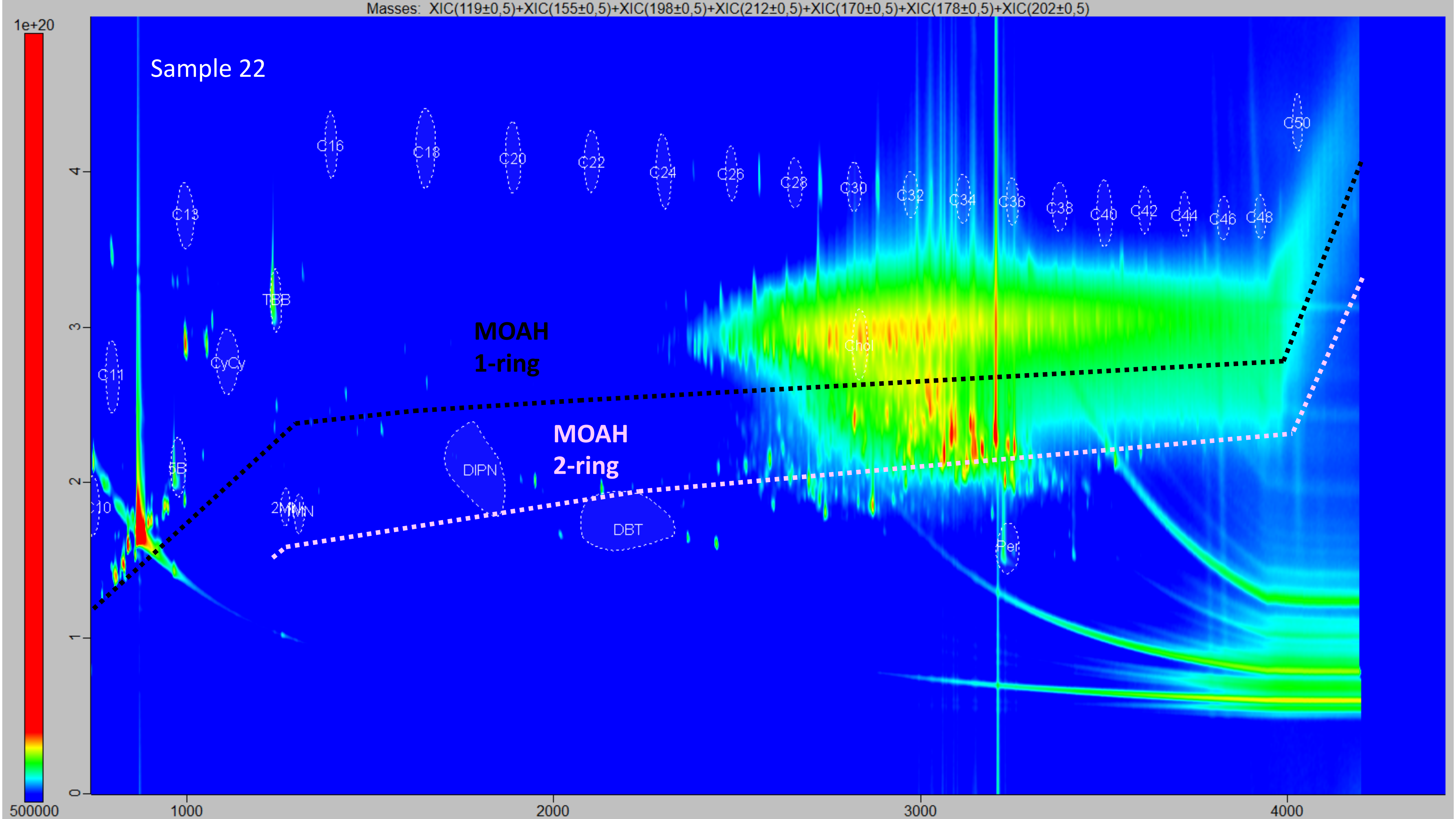

Masses: XIC(119±0,5)+XIC(155±0,5)+XIC(198±0,5)+XIC(212±0,5)+XIC(170±0,5)+XIC(178±0,5)+XIC(202±0,5)
1e+20
500000
Sample 22
C16
C18
C20
C22
C24
C26
C28
C30
C32
C34
C36
C38
C40
C42
C44
C46
C48
C50
C13
C11
C10
TBB
CyCy
5B
2MN
1MN
DIPN
DBT
Chol
Per
MOAH
1-ring
MOAH
2-ring
0
1
2
3
4
1000
2000
3000
4000

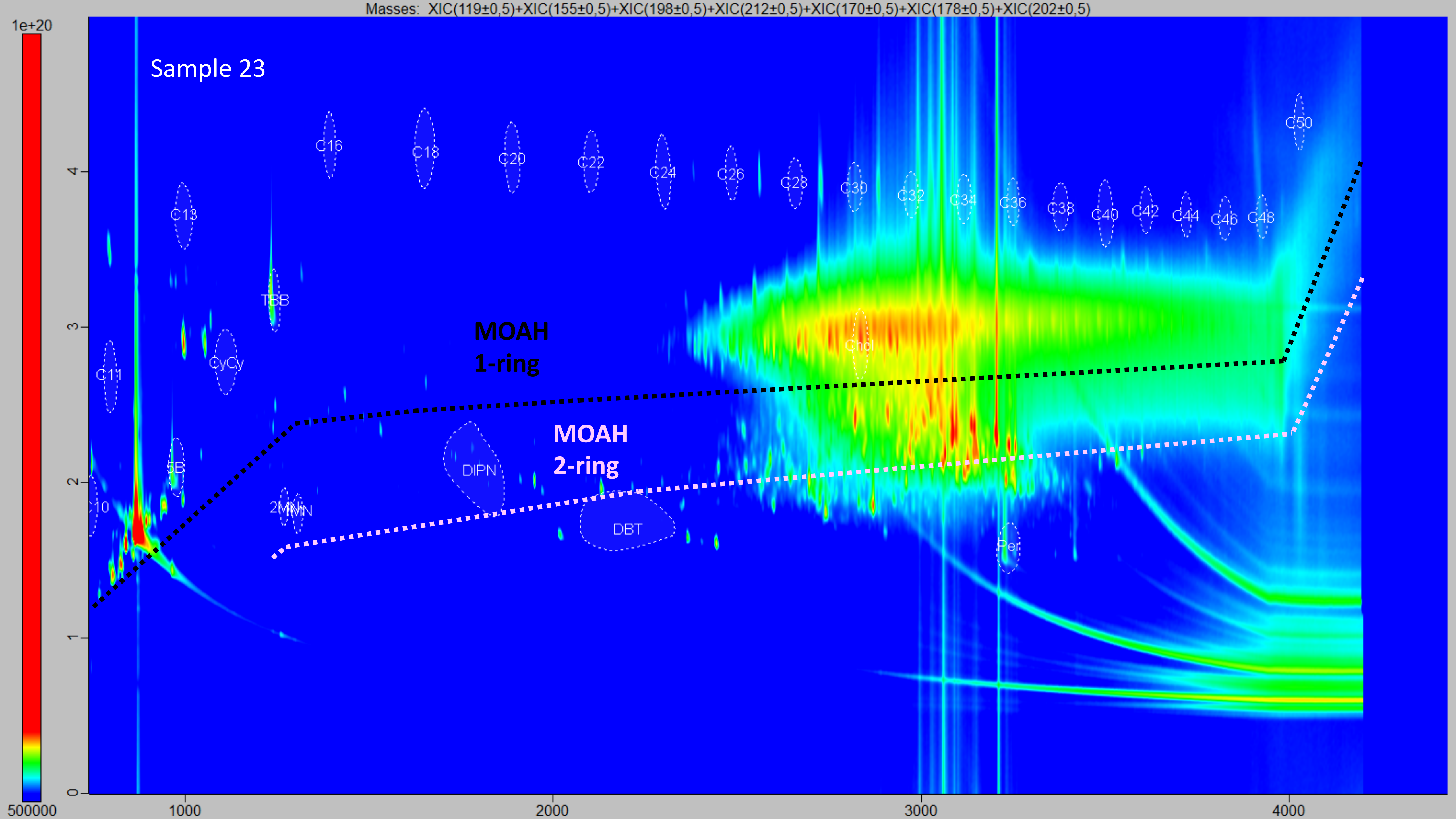
Masses: XIC(119±0,5)+XIC(155±0,5)+XIC(198±0,5)+XIC(212±0,5)+XIC(170±0,5)+XIC(178±0,5)+XIC(202±0,5)
1e+20
500000
Sample 23
C16
C18
C20
C22
C24
C26
C28
C30
C32
C34
C36
C38
C40
C42
C44
C46
C48
C50
C13
C11
C10
TBB
CyCy
5B
2MN
DIPN
DBT
Chol
Per
MOAH
1-ring
MOAH
2-ring
0
1
2
3
4
1000
2000
3000
4000

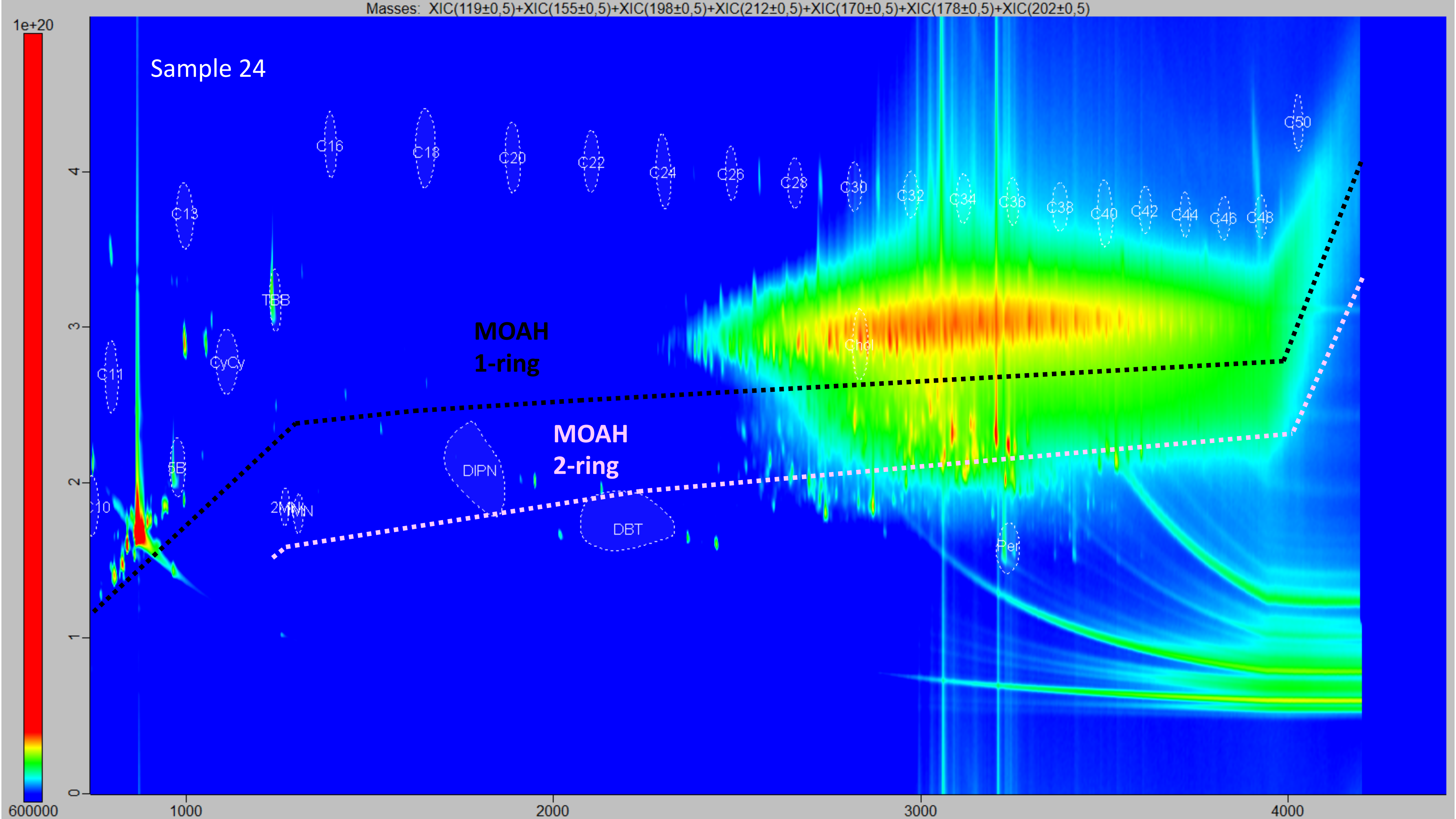

Masses: XIC(119±0,5)+XIC(155±0,5)+XIC(198±0,5)+XIC(212±0,5)+XIC(170±0,5)+XIC(178±0,5)+XIC(202±0,5)
1e+20
600000
Sample 24
MOAH
1-ring
MOAH
2-ring
C10
C11
C13
C16
C18
C20
C22
C24
C26
C28
C30
C32
C34
C36
C38
C40
C42
C44
C46
C48
C50
TBB
CyCy
5B
DIPN
DBT
Chol
Per
0
1
2
3
4
1000
2000
3000
4000

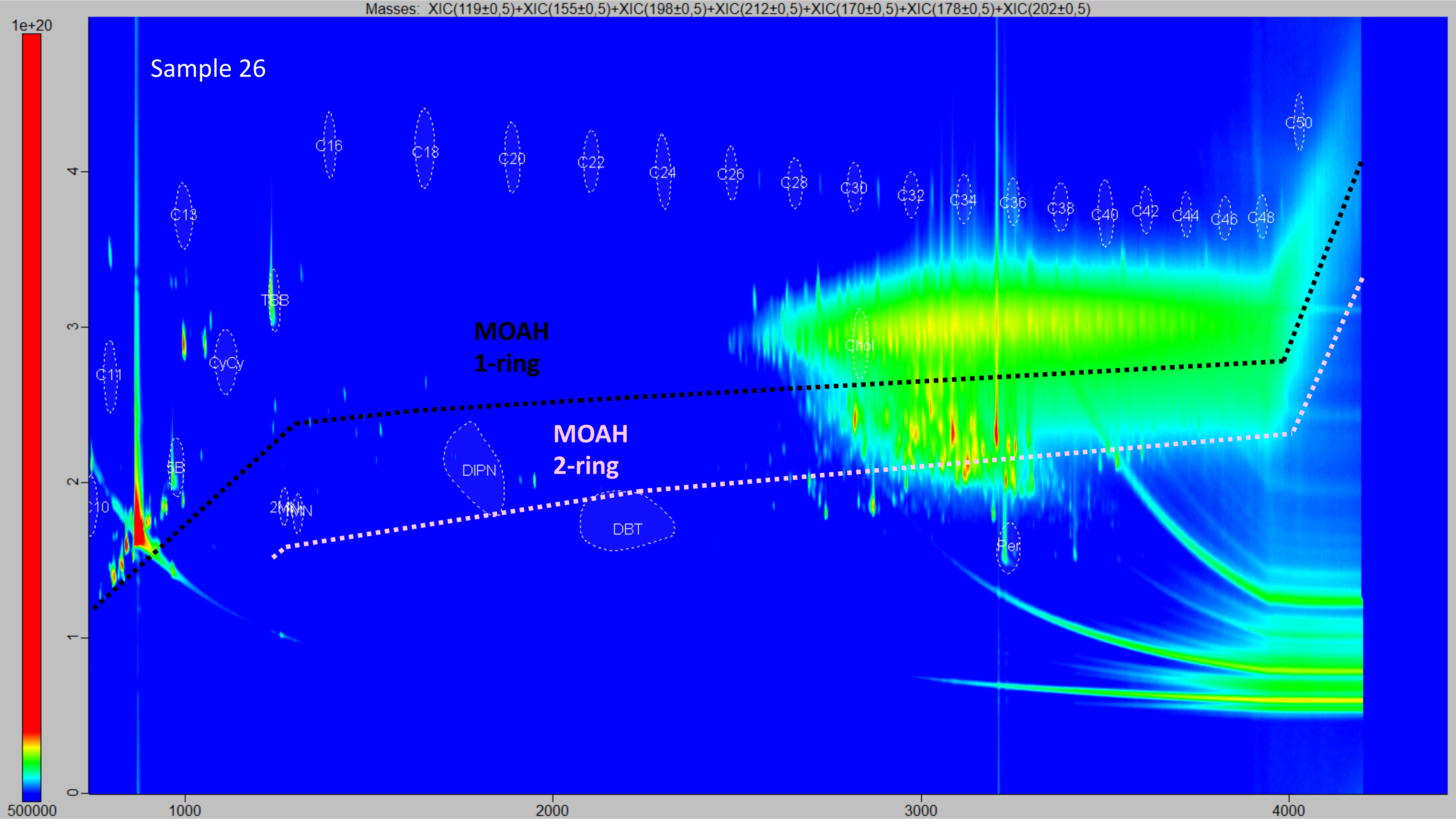
Masses: XIC(119±0,5)+XIC(155±0,5)+XIC(198±0,5)+XIC(212±0,5)+XIC(170±0,5)+XIC(178±0,5)+XIC(202±0,5)
Sample 26
MOAH
1-ring
MOAH
2-ring
C10
C11
C13
C16
C18
C20
C22
C24
C26
C28
C30
C32
C34
C36
C38
C40
C42
C44
C46
C48
C50
TBB
CyCy
5B
2MN
DIPN
DBT
Chol
Per
1e+20
500000
1000
2000
3000
4000
0
1
2
3
4

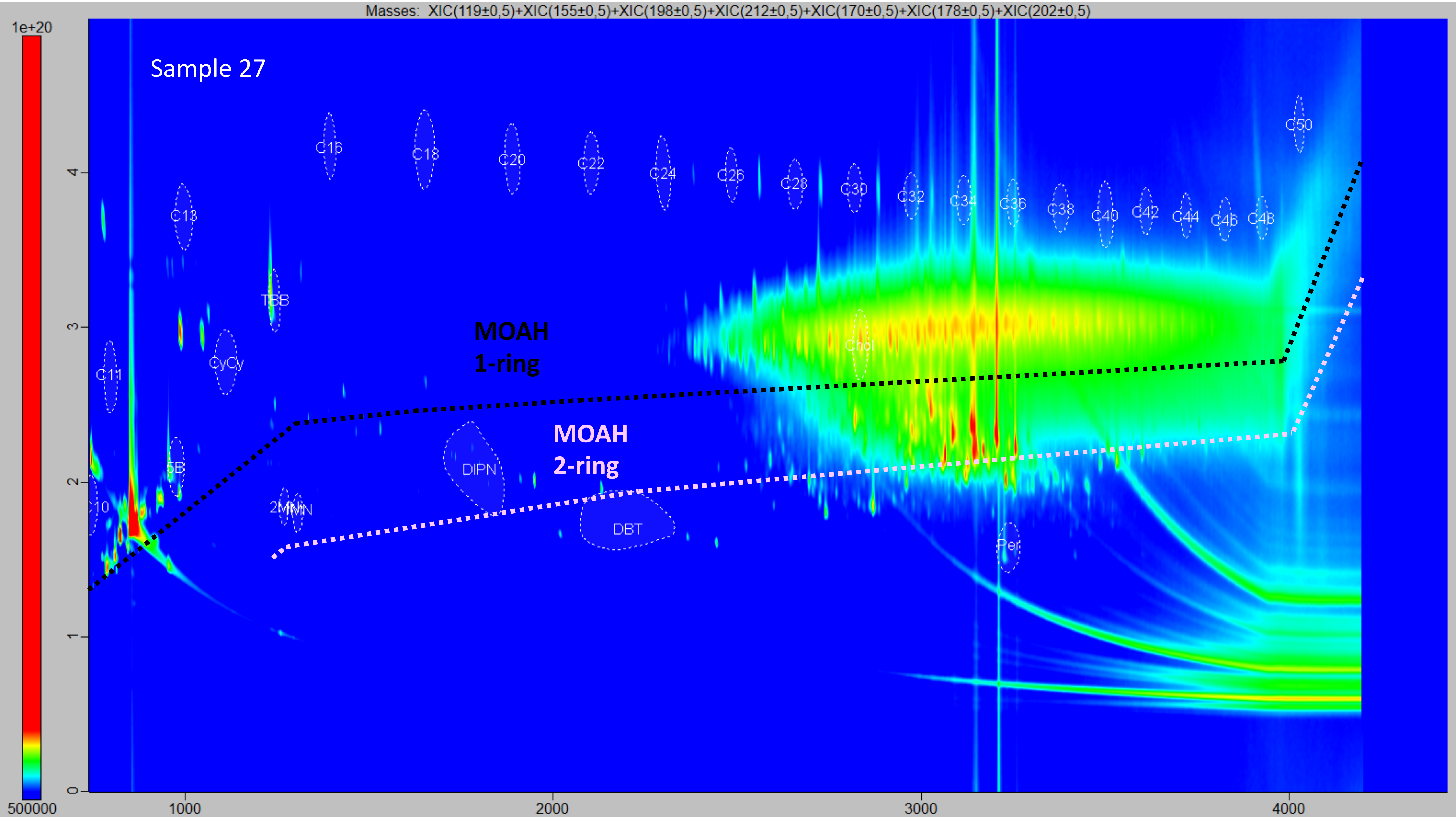
Masses: XIC(119±0,5)+XIC(155±0,5)+XIC(198±0,5)+XIC(212±0,5)+XIC(170±0,5)+XIC(178±0,5)+XIC(202±0,5)
1e+20
500000
Sample 27
MOAH
1-ring
MOAH
2-ring
C10
C11
C13
C16
C18
C20
C22
C24
C26
C28
C30
C32
C34
C36
C38
C40
C42
C44
C46
C48
C50
TBB
CyCy
5B
DIPN
DBT
Chol
Per
0
1
2
3
4
1000
2000
3000
4000

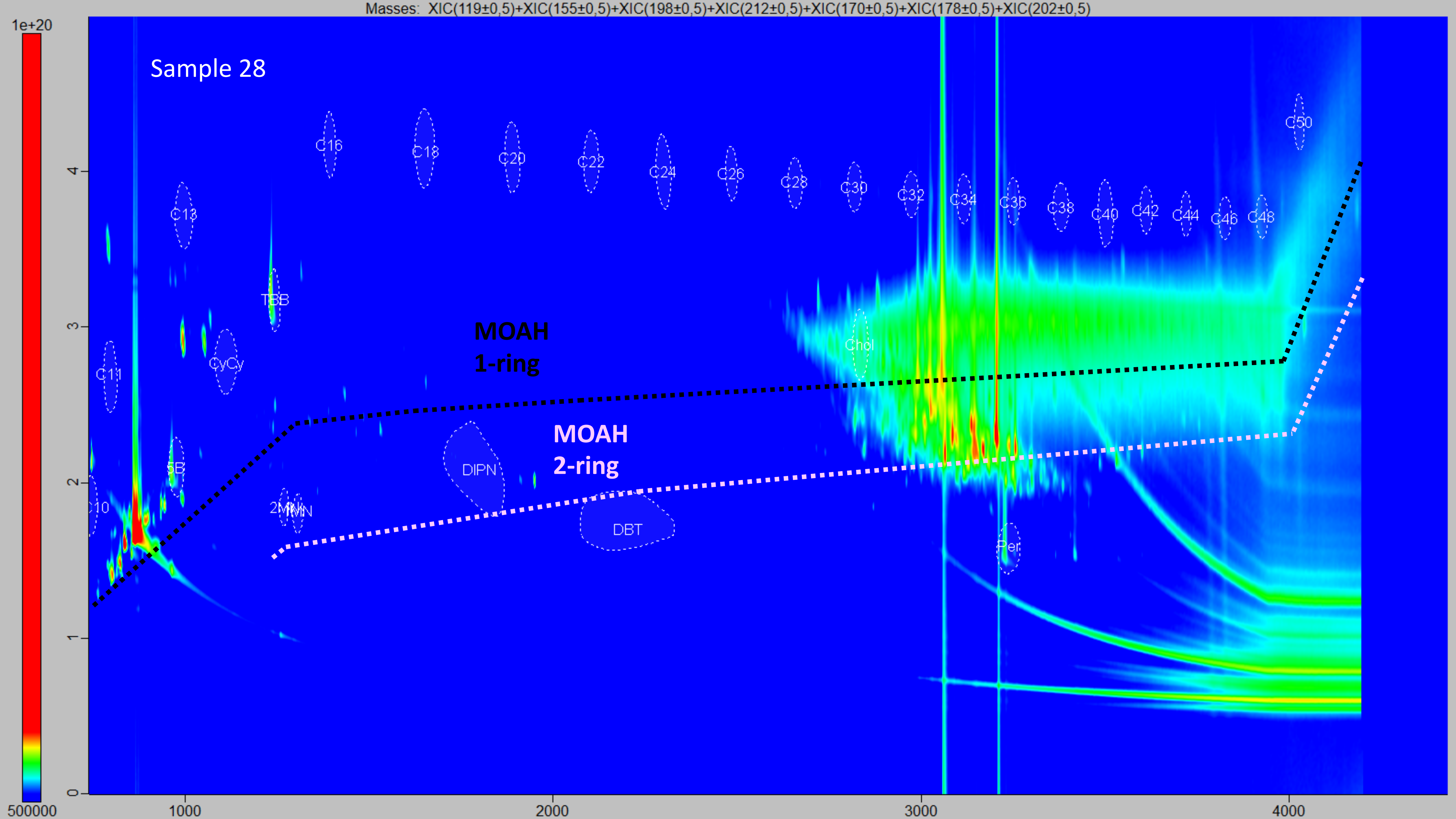
Masses: XIC(119±0,5)+XIC(155±0,5)+XIC(198±0,5)+XIC(212±0,5)+XIC(170±0,5)+XIC(178±0,5)+XIC(202±0,5)
1e+20
500000
Sample 28
MOAH
1-ring
MOAH
2-ring
C10
C11
C13
C16
C18
C20
C22
C24
C26
C28
C30
C32
C34
C36
C38
C40
C42
C44
C46
C48
C50
TBB
CyCy
5B
2MN
1MN
DIPN
DBT
Chol
Per
0
1
2
3
4
1000
2000
3000
4000

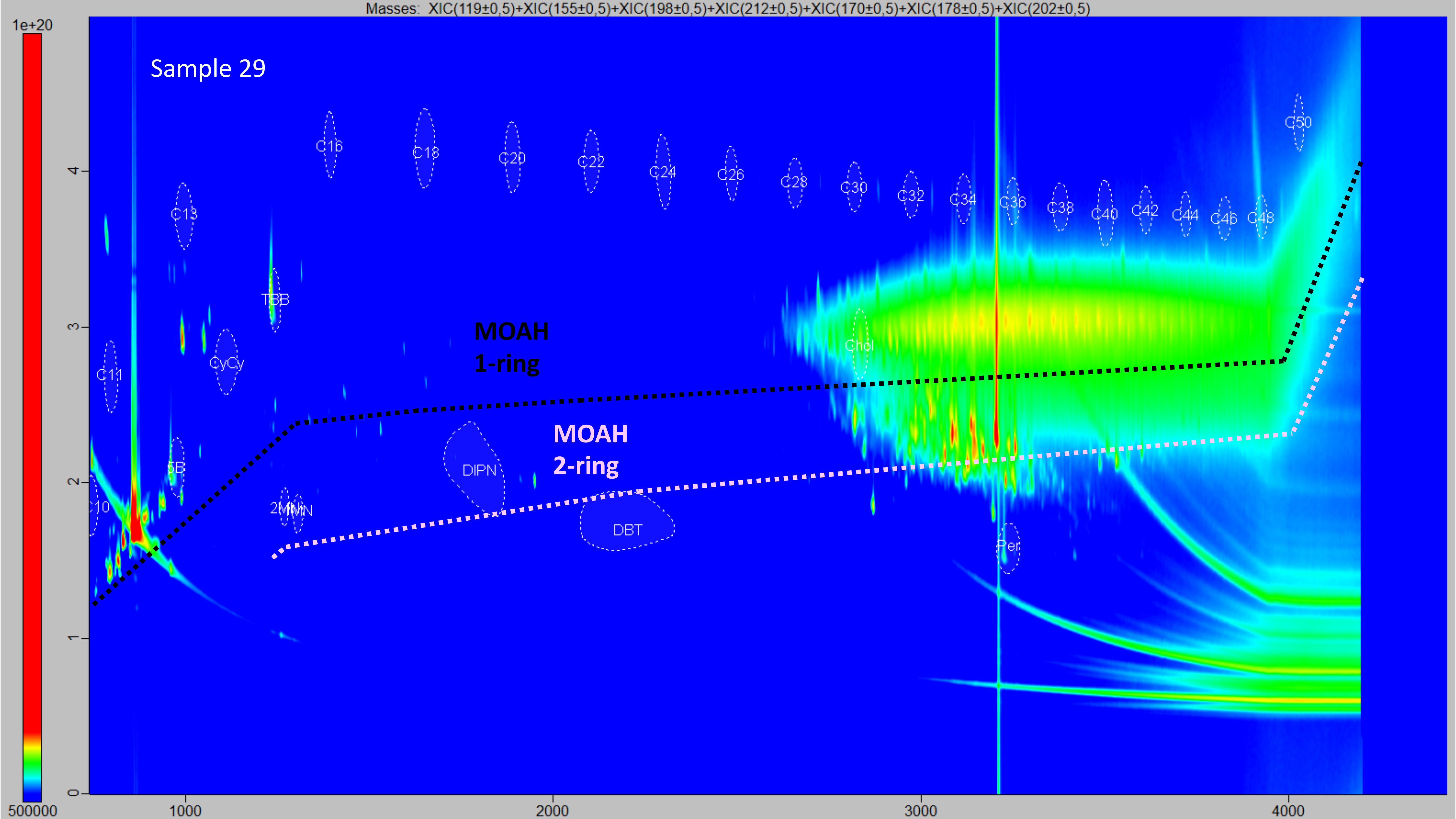
Masses: XIC(119±0,5)+XIC(155±0,5)+XIC(198±0,5)+XIC(212±0,5)+XIC(170±0,5)+XIC(178±0,5)+XIC(202±0,5)
1e+20
500000
Sample 29
MOAH
1-ring
MOAH
2-ring
C10
C11
C13
C16
C18
C20
C22
C24
C26
C28
C30
C32
C34
C36
C38
C40
C42
C44
C46
C48
C50
TBB
CyCy
5B
2MN
1MN
DIPN
DBT
Chol
Per
0
1
2
3
4
1000
2000
3000
4000

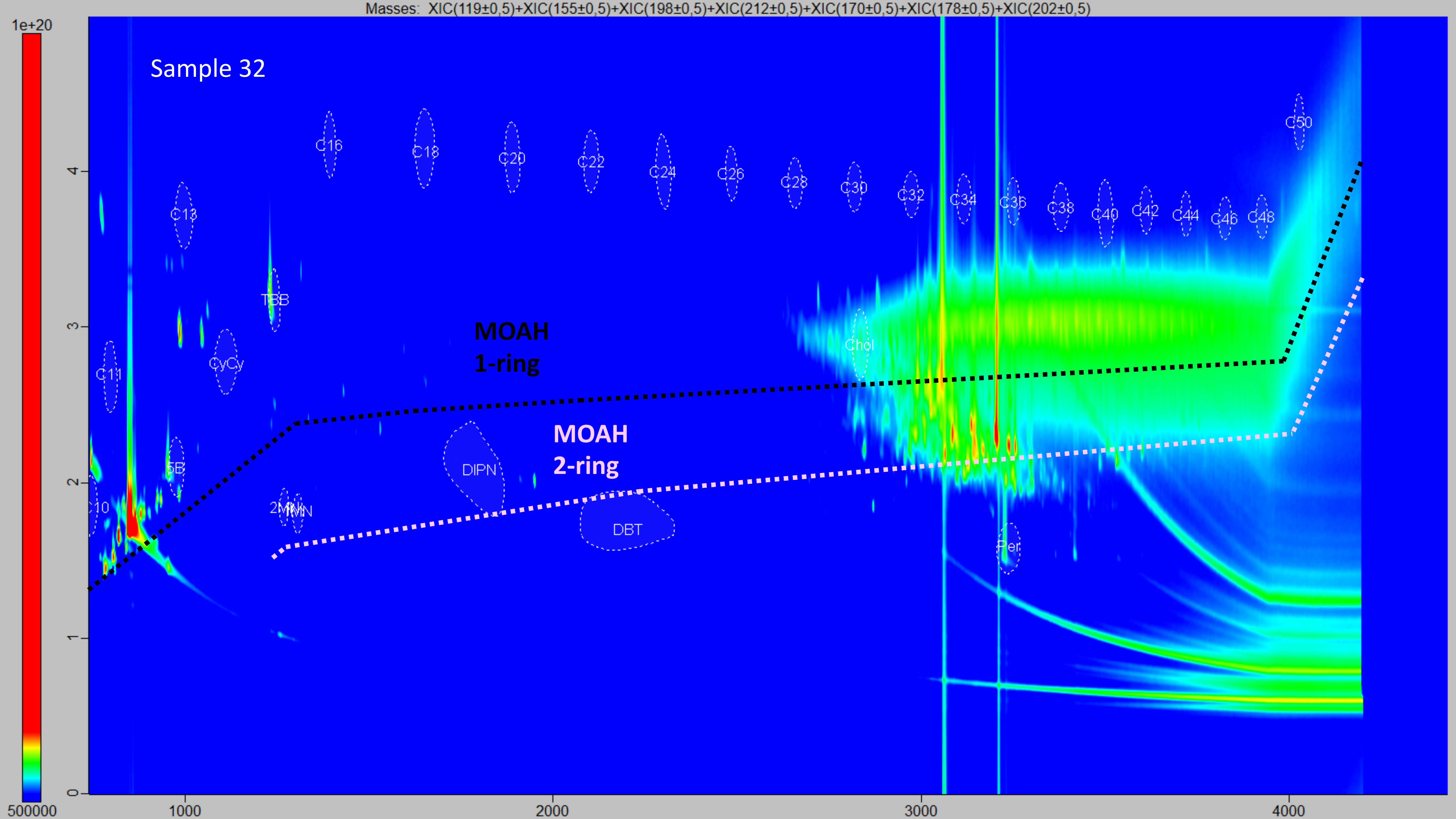
Masses: XIC(119±0,5)+XIC(155±0,5)+XIC(198±0,5)+XIC(212±0,5)+XIC(170±0,5)+XIC(178±0,5)+XIC(202±0,5)
1e+20
500000
Sample 32
C16
C18
C20
C22
C24
C26
C28
C30
C32
C34
C36
C38
C40
C42
C44
C46
C48
C50
C13
C11
C10
TBB
CyCy
5B
2MN
1MN
DIPN
DBT
Chol
Per
MOAH
1-ring
MOAH
2-ring
0
1
2
3
4
1000
2000
3000
4000

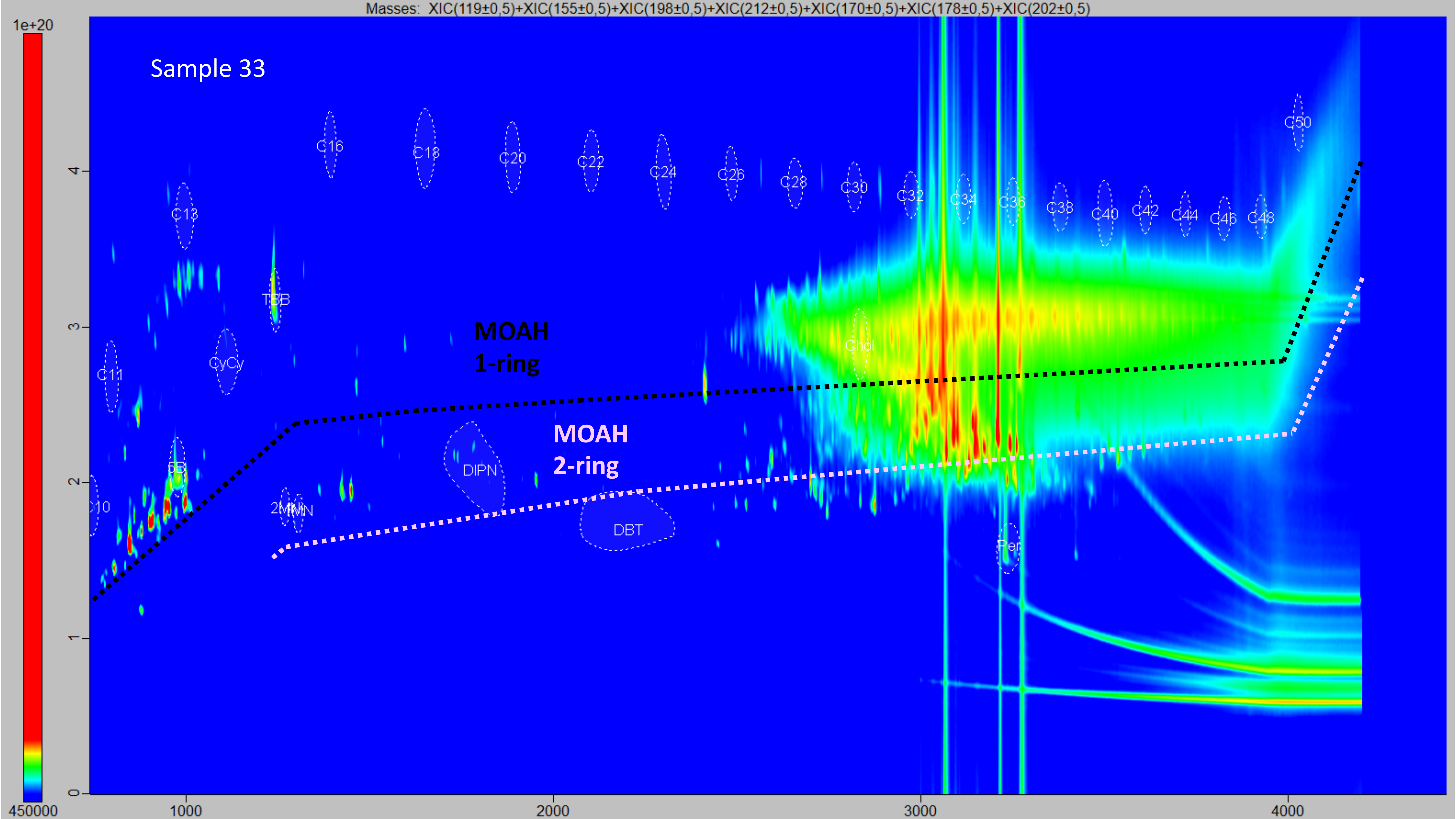

Masses: XIC(119±0,5)+XIC(155±0,5)+XIC(198±0,5)+XIC(212±0,5)+XIC(170±0,5)+XIC(178±0,5)+XIC(202±0,5)
Sample 33
1e+20
450000
MOAH
1-ring
MOAH
2-ring
C10
C11
C13
C16
C18
C20
C22
C24
C26
C28
C30
C32
C34
C36
C38
C40
C42
C44
C46
C48
C50
TBB
CyCy
DIPN
DBT
Chol
Per
0
1
2
3
4
1000
2000
3000
4000

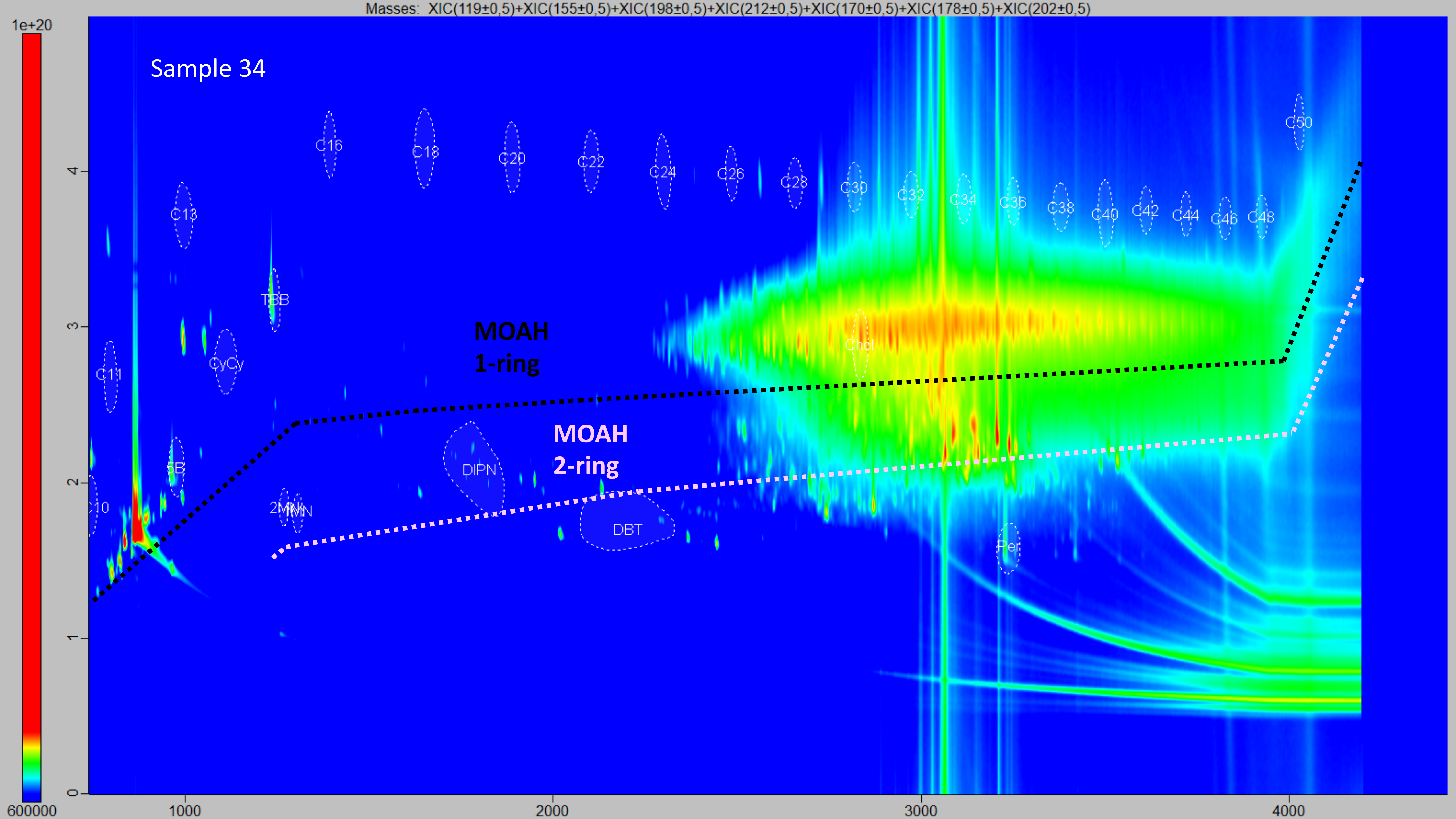
Masses: XIC(119±0,5)+XIC(155±0,5)+XIC(198±0,5)+XIC(212±0,5)+XIC(170±0,5)+XIC(178±0,5)+XIC(202±0,5)
1e+20
600000
Sample 34
C16
C18
C20
C22
C24
C26
C28
C30
C32
C34
C36
C38
C40
C42
C44
C46
C48
C50
C13
C11
C10
TBB
CyCy
5B
2MN
1MN
DIPN
DBT
Chol
Per
MOAH
1-ring
MOAH
2-ring
0
1
2
3
4
1000
2000
3000
4000

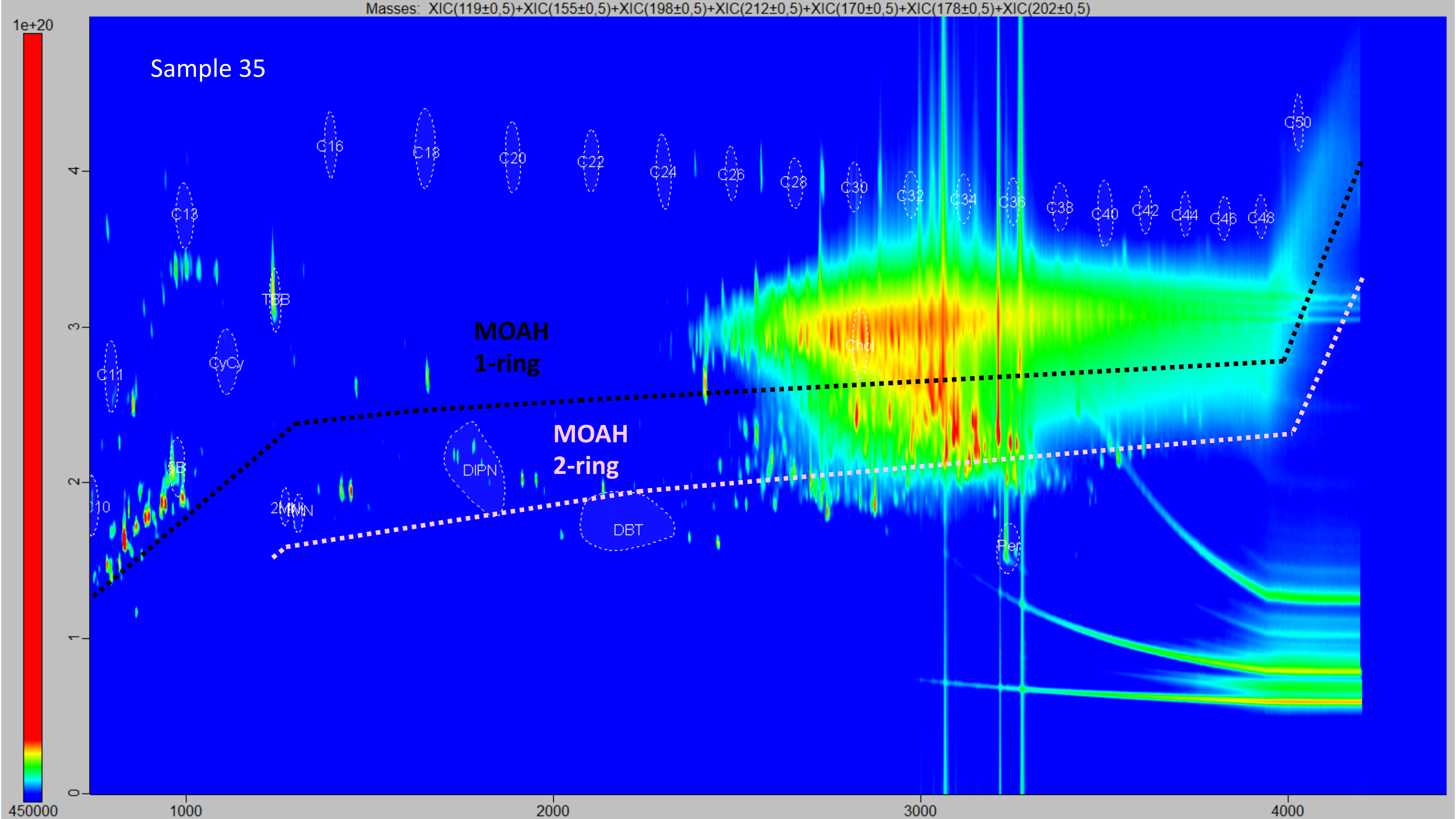

Masses: XIC(119±0,5)+XIC(155±0,5)+XIC(198±0,5)+XIC(212±0,5)+XIC(170±0,5)+XIC(178±0,5)+XIC(202±0,5)
1e+20
450000
Sample 35
C16
C18
C20
C22
C24
C26
C28
C30
C32
C34
C36
C38
C40
C42
C44
C46
C48
C50
C13
TBB
C11
CyCy
Chol
MOAH
1-ring
MOAH
2-ring
DIPN
DBT
Per
2MN
1MN
5B
C10
4
3
2
1
0
1000
2000
3000
4000

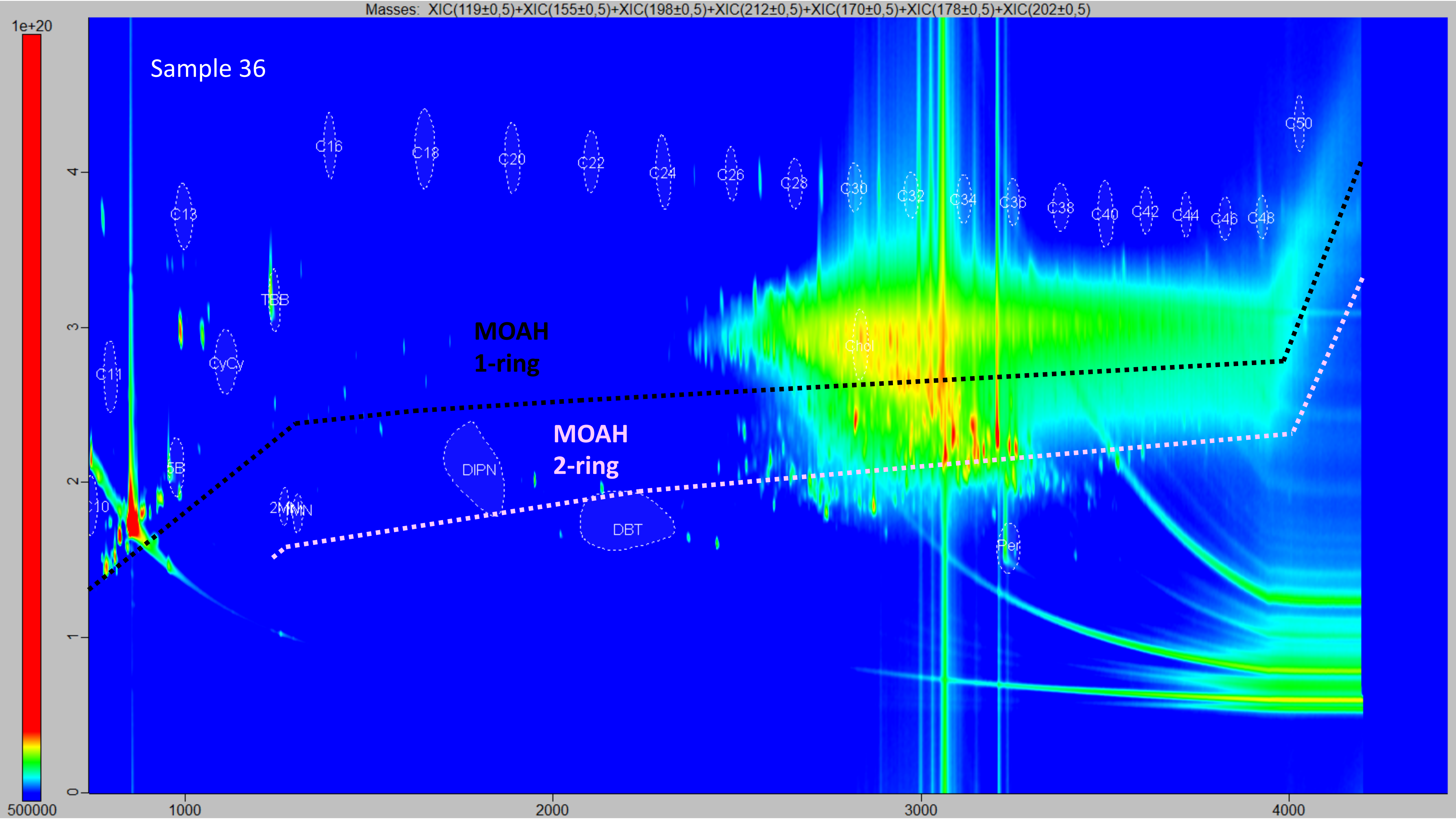
Masses: XIC(119±0,5)+XIC(155±0,5)+XIC(198±0,5)+XIC(212±0,5)+XIC(170±0,5)+XIC(178±0,5)+XIC(202±0,5)
1e+20
500000
Sample 36
MOAH
1-ring
MOAH
2-ring
C11
C13
C16
C18
C20
C22
C24
C26
C28
C30
C32
C34
C36
C38
C40
C42
C44
C46
C48
C50
TBB
CyCy
5B
C10
2MN
1MN
DIPN
DBT
Chol
Per
0
1
2
3
4
1000
2000
3000
4000

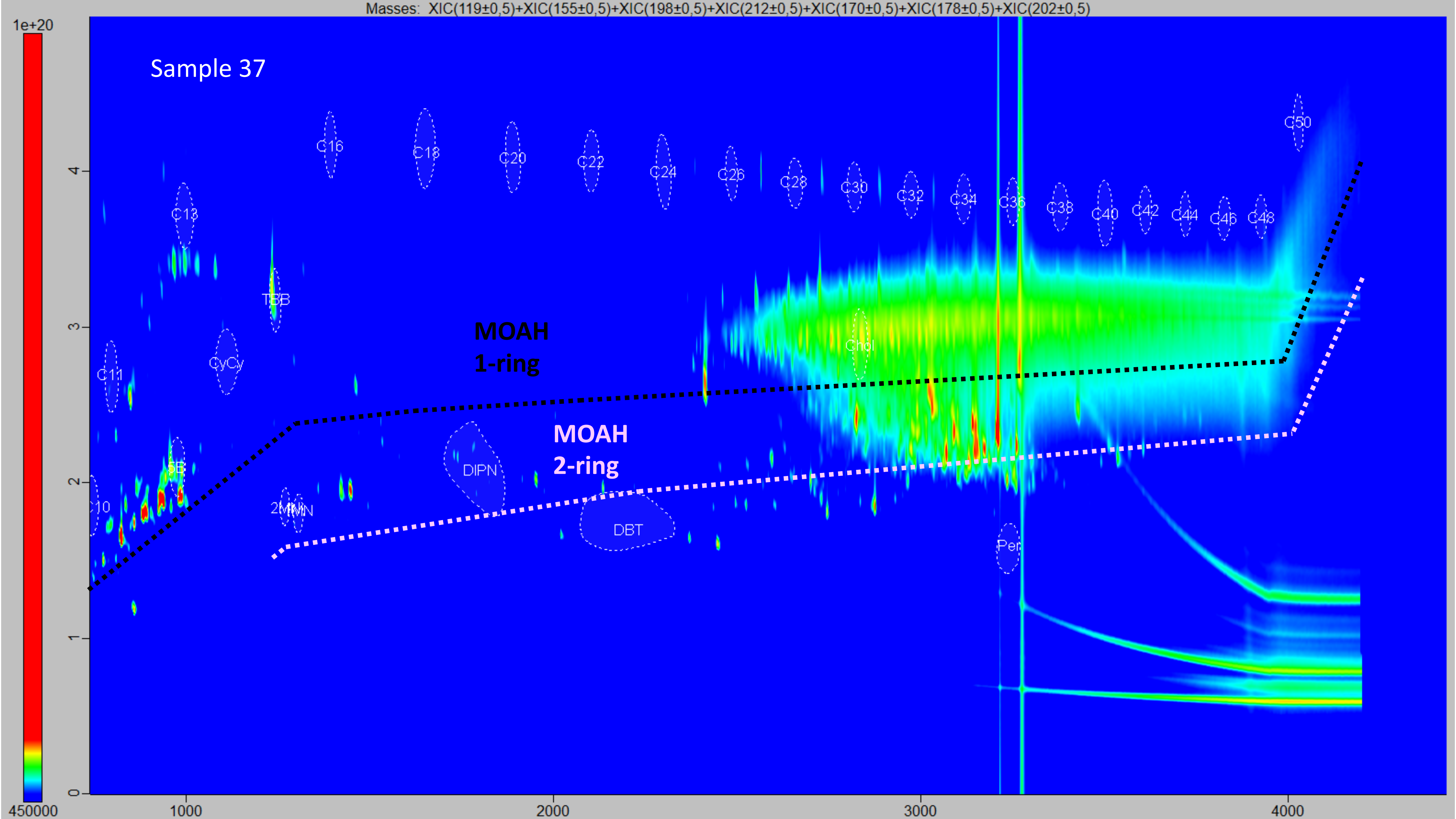
Masses: XIC(119±0,5)+XIC(155±0,5)+XIC(198±0,5)+XIC(212±0,5)+XIC(170±0,5)+XIC(178±0,5)+XIC(202±0,5)
1e+20
450000
Sample 37
MOAH 1-ring
MOAH 2-ring
C16
C18
C20
C22
C24
C26
C28
C30
C32
C34
C36
C38
C40
C42
C44
C46
C48
C50
C13
C11
C10
TBB
CyCy
5B
DIPN
DBT
Chol
Per
0
1
2
3
4
1000
2000
3000
4000

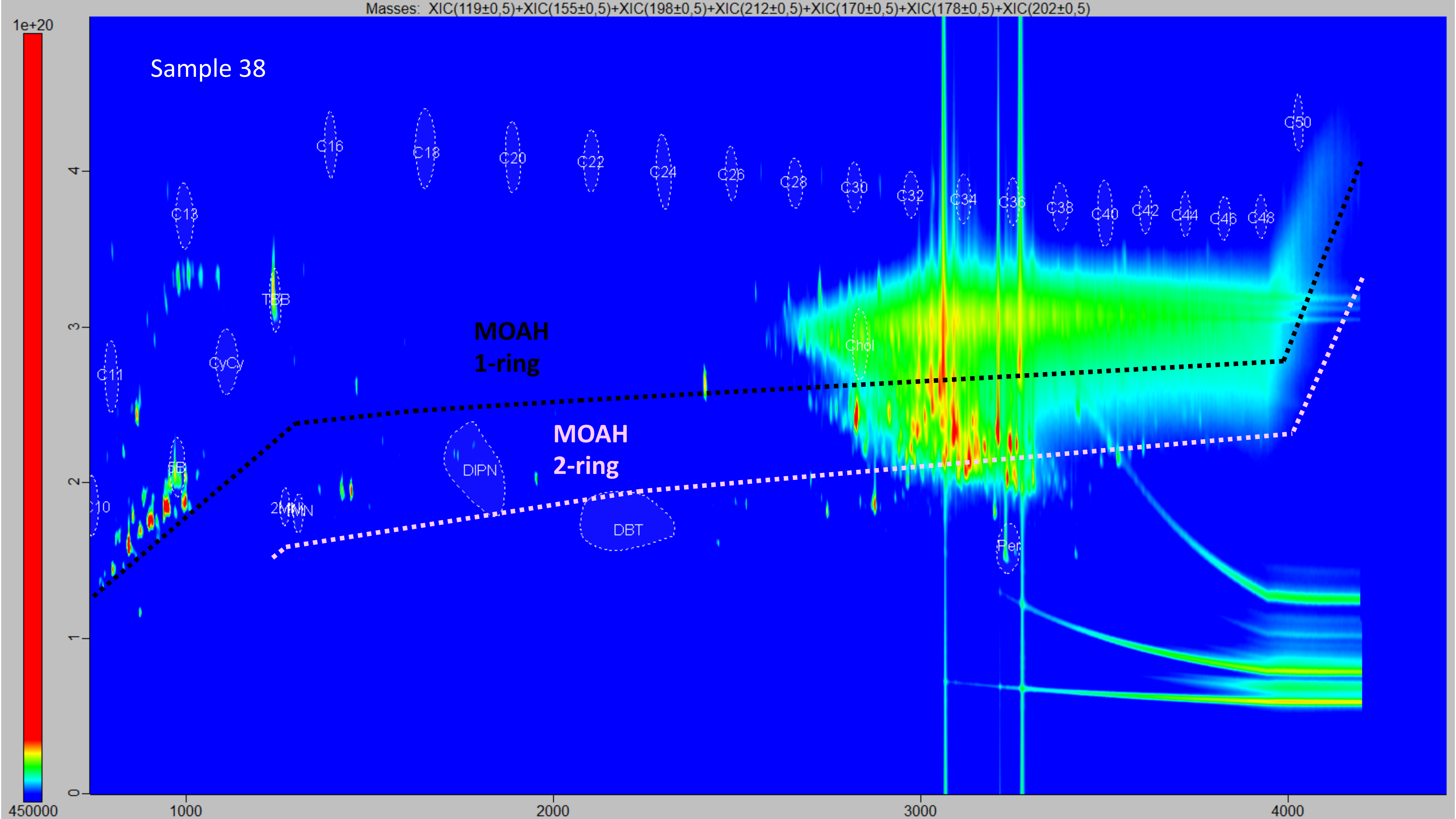
Masses: XIC(119±0,5)+XIC(155±0,5)+XIC(198±0,5)+XIC(212±0,5)+XIC(170±0,5)+XIC(178±0,5)+XIC(202±0,5)
1e+20
450000
Sample 38
MOAH
1-ring
MOAH
2-ring
C10
C11
C13
C16
C18
C20
C22
C24
C26
C28
C30
C32
C34
C36
C38
C40
C42
C44
C46
C48
C50
TBB
CyCy
5B
DIPN
DBT
Chol
Per
0
1
2
3
4
1000
2000
3000
4000

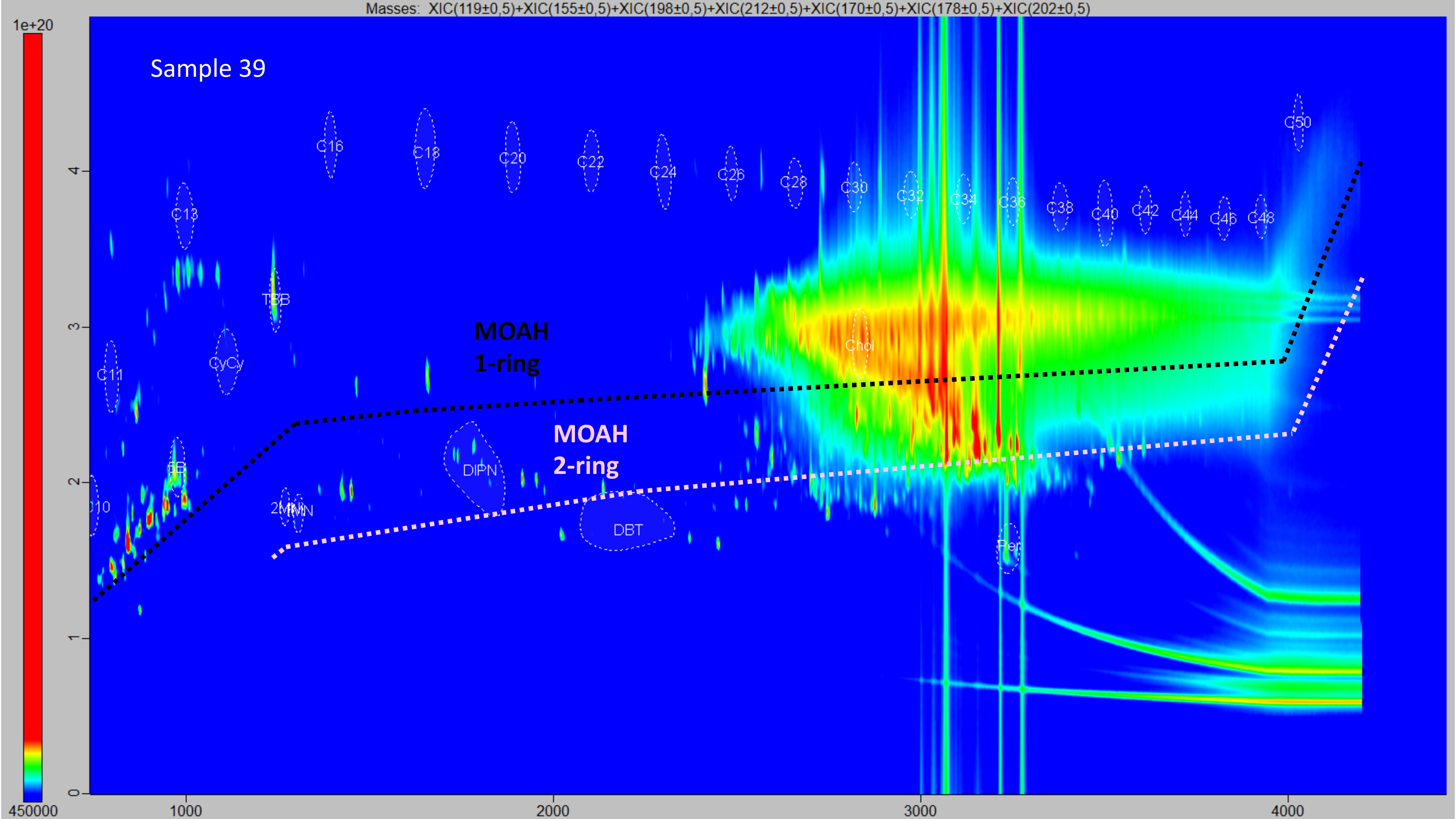

Masses: XIC(119±0,5)+XIC(155±0,5)+XIC(198±0,5)+XIC(212±0,5)+XIC(170±0,5)+XIC(178±0,5)+XIC(202±0,5)
1e+20
450000
Sample 39
MOAH
1-ring
MOAH
2-ring
C11
C13
C16
C18
C20
C22
C24
C26
C28
C30
C32
C34
C36
C38
C40
C42
C44
C46
C48
C50
TBB
CyCy
DIPN
DBT
Chol
Per
0
1
2
3
4
1000
2000
3000
4000

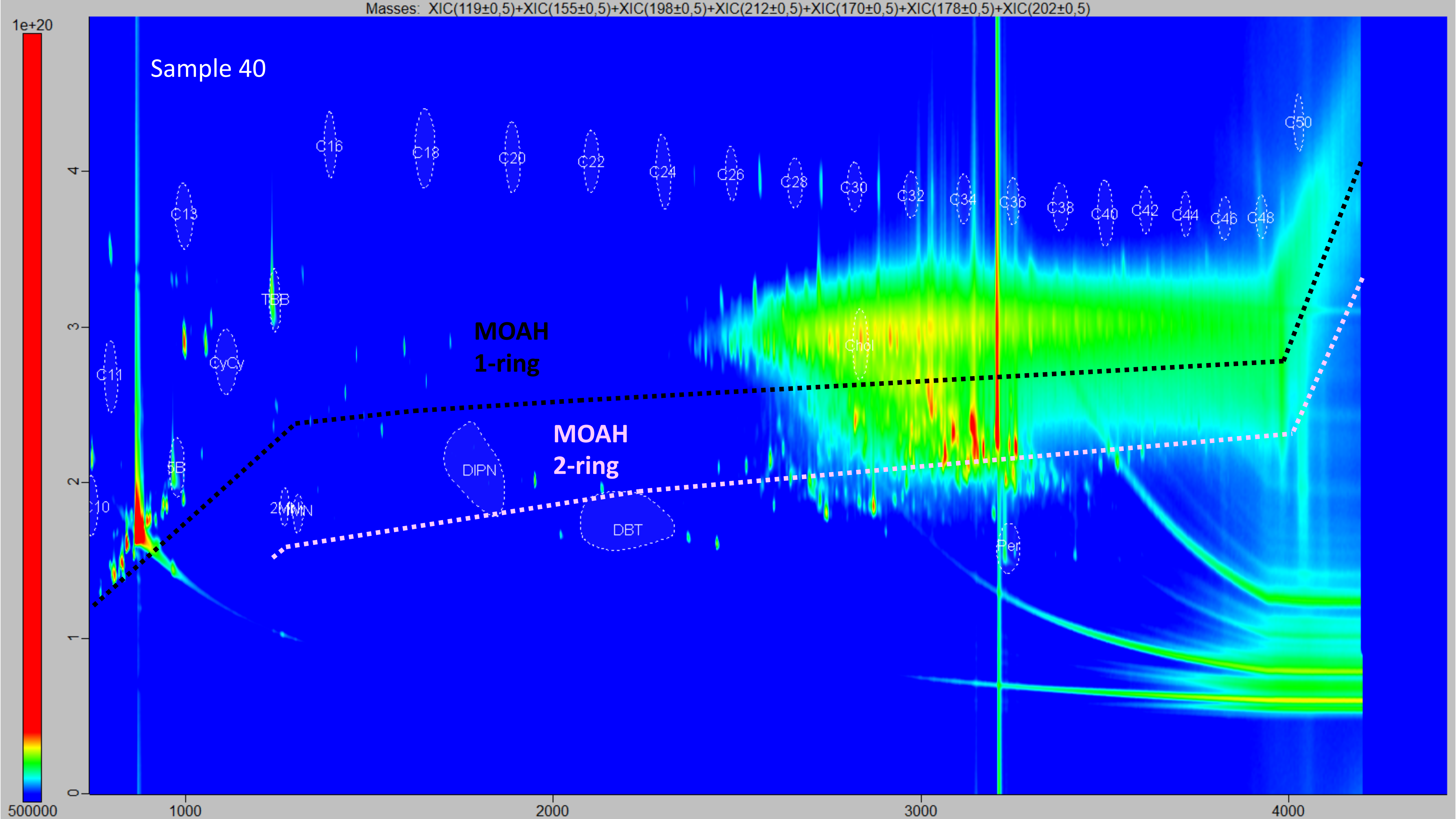
Masses: XIC(119±0,5)+XIC(155±0,5)+XIC(198±0,5)+XIC(212±0,5)+XIC(170±0,5)+XIC(178±0,5)+XIC(202±0,5)
1e+20
500000
Sample 40
C10
C11
C13
C16
C18
C20
C22
C24
C26
C28
C30
C32
C34
C36
C38
C40
C42
C44
C46
C48
C50
TBB
CyCy
5B
2MN
1MN
DIPN
DBT
Chol
Per
MOAH
1-ring
MOAH
2-ring
0
1
2
3
4
1000
2000
3000
4000

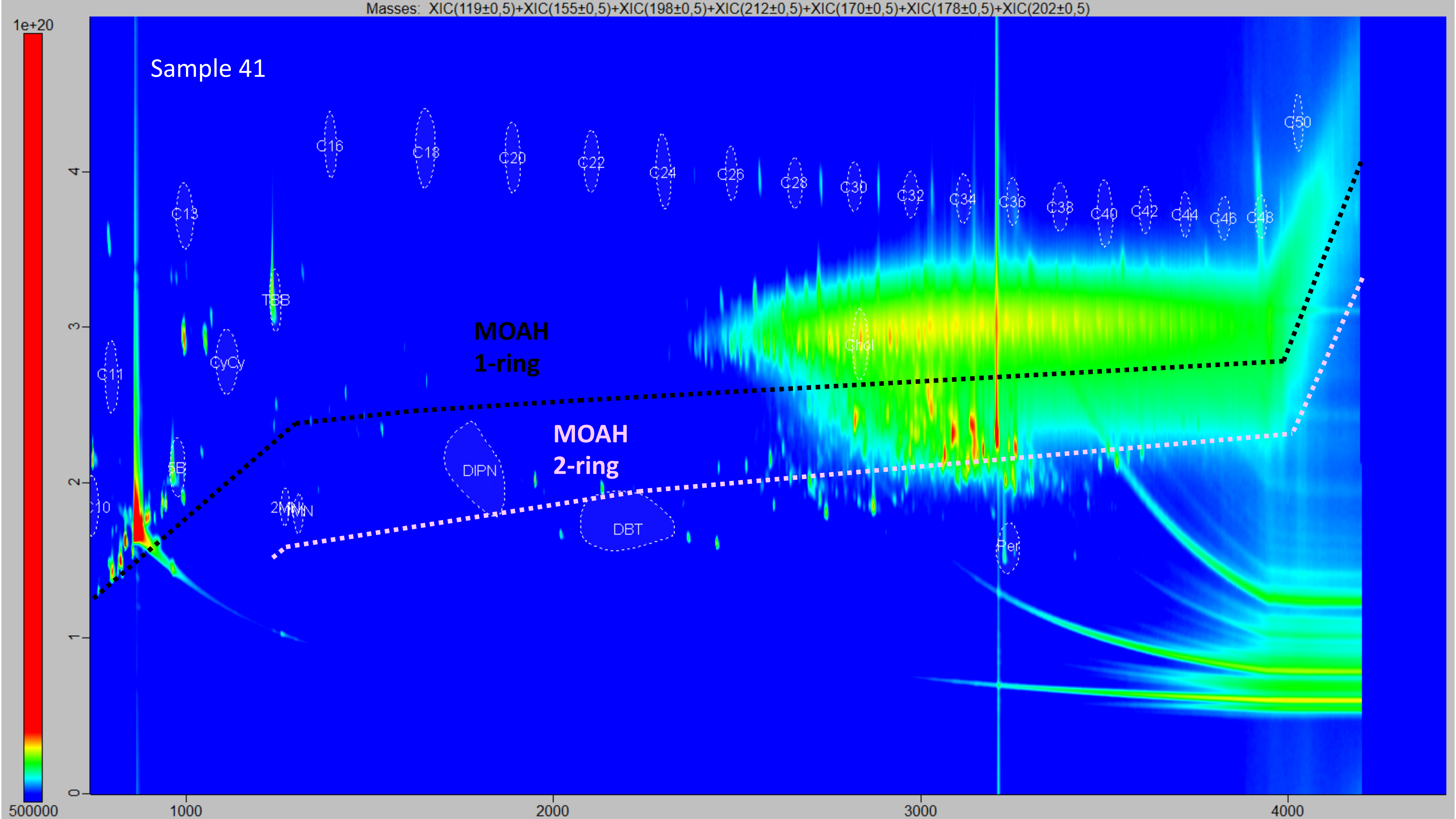

Masses: XIC(119±0,5)+XIC(155±0,5)+XIC(198±0,5)+XIC(212±0,5)+XIC(170±0,5)+XIC(178±0,5)+XIC(202±0,5)
Sample 41
1e+20
500000
MOAH
1-ring
MOAH
2-ring
C11
C13
C16
C18
C20
C22
C24
C26
C28
C30
C32
C34
C36
C38
C40
C42
C44
C46
C48
C50
TBB
CyCy
5B
DIPN
DBT
Chol
Per
0
1
2
3
4
1000
2000
3000
4000

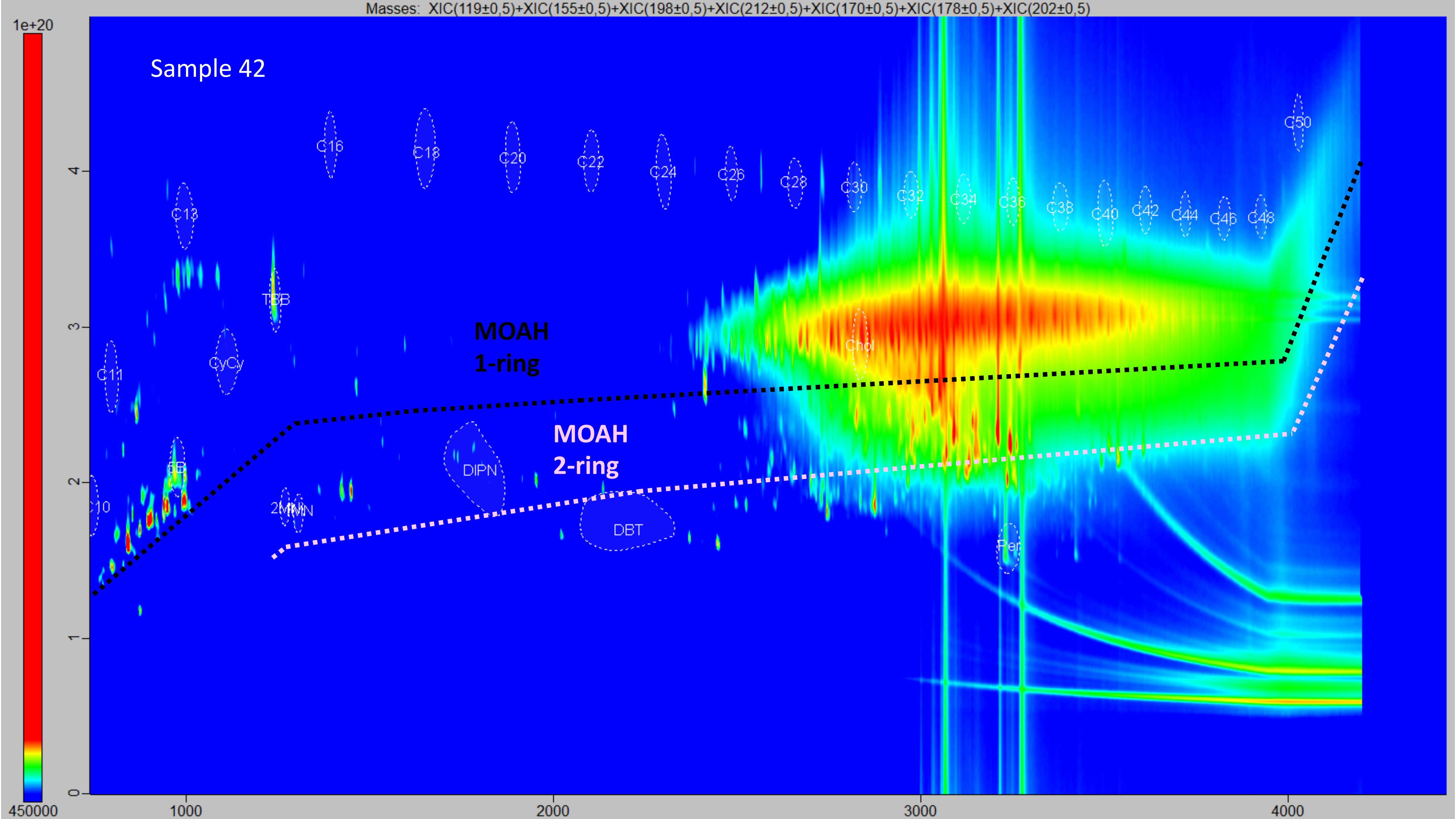
Masses: XIC(119±0,5)+XIC(155±0,5)+XIC(198±0,5)+XIC(212±0,5)+XIC(170±0,5)+XIC(178±0,5)+XIC(202±0,5)
1e+20
450000
Sample 42
MOAH
1-ring
MOAH
2-ring
C10
C11
C13
C16
C18
C20
C22
C24
C26
C28
C30
C32
C34
C36
C38
C40
C42
C44
C46
C48
C50
TBB
CyCy
DIPN
DBT
Chol
Per
0
1
2
3
4
1000
2000
3000
4000

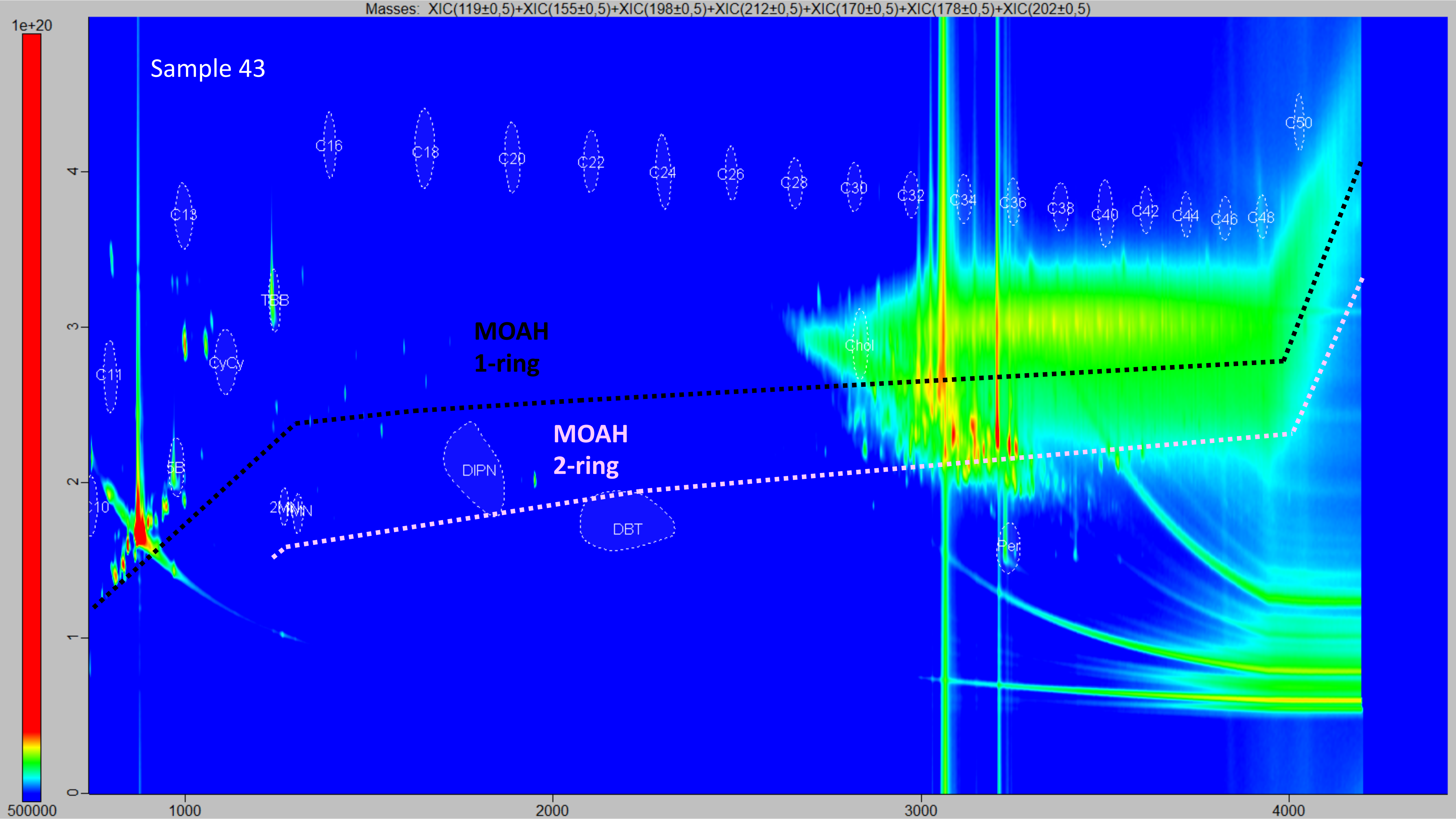
Masses: XIC(119±0,5)+XIC(155±0,5)+XIC(198±0,5)+XIC(212±0,5)+XIC(170±0,5)+XIC(178±0,5)+XIC(202±0,5)
1e+20
500000
Sample 43
C16
C18
C20
C22
C24
C26
C28
C30
C32
C34
C36
C38
C40
C42
C44
C46
C48
C50
C13
C11
C10
TBB
CyCy
5B
Chol
MOAH
1-ring
MOAH
2-ring
DIPN
DBT
Per
4
3
2
1
0
1000
2000
3000
4000

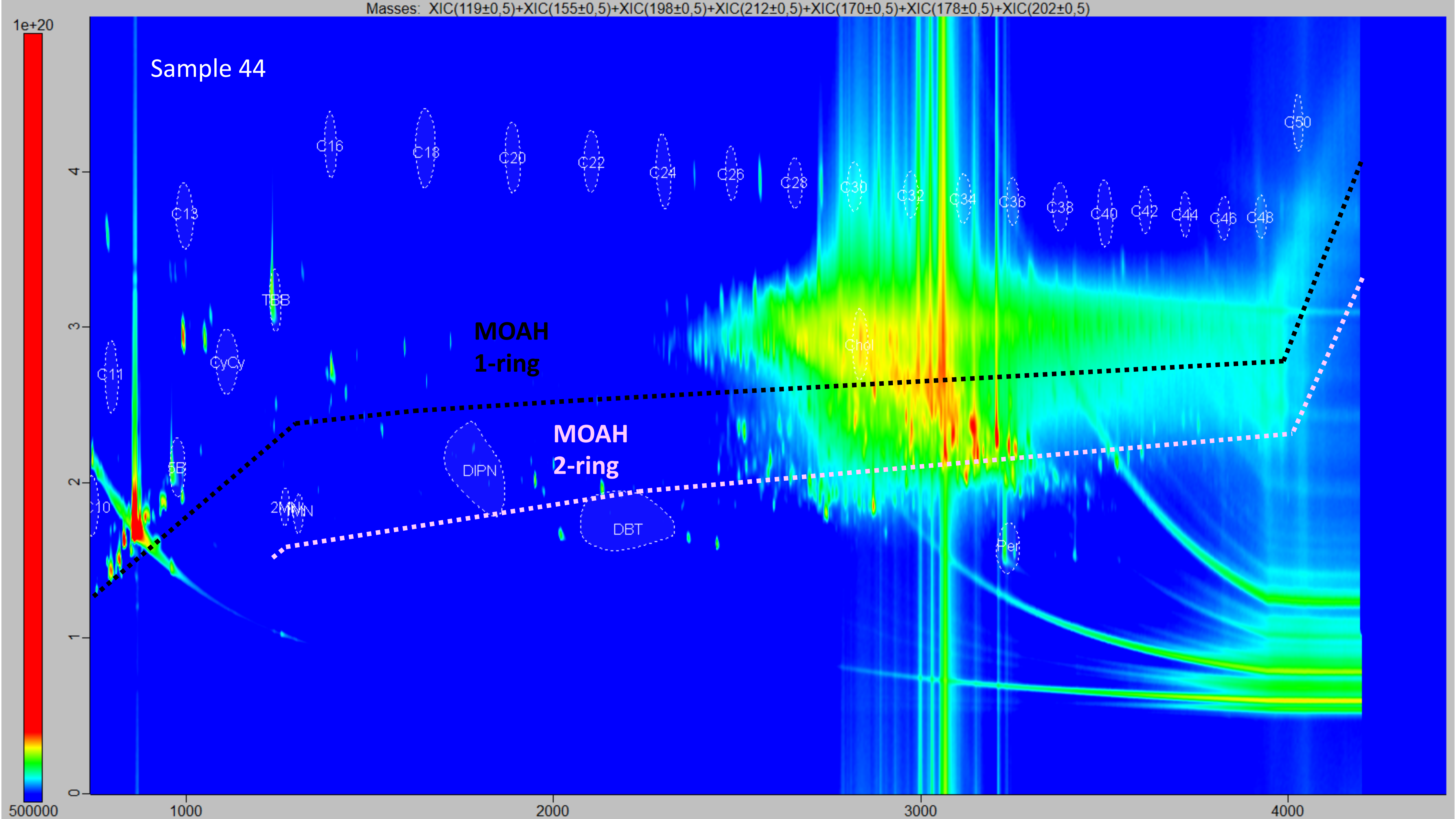

Masses: XIC(119±0,5)+XIC(155±0,5)+XIC(198±0,5)+XIC(212±0,5)+XIC(170±0,5)+XIC(178±0,5)+XIC(202±0,5)
1e+20
500000
Sample 44
MOAH
1-ring
MOAH
2-ring
C10
C11
C13
C16
C18
C20
C22
C24
C26
C28
C30
C32
C34
C36
C38
C40
C42
C44
C46
C48
C50
TBB
CyCy
5B
2MN
DIPN
DBT
Chol
Per
0
1
2
3
4
1000
2000
3000
4000

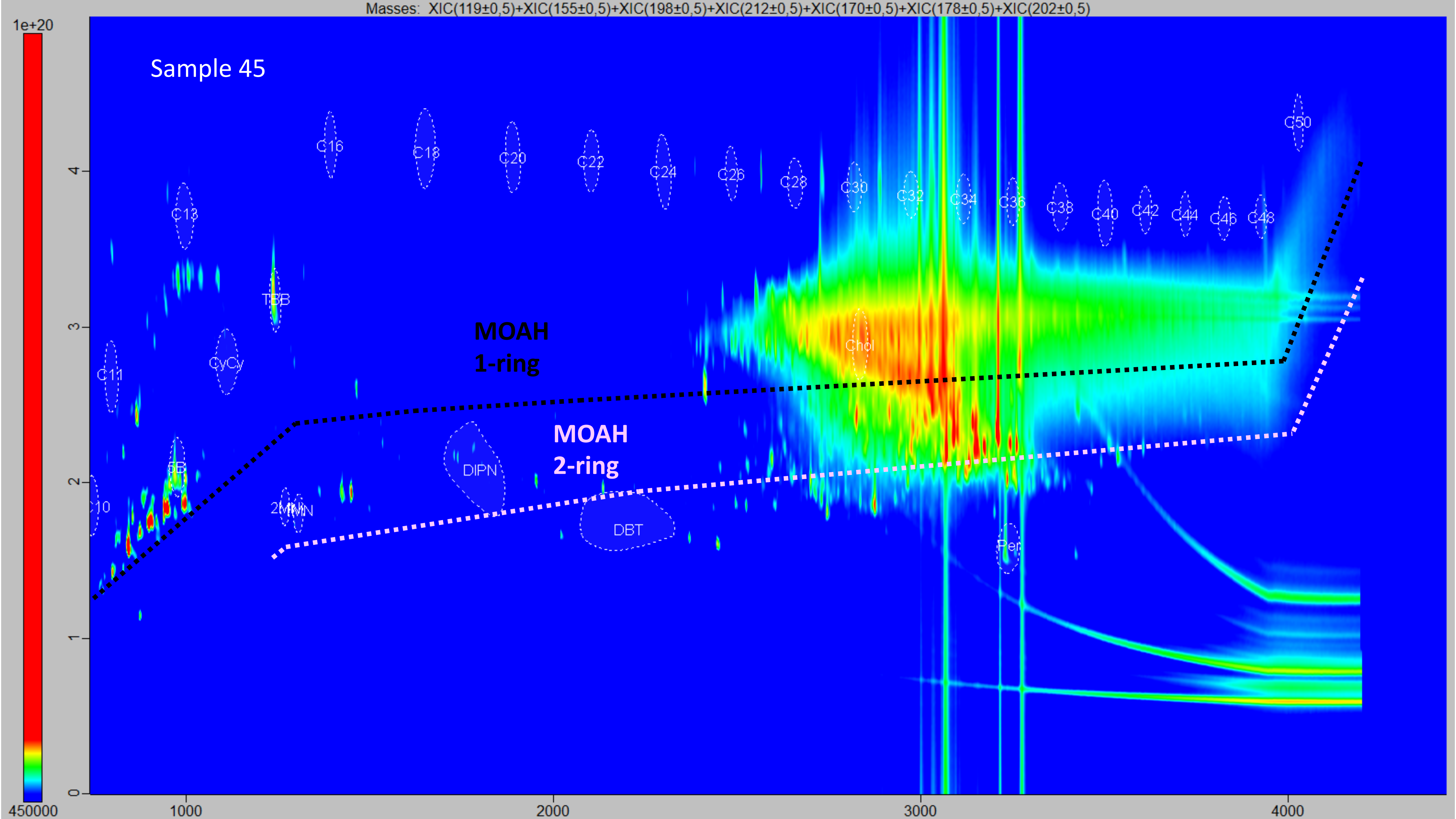

Masses: XIC(119±0,5)+XIC(155±0,5)+XIC(198±0,5)+XIC(212±0,5)+XIC(170±0,5)+XIC(178±0,5)+XIC(202±0,5)
Sample 45
1e+20
450000
MOAH
1-ring
MOAH
2-ring
C11
C13
C16
C18
C20
C22
C24
C26
C28
C30
C32
C34
C36
C38
C40
C42
C44
C46
C48
C50
TBB
CyCy
DIPN
DBT
Chol
Per
0
1
2
3
4
1000
2000
3000
4000

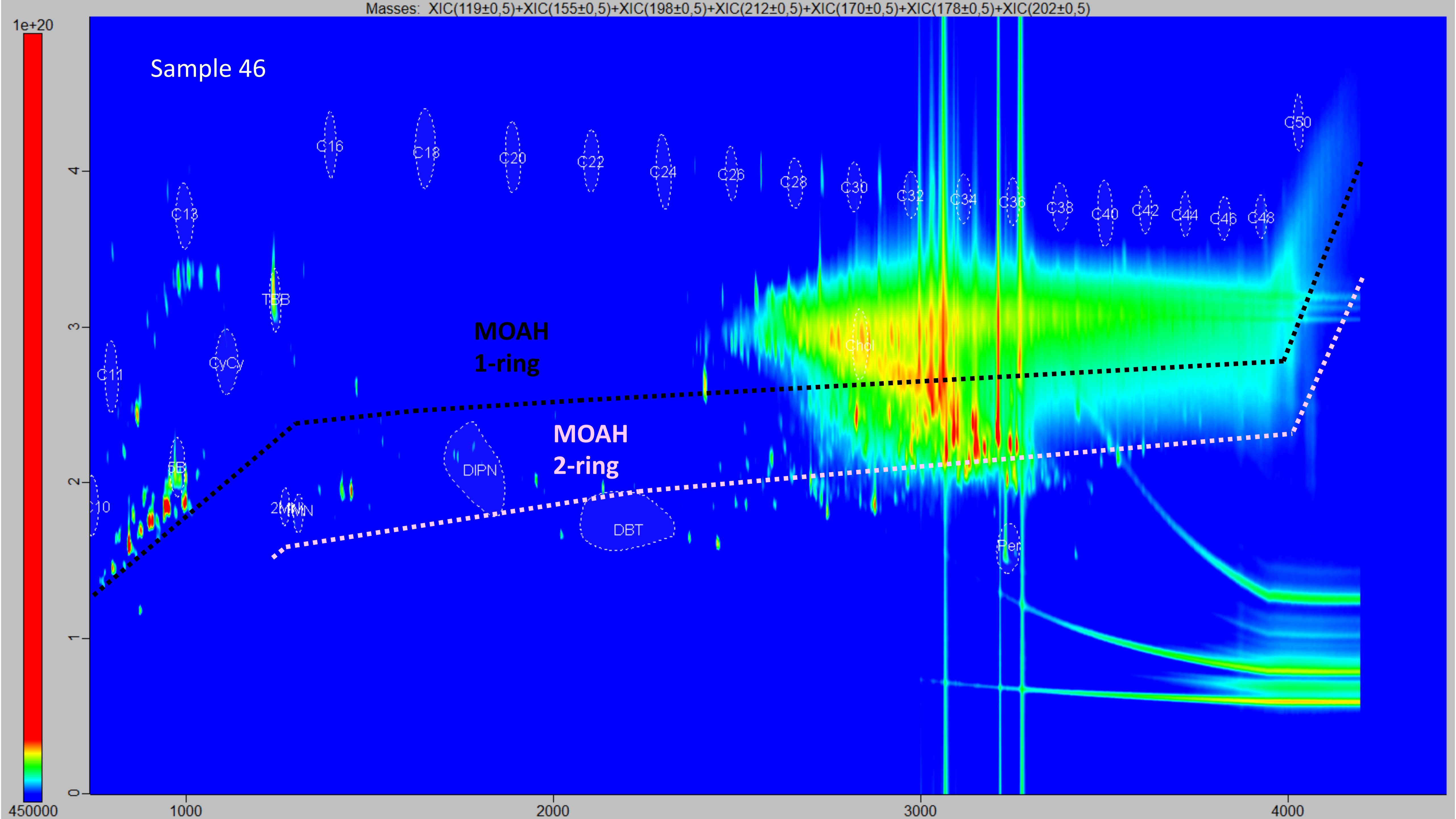
Masses: XIC(119±0,5)+XIC(155±0,5)+XIC(198±0,5)+XIC(212±0,5)+XIC(170±0,5)+XIC(178±0,5)+XIC(202±0,5)
1e+20
450000
Sample 46
C16
C18
C20
C22
C24
C26
C28
C30
C32
C34
C36
C38
C40
C42
C44
C46
C48
C50
C13
C11
C10
TBB
CyCy
5B
2MN
1MN
DIPN
DBT
Chol
Per
MOAH
1-ring
MOAH
2-ring
0
1
2
3
4
1000
2000
3000
4000

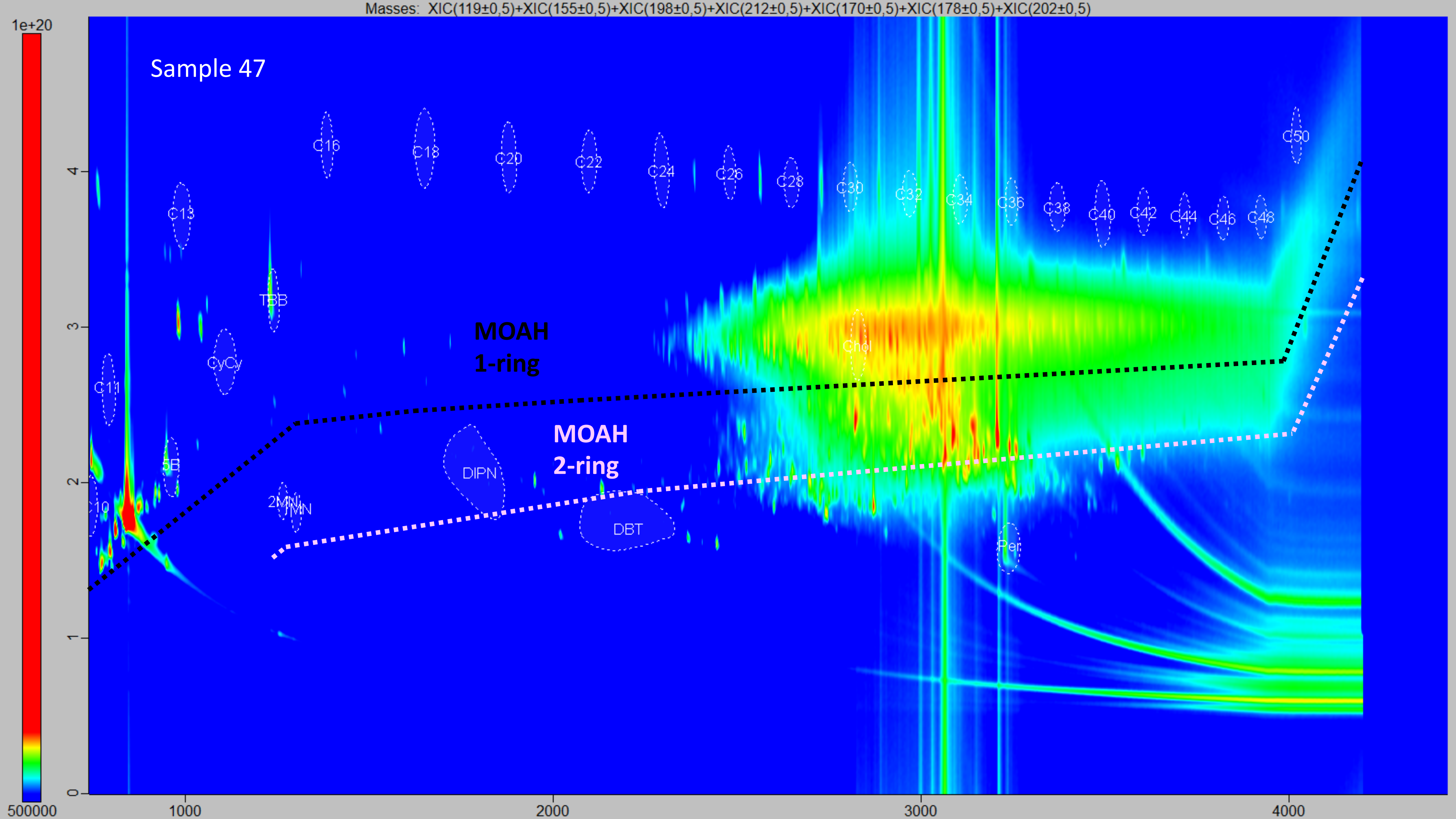

Masses: XIC(119±0,5)+XIC(155±0,5)+XIC(198±0,5)+XIC(212±0,5)+XIC(170±0,5)+XIC(178±0,5)+XIC(202±0,5)
Sample 47
MOAH
1-ring
MOAH
2-ring
C16
C18
C20
C22
C24
C26
C28
C30
C32
C34
C36
C38
C40
C42
C44
C46
C48
C50
C13
C11
C10
TBB
CyCy
5B
2MN
1MN
DIPN
DBT
Chol
Per
1e+20
500000
0
1
2
3
4
1000
2000
3000
4000

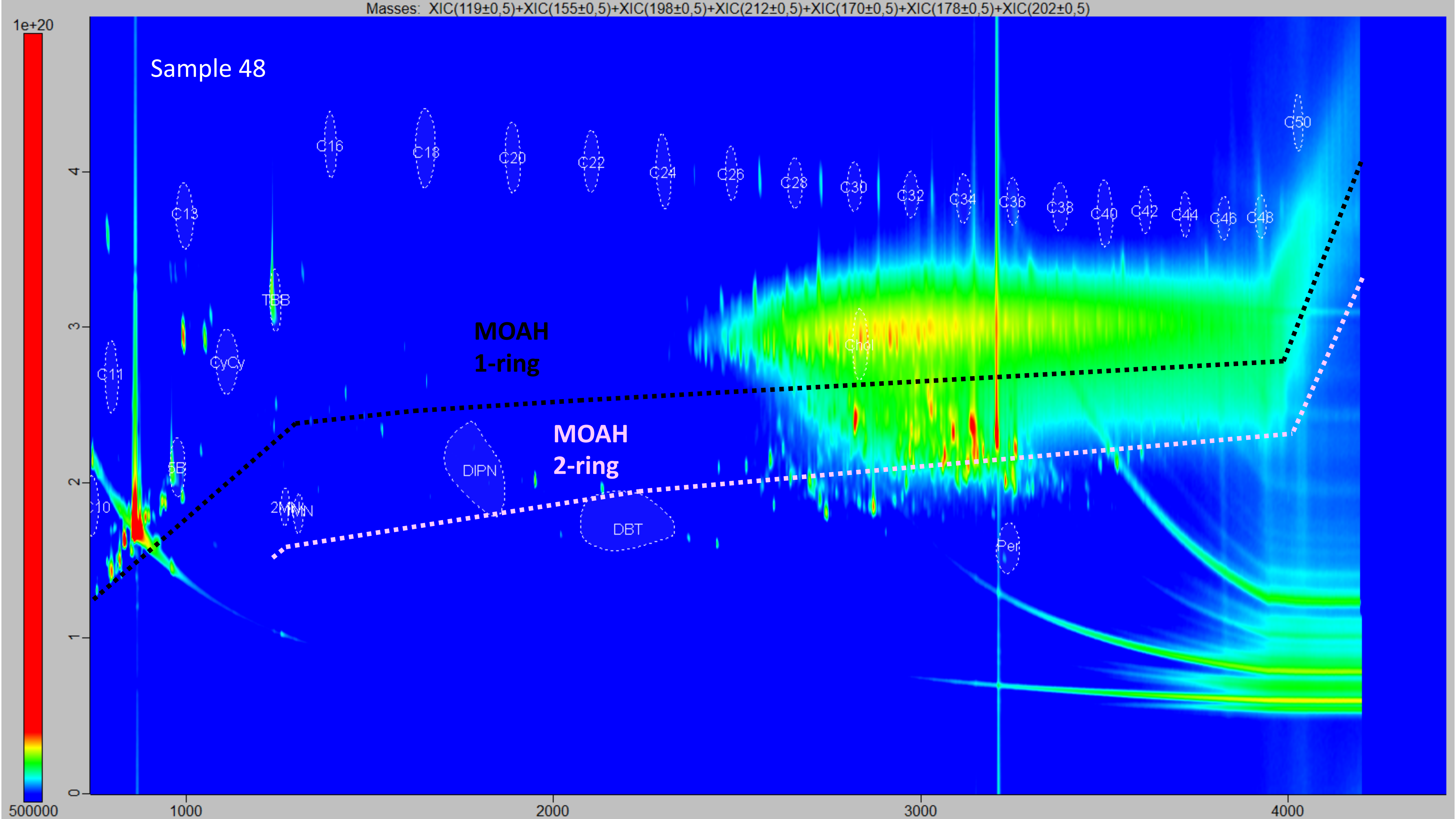

Masses: XIC(119±0,5)+XIC(155±0,5)+XIC(198±0,5)+XIC(212±0,5)+XIC(170±0,5)+XIC(178±0,5)+XIC(202±0,5)
1e+20
500000
Sample 48
C16
C18
C20
C22
C24
C26
C28
C30
C32
C34
C36
C38
C40
C42
C44
C46
C48
C50
C13
C11
C10
TBB
CyCy
5B
MOAH 1-ring
MOAH 2-ring
Chol
DIPN
DBT
Per
0
1
2
3
4
1000
2000
3000
4000

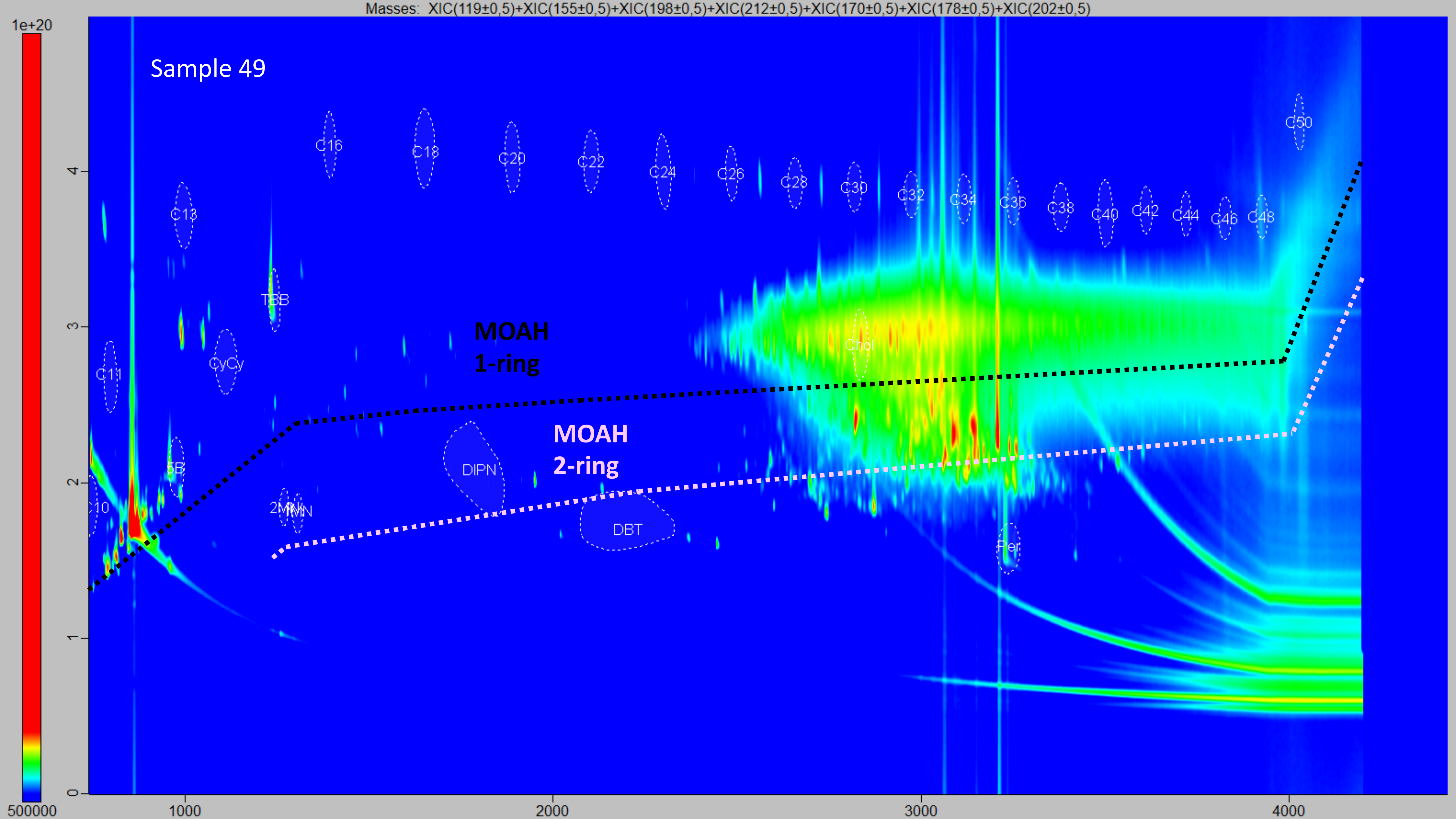

Masses: XIC(119±0,5)+XIC(155±0,5)+XIC(198±0,5)+XIC(212±0,5)+XIC(170±0,5)+XIC(178±0,5)+XIC(202±0,5)
1e+20
500000
Sample 49
MOAH
1-ring
MOAH
2-ring
C10
C11
C13
5B
CyCy
TBB
2MN
1MN
C16
C18
C20
C22
C24
C26
C28
C30
C32
C34
C36
C38
C40
C42
C44
C46
C48
C50
DIPN
DBT
Chol
Per
1000
2000
3000
4000
0
1
2
3
4

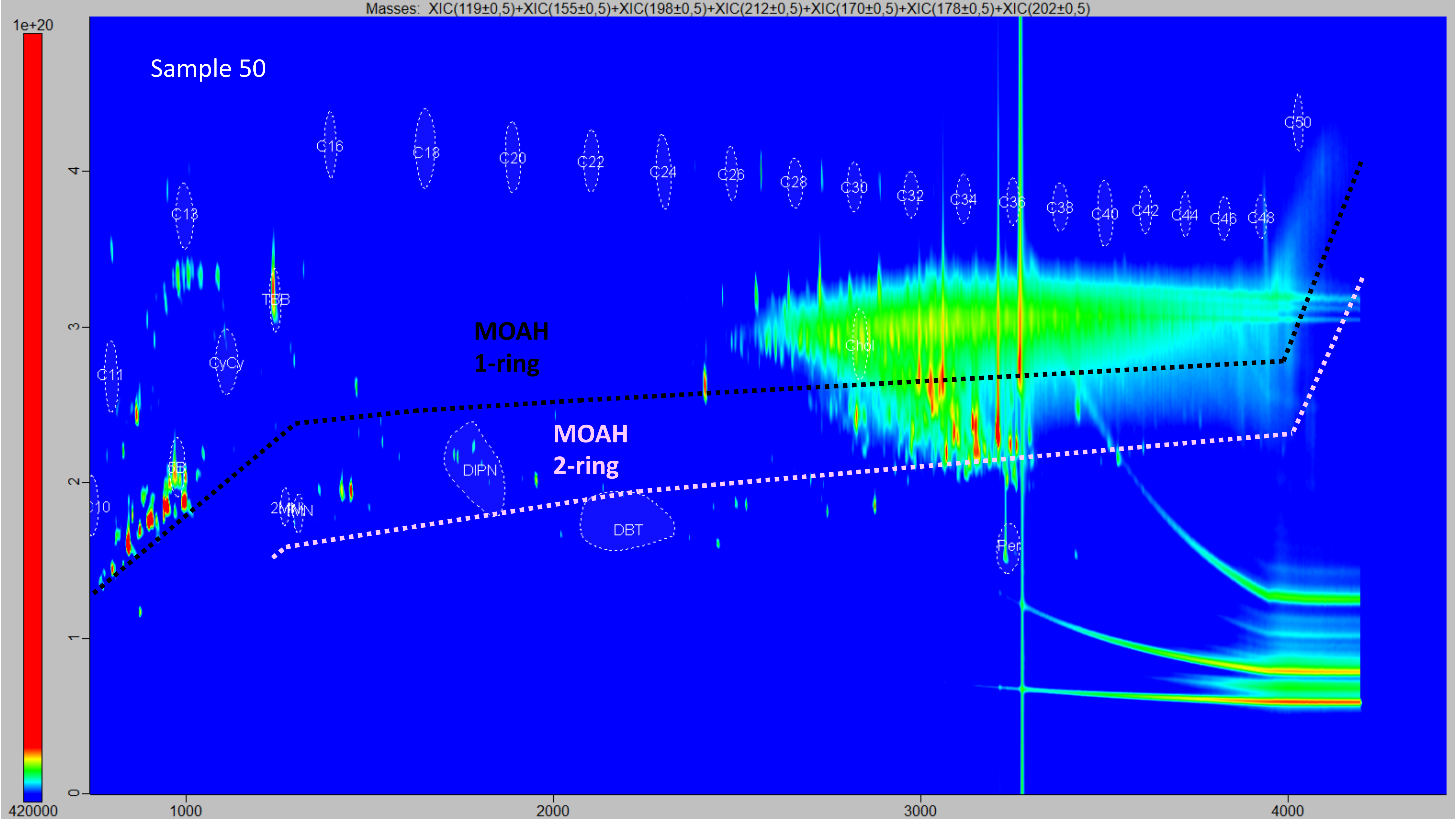
Masses: XIC(119±0,5)+XIC(155±0,5)+XIC(198±0,5)+XIC(212±0,5)+XIC(170±0,5)+XIC(178±0,5)+XIC(202±0,5)
1e+20
420000
Sample 50
MOAH
1-ring
MOAH
2-ring
C10
C11
C13
C16
C18
C20
C22
C24
C26
C28
C30
C32
C34
C36
C38
C40
C42
C44
C46
C48
C50
TBB
CyCy
DIPN
DBT
Chol
Per
0
1
2
3
4
1000
2000
3000
4000

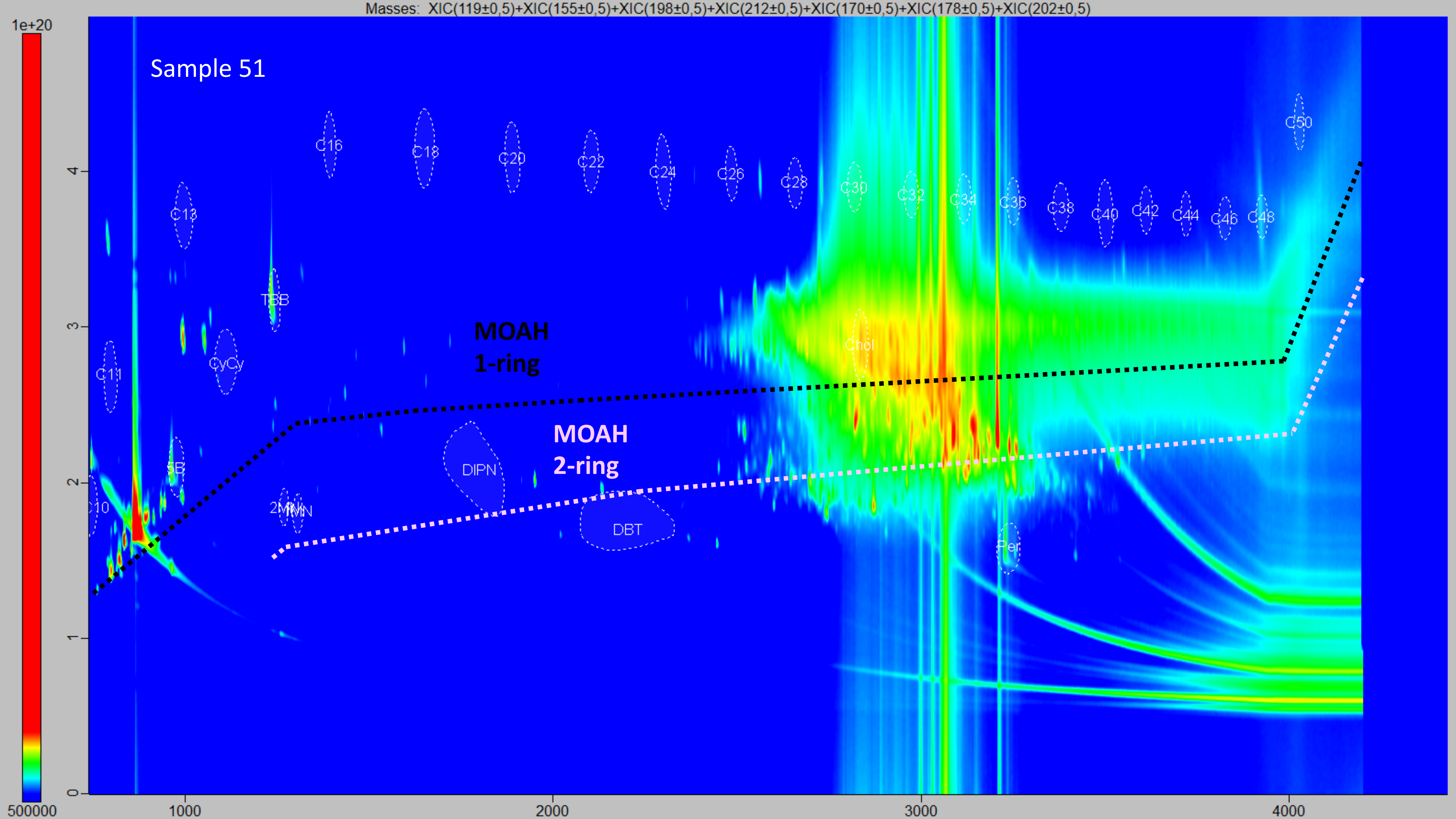
Masses: XIC(119±0,5)+XIC(155±0,5)+XIC(198±0,5)+XIC(212±0,5)+XIC(170±0,5)+XIC(178±0,5)+XIC(202±0,5)
1e+20
500000
Sample 51
MOAH
1-ring
MOAH
2-ring
C10
C11
C13
C16
C18
C20
C22
C24
C26
C28
C30
C32
C34
C36
C38
C40
C42
C44
C46
C48
C50
TBB
CyCy
5B
DIPN
DBT
Chol
Per
0
1
2
3
4
1000
2000
3000
4000
