# Supplementary figures and images for: The Mineral Oil Hydrocarbon Paradox in Olive Pomace Oils
Source: Foods. 2023 Jan 17;12(3):434. doi: 10.3390/foods12030434 (PMC9914016; doi:10.3390/foods12030434)

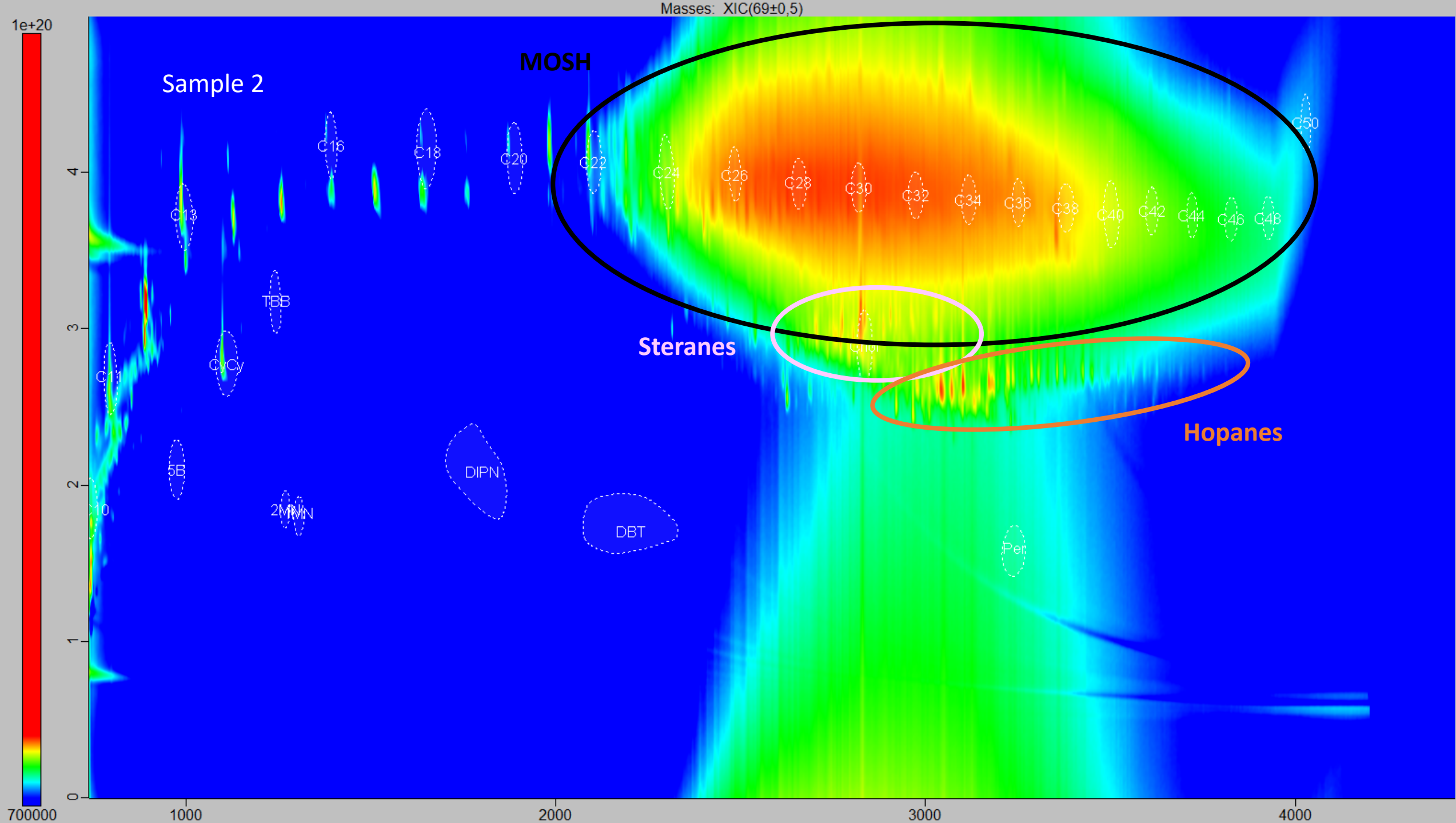

1e+20

700000

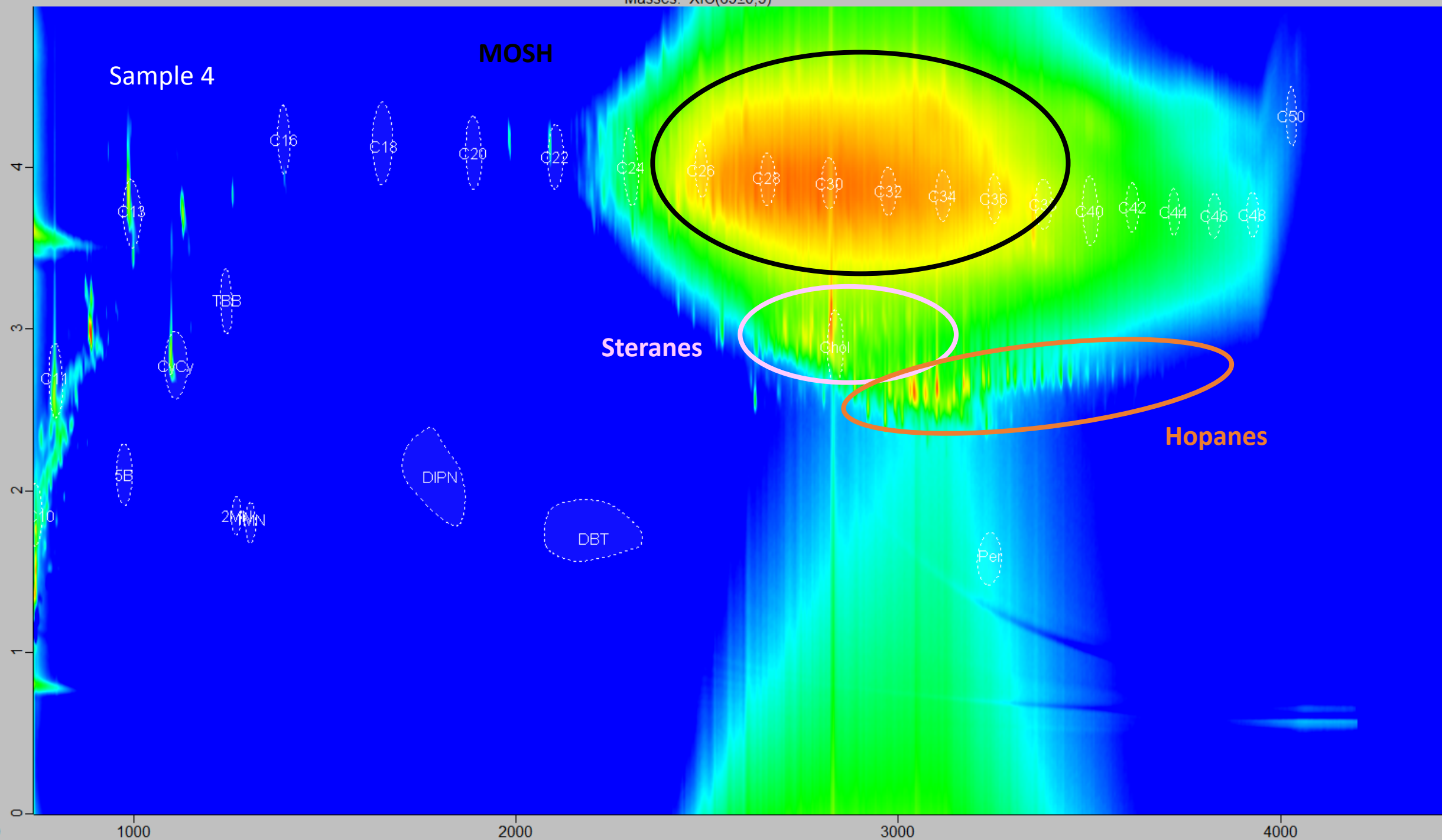

1e+20

700000

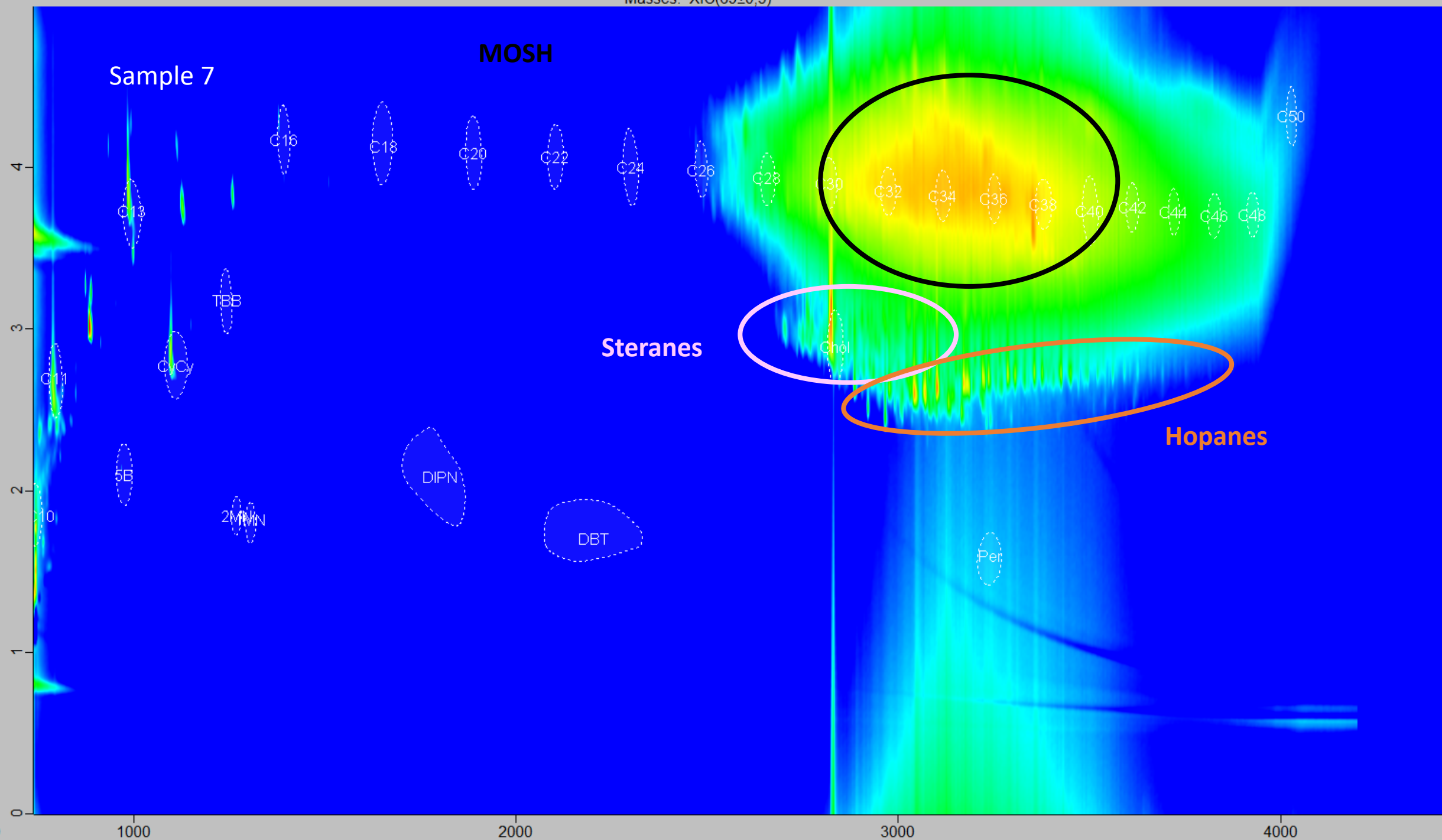

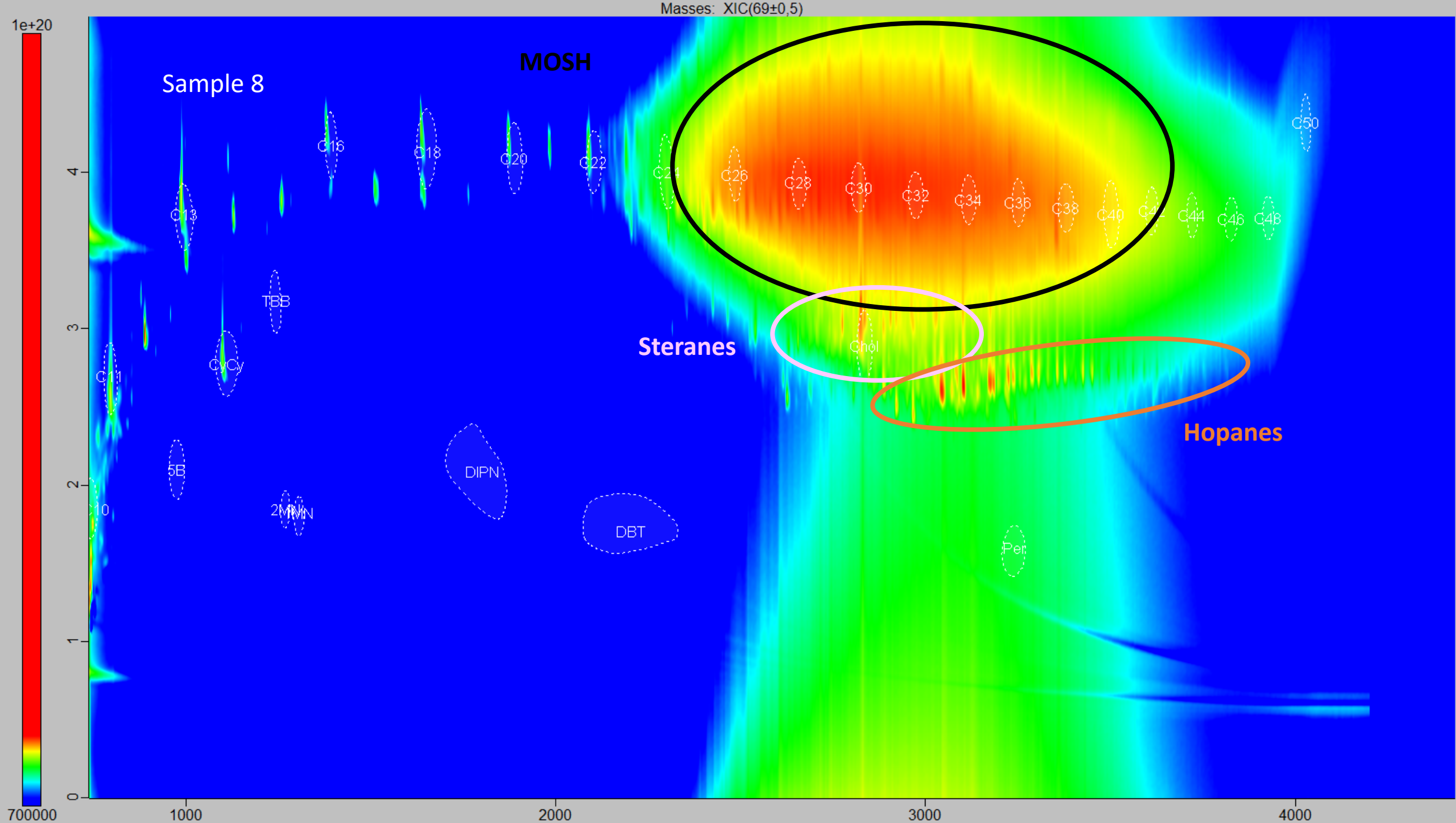

1e+20

700000

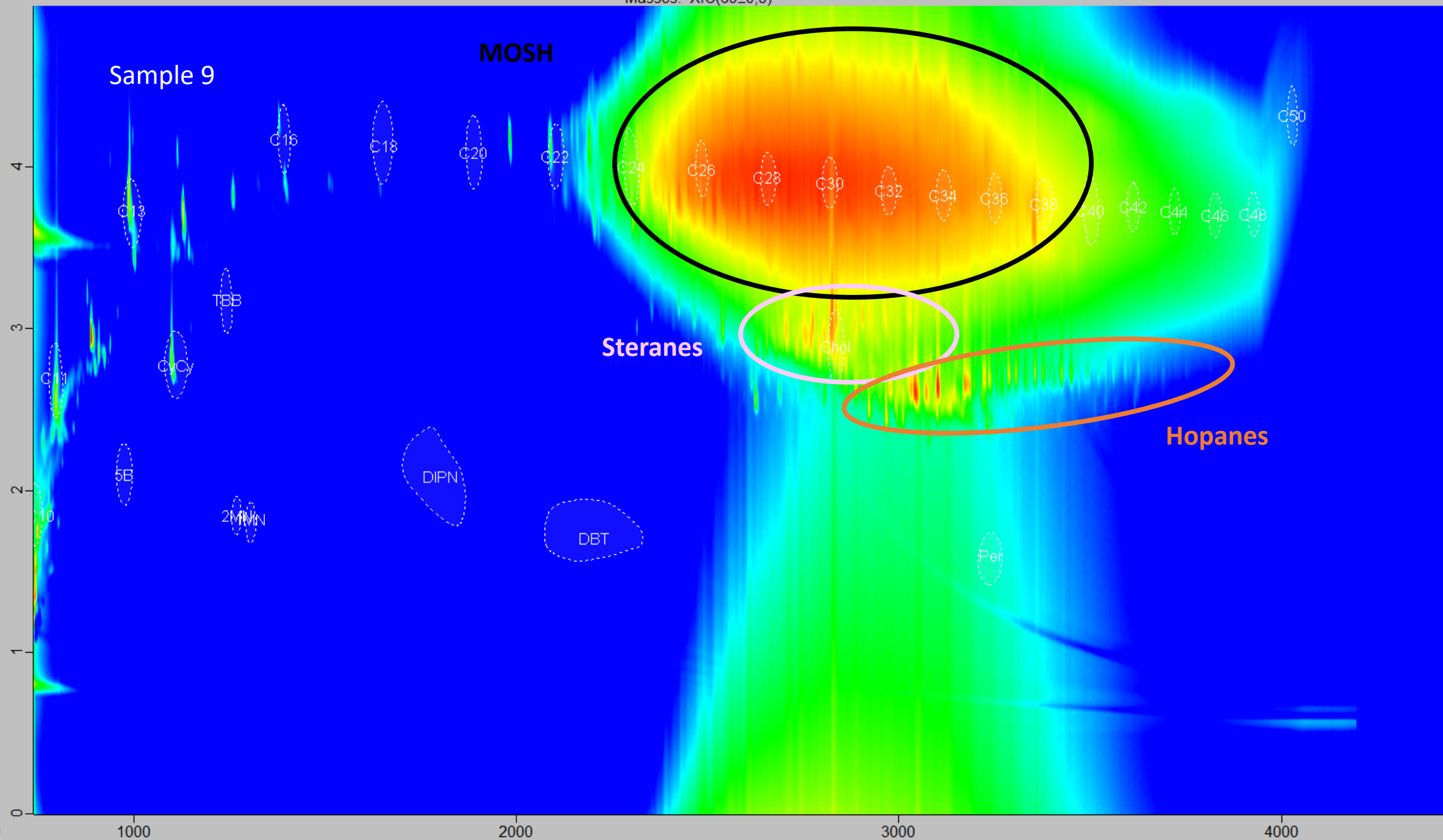

1e+20

700000

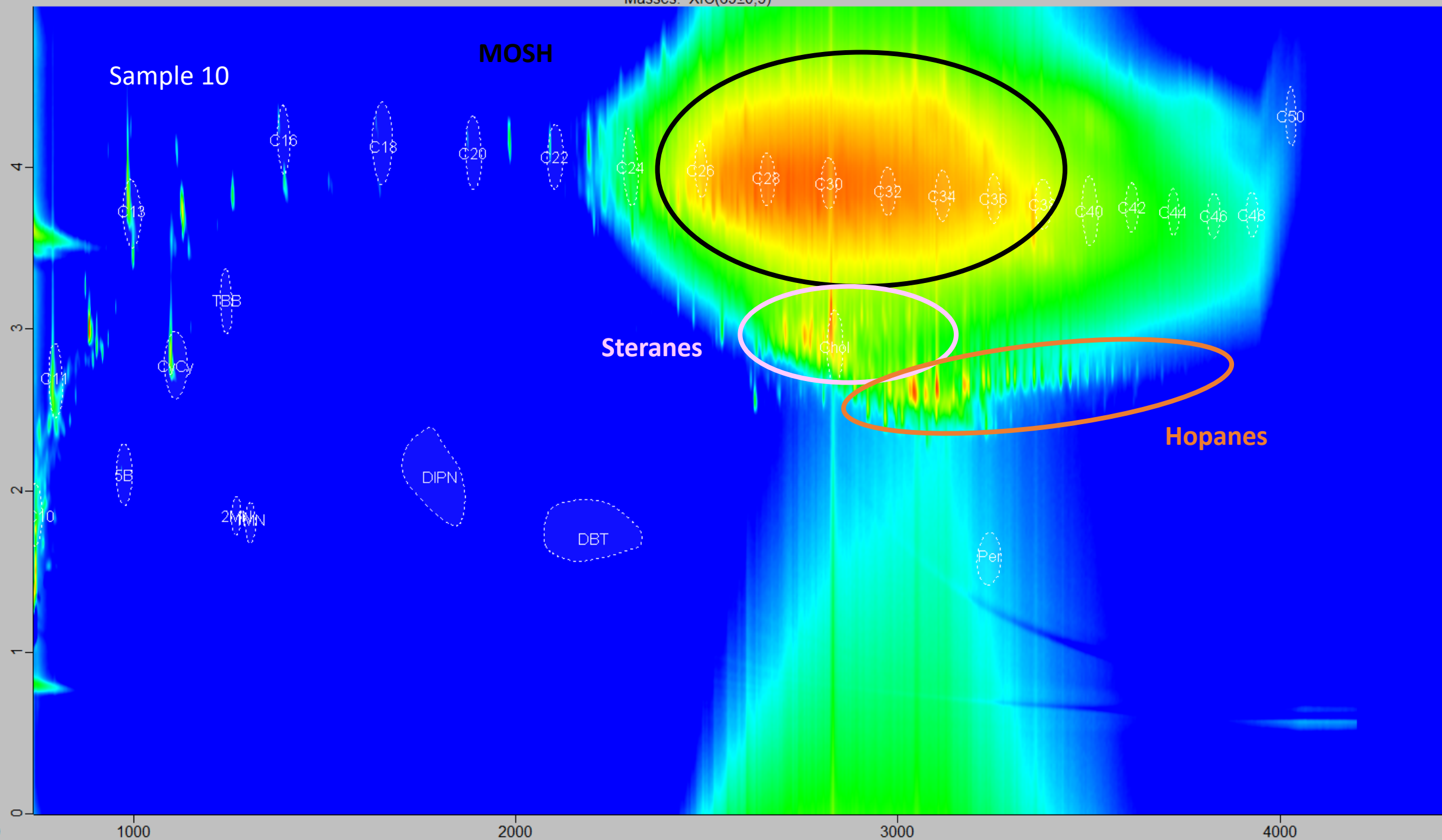

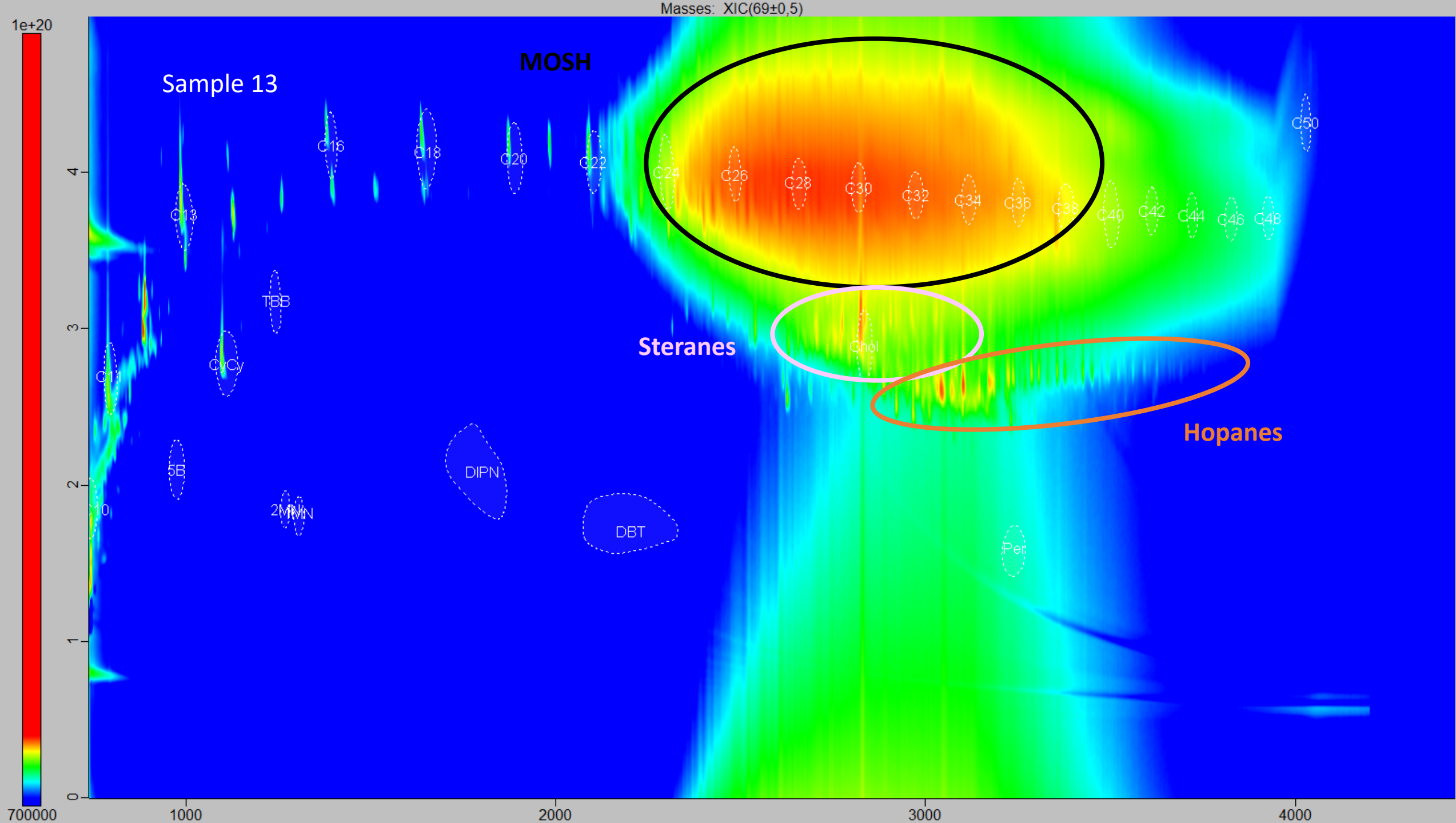

1e+20

700000

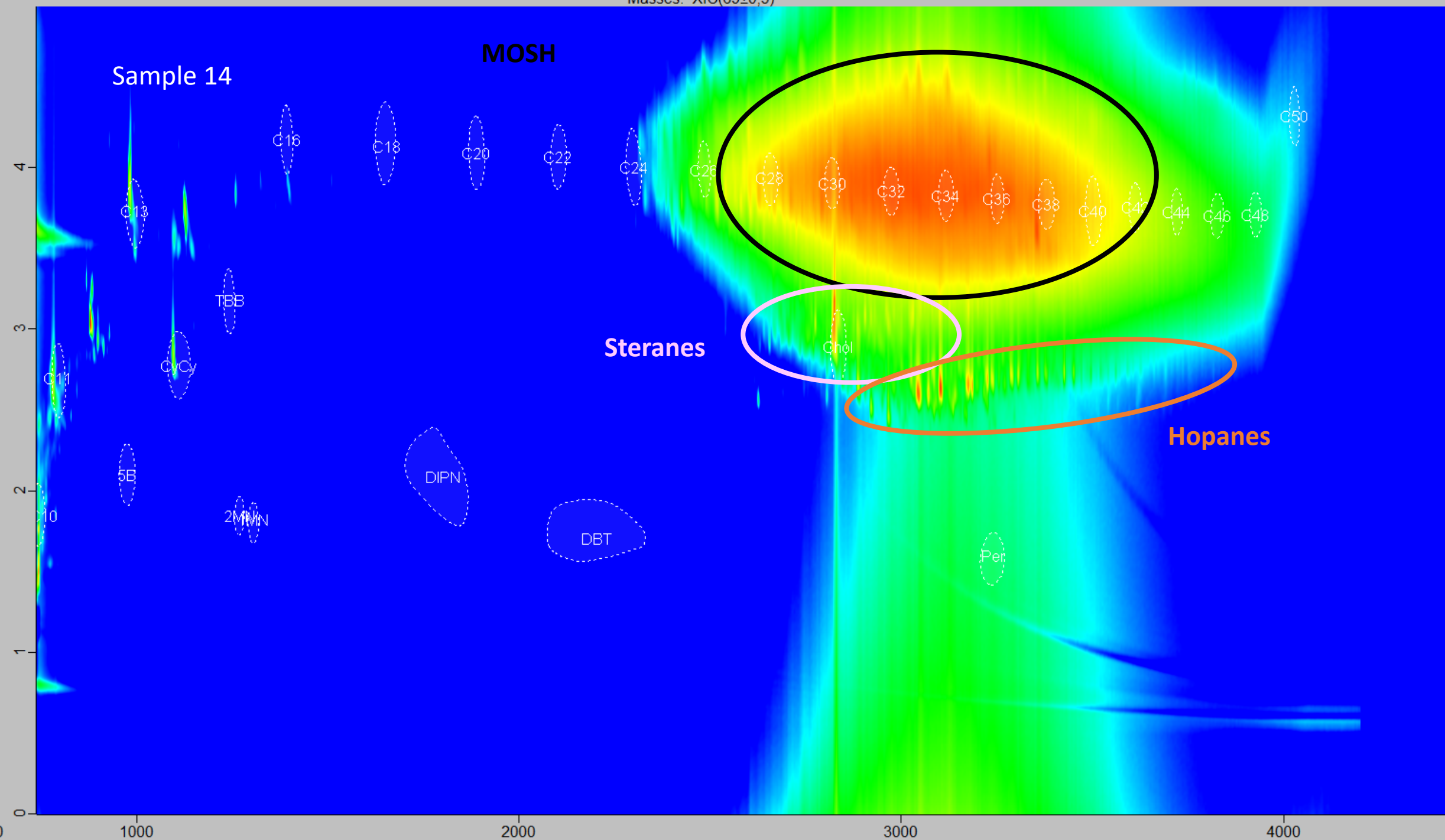

1e+20

700000

**MOSH****Sample 16****Steranes****Hopanes**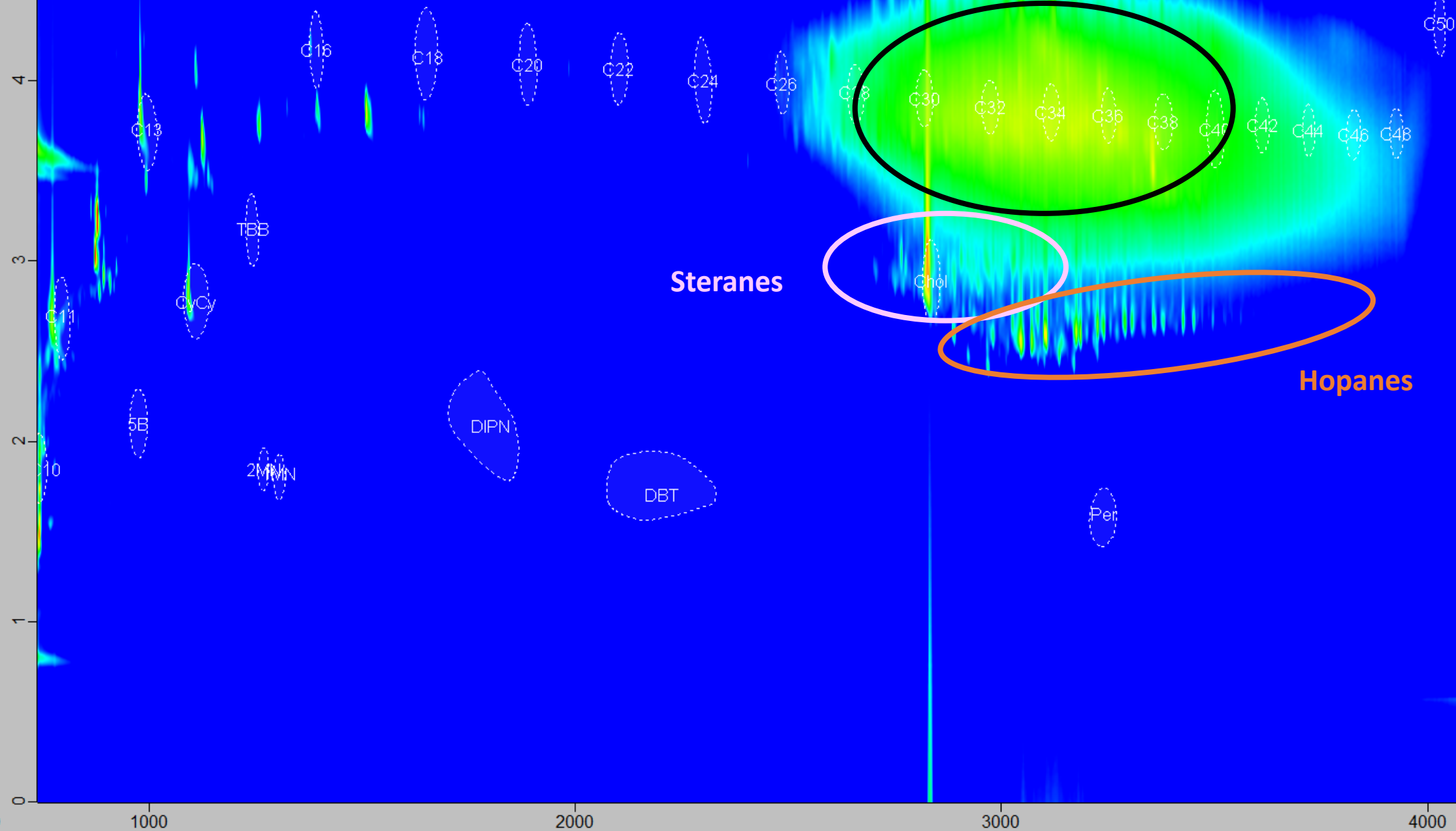

1e+20

700000

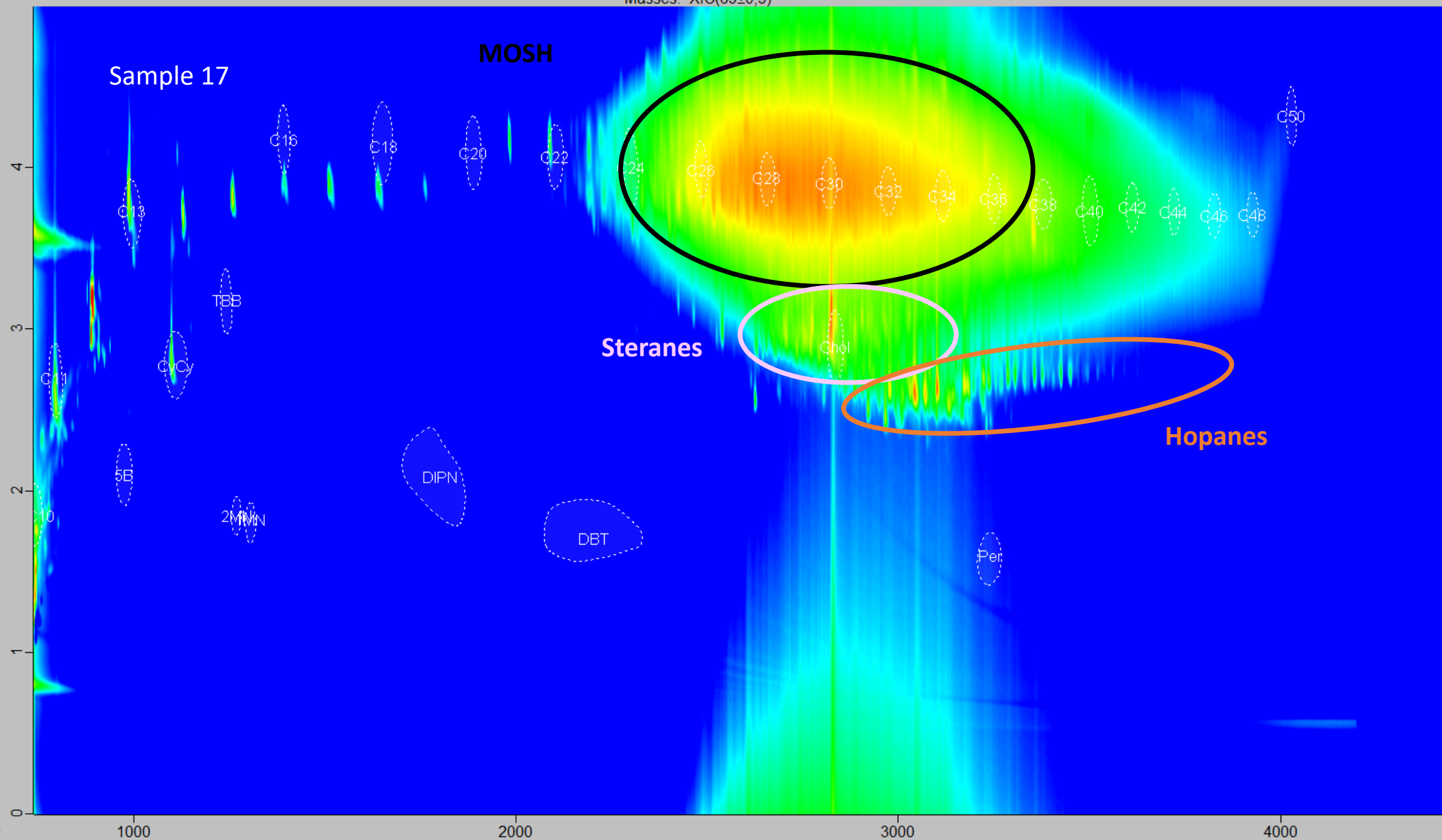

1e+20

700000

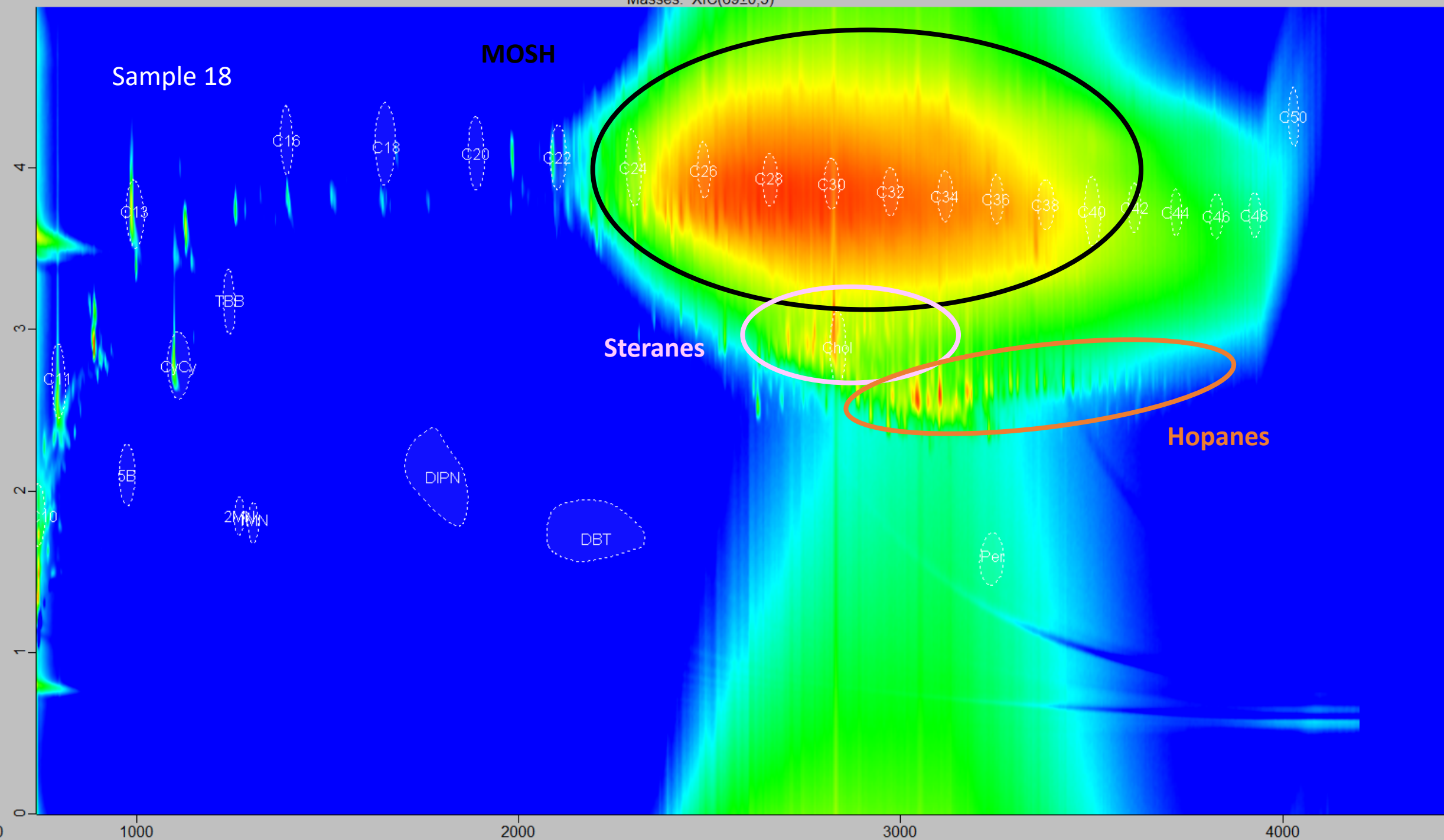

1e+20

700000

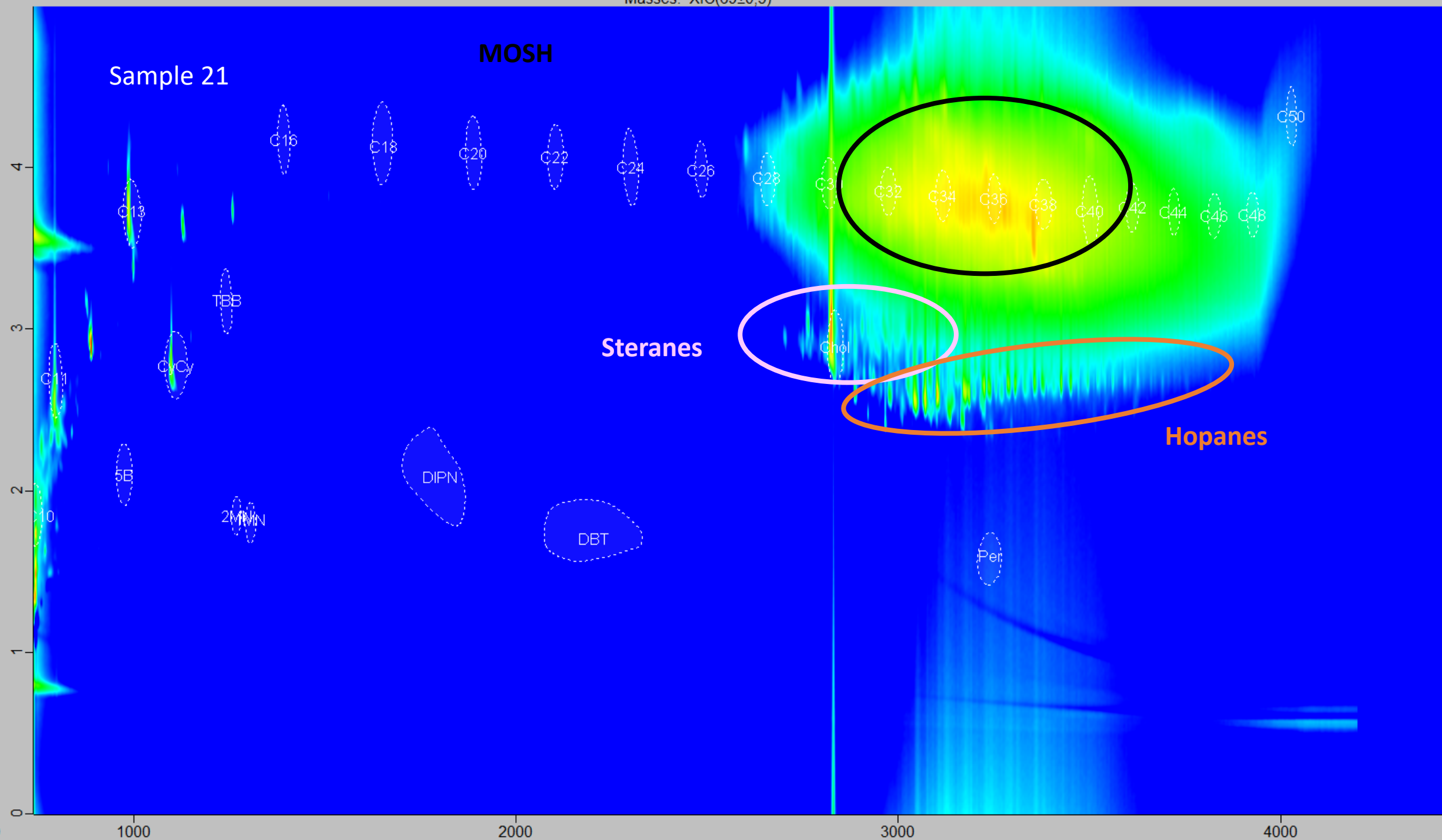

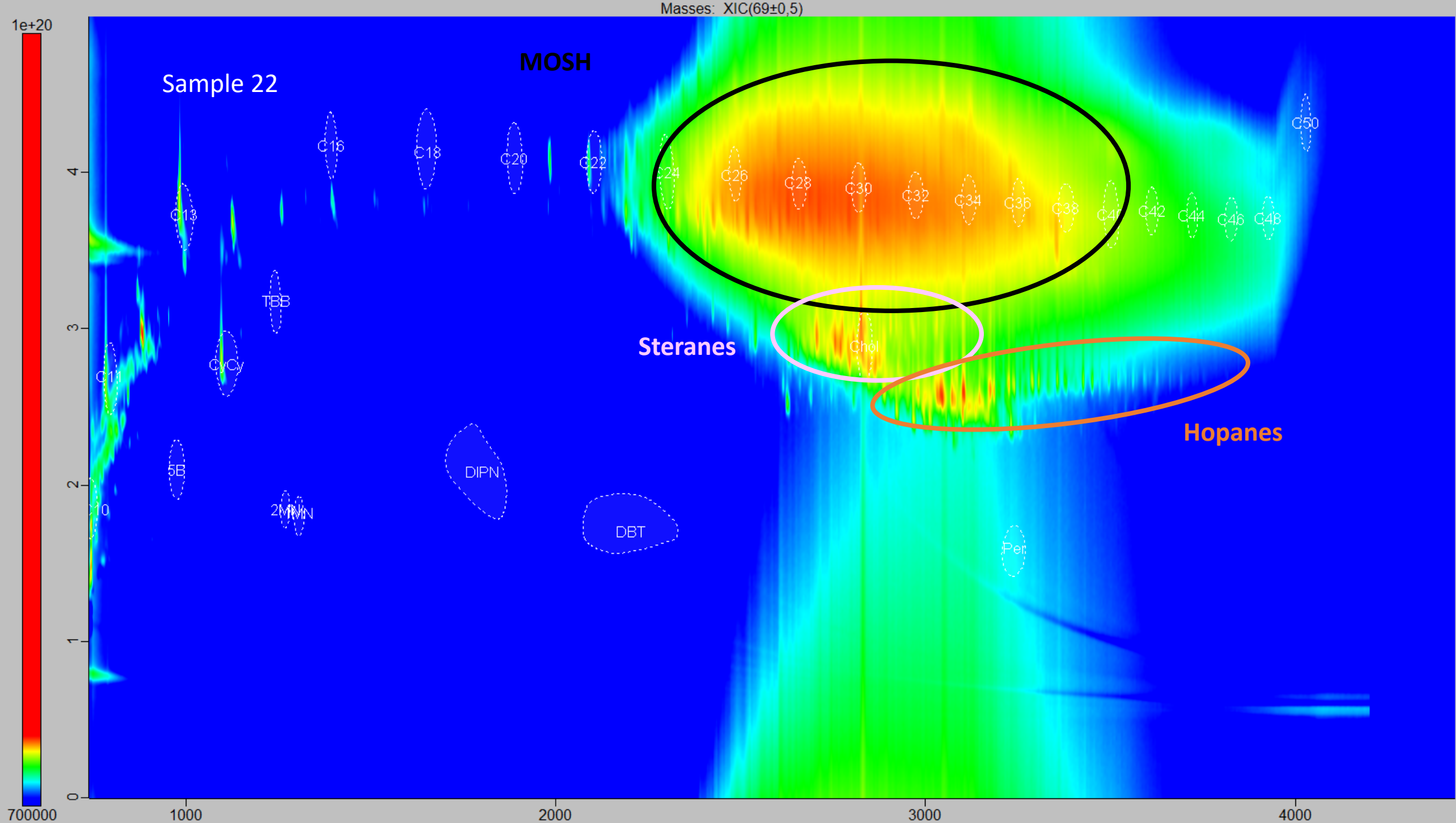

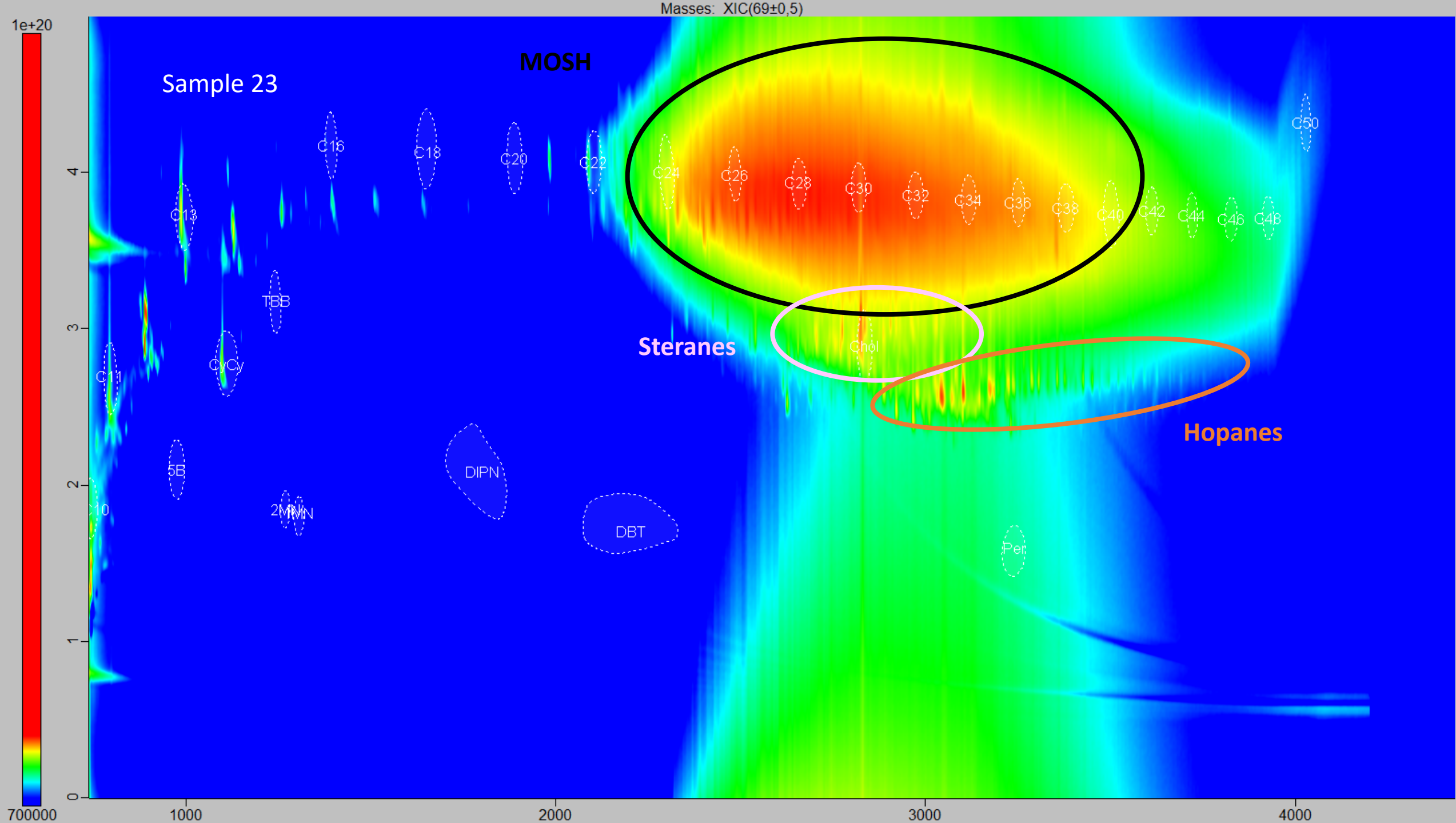

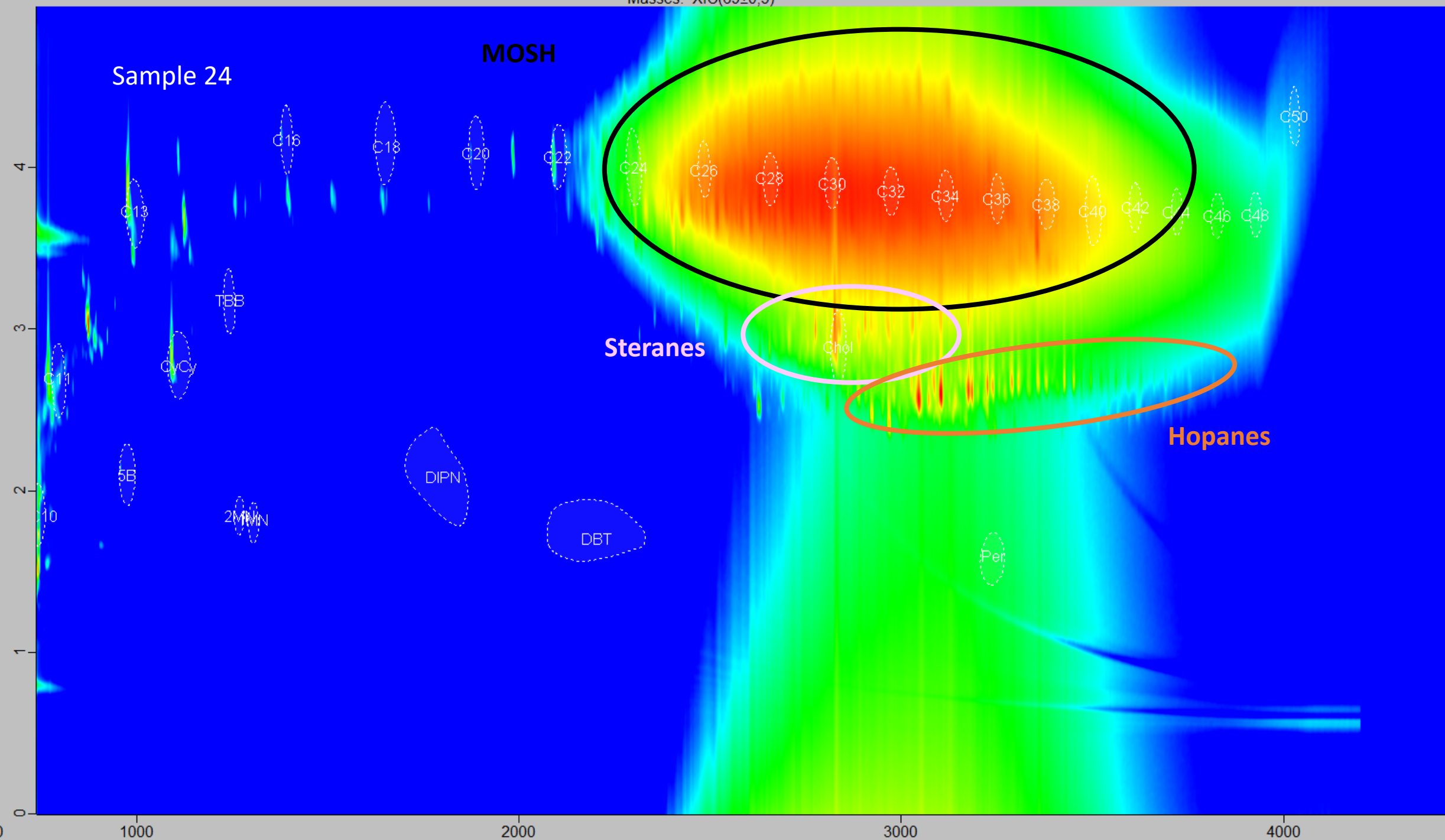

1e+20

700000

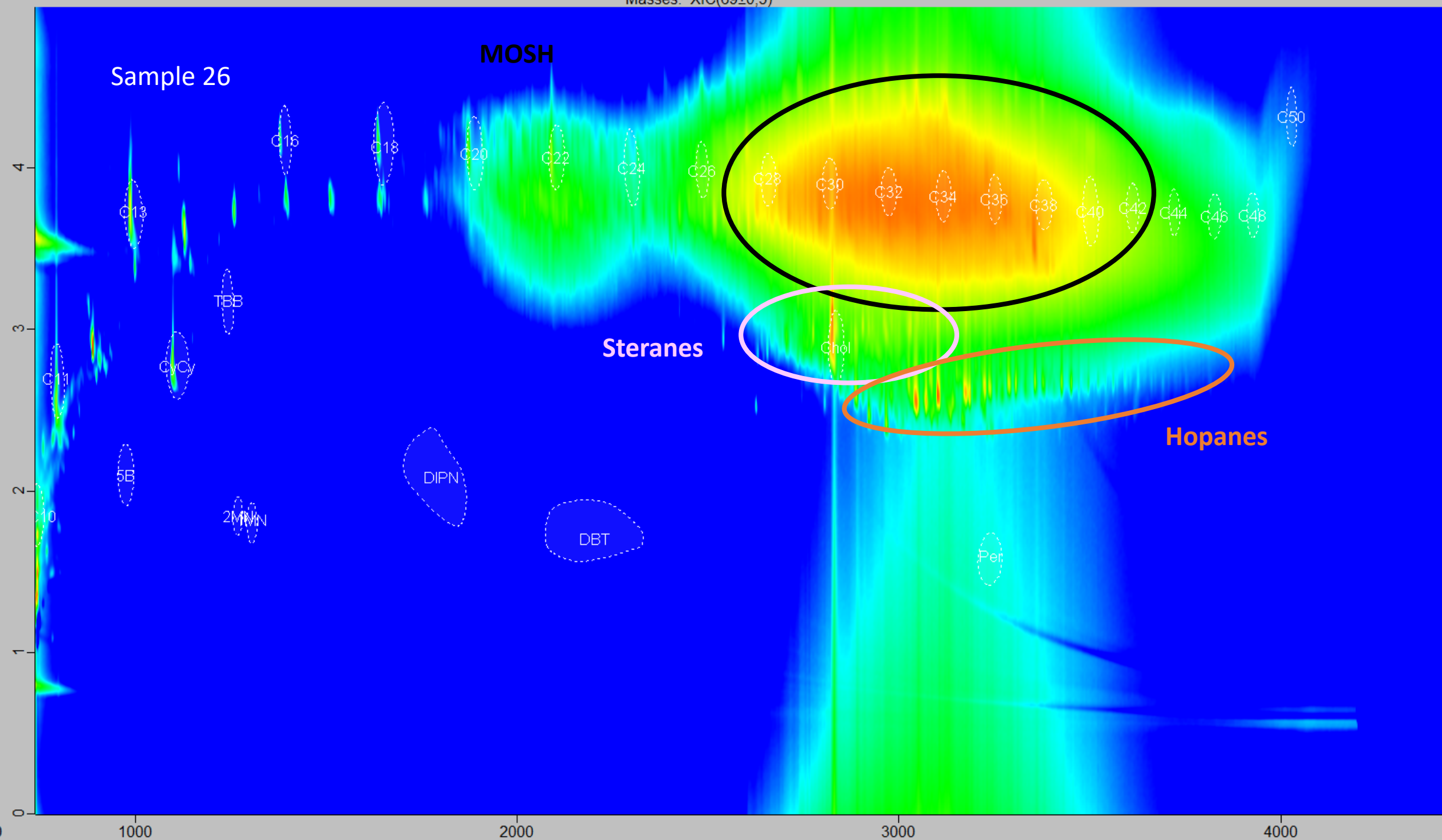

1e+20

700000

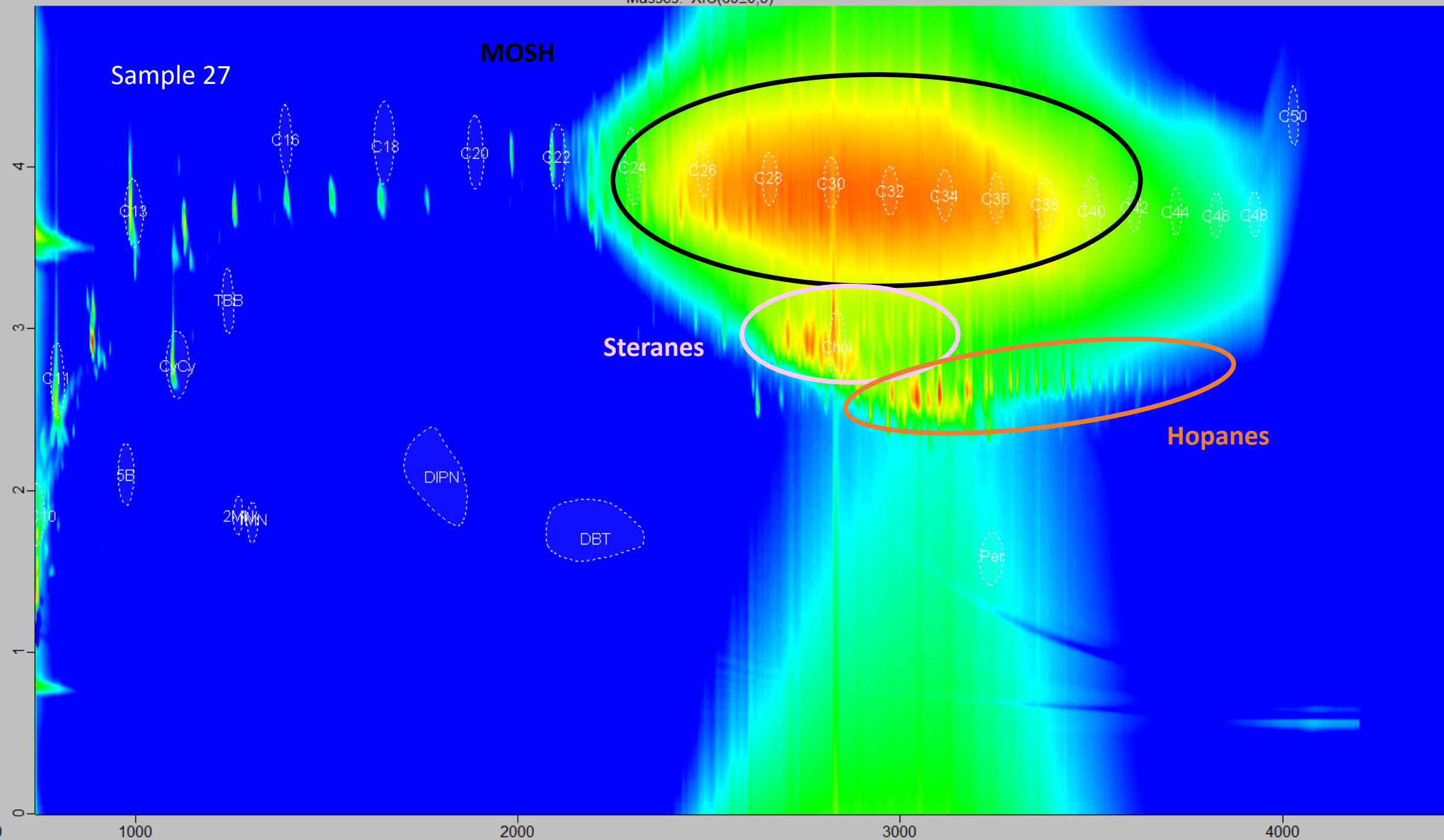

1e+20

700000

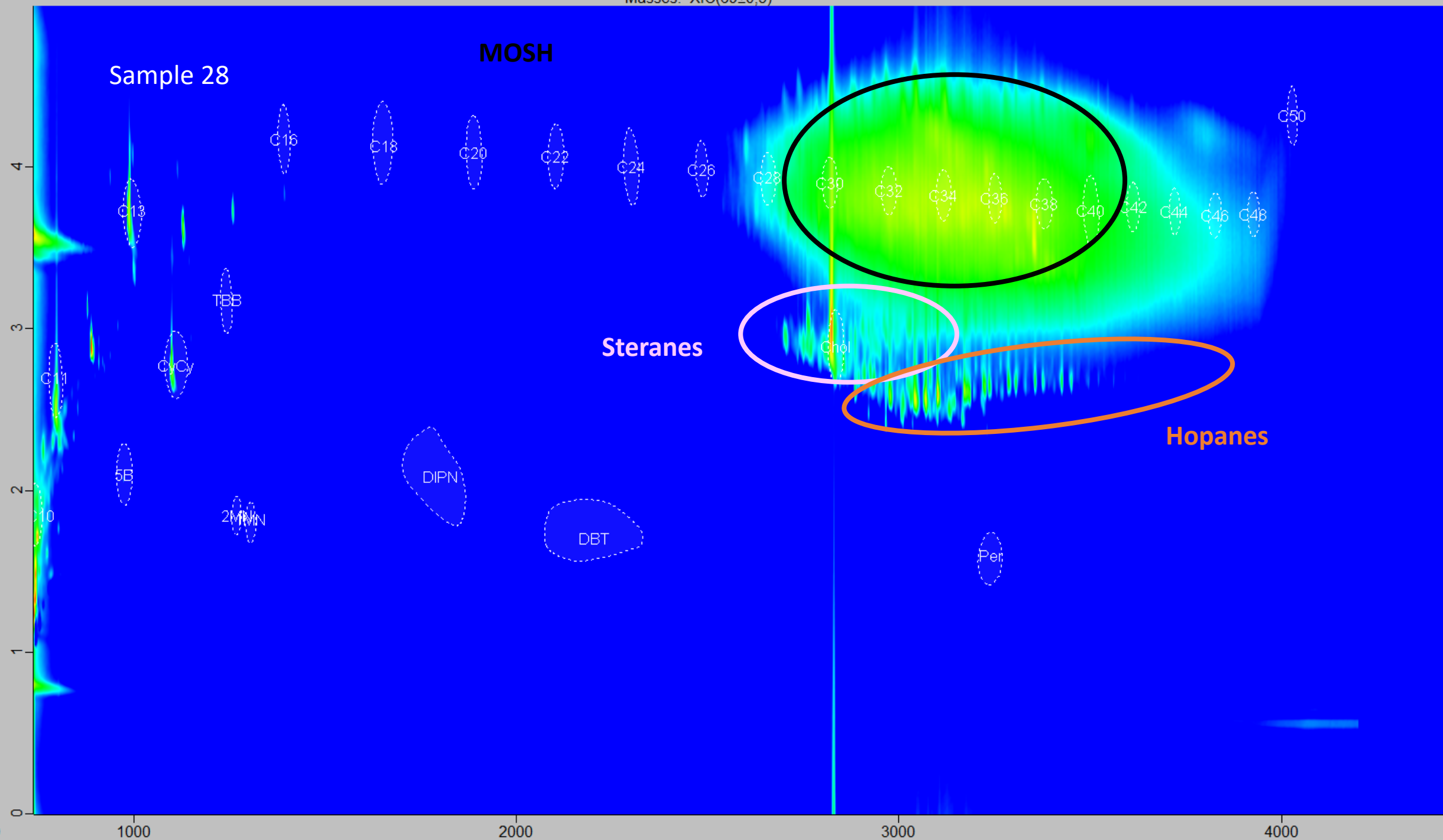

1e+20

700000

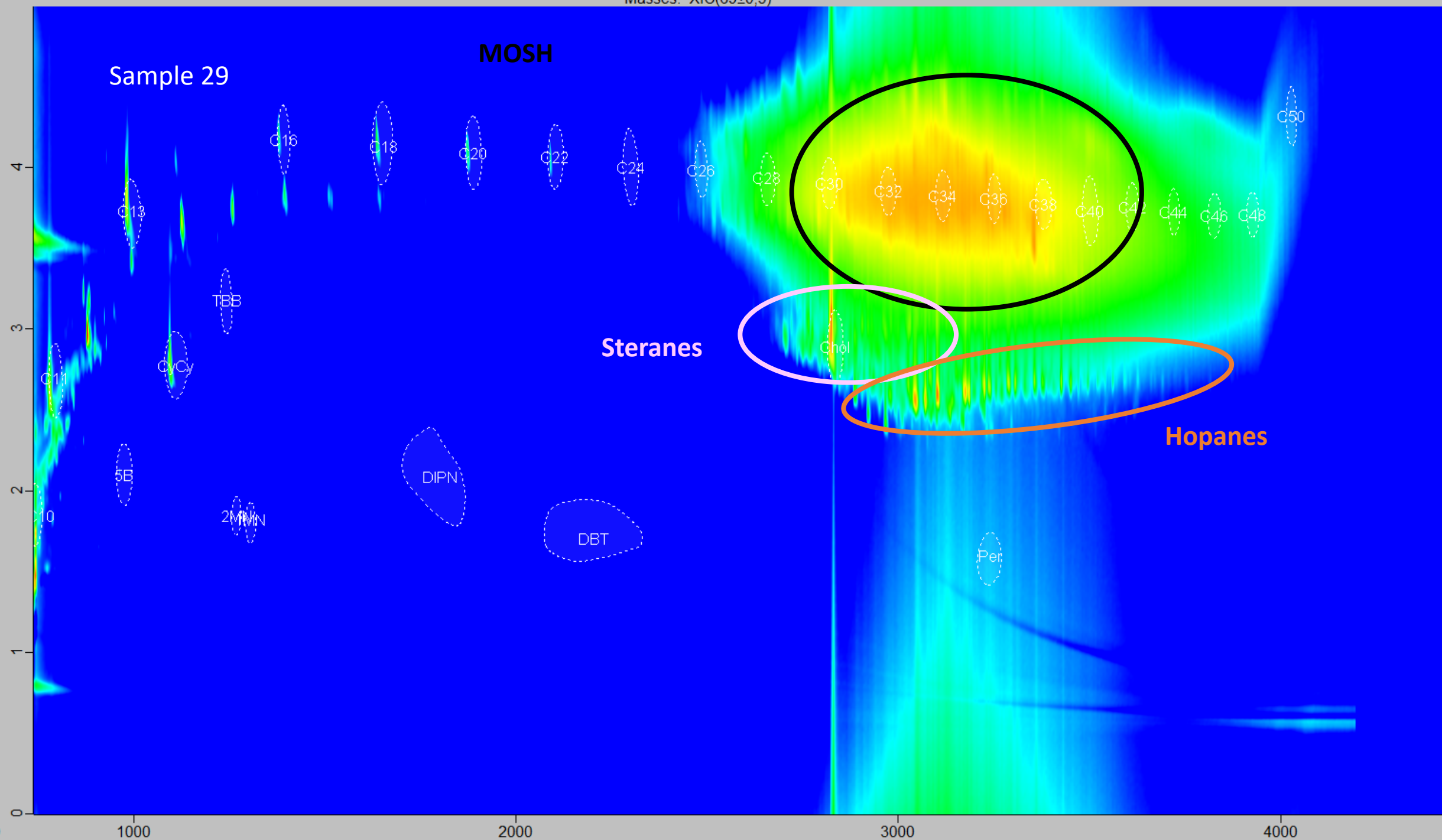

1e+20

700000

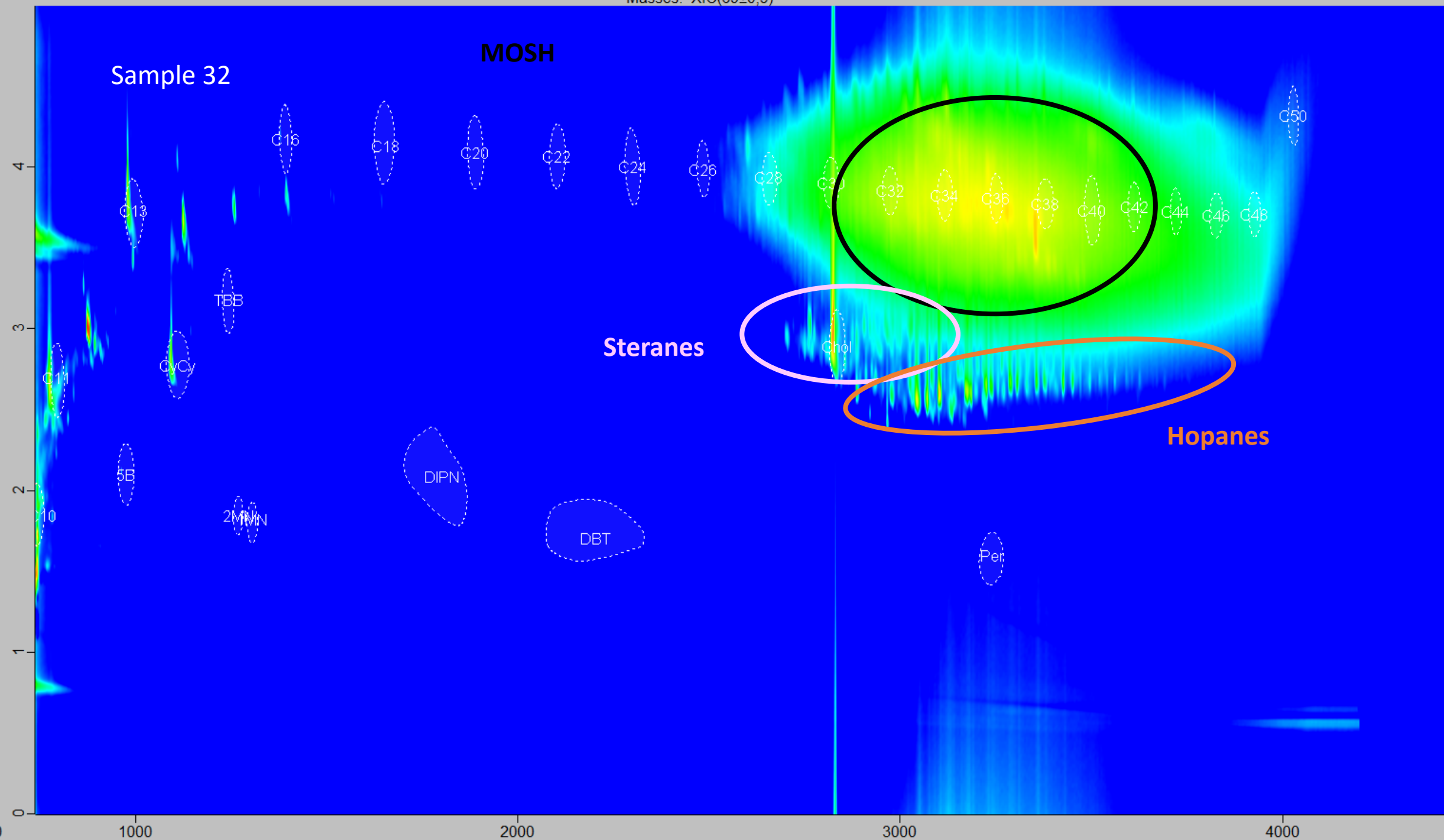

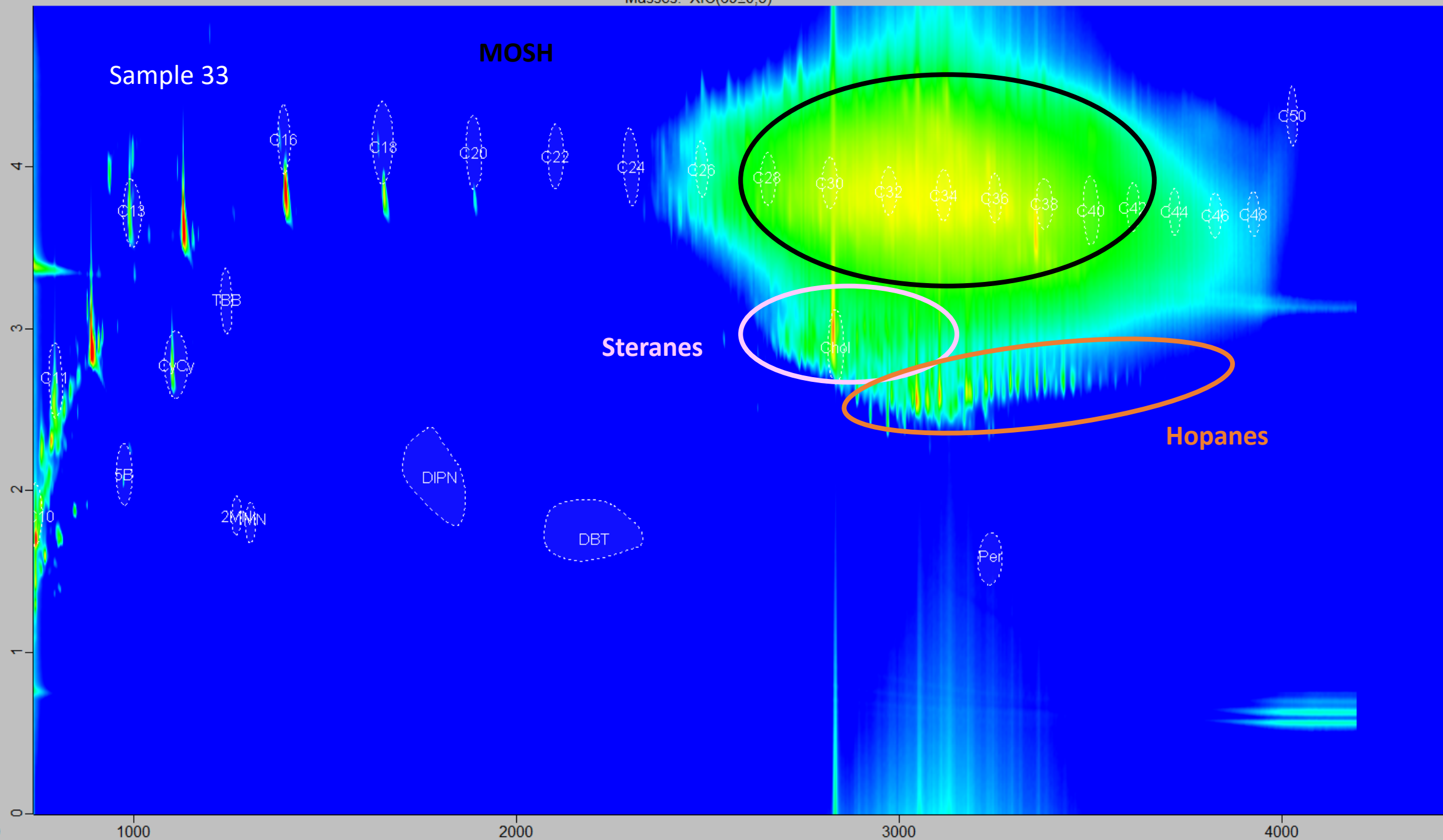

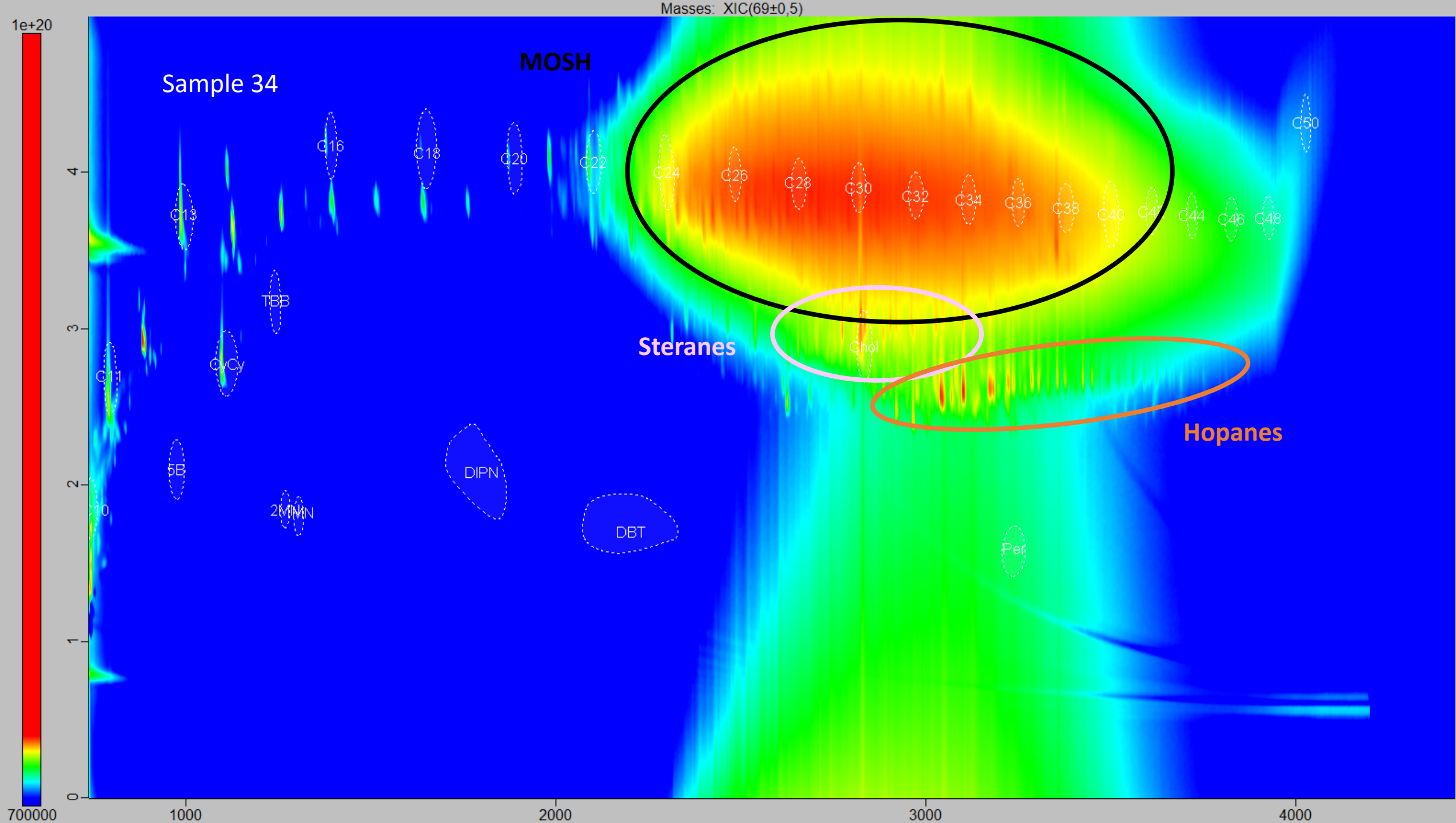

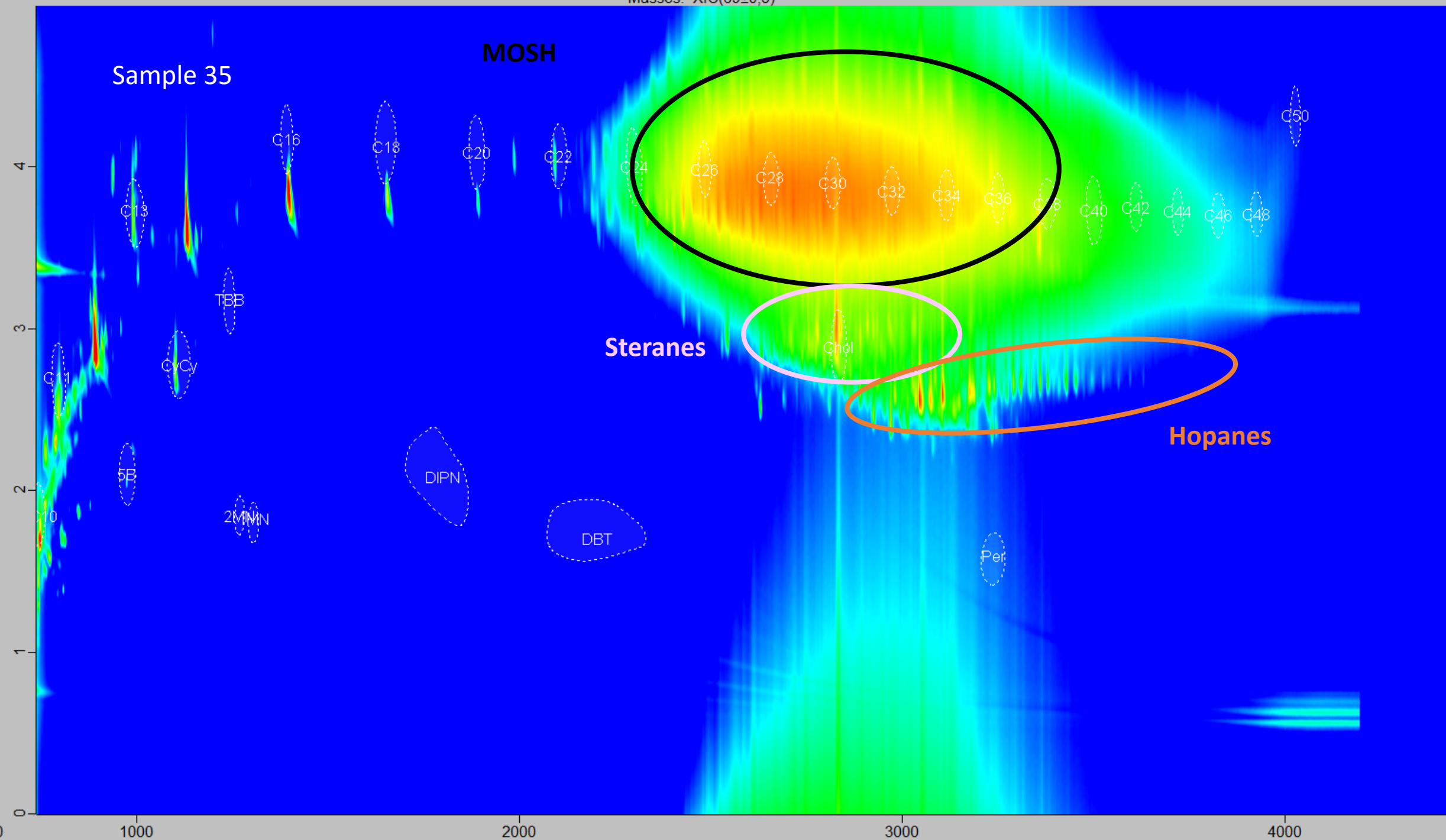

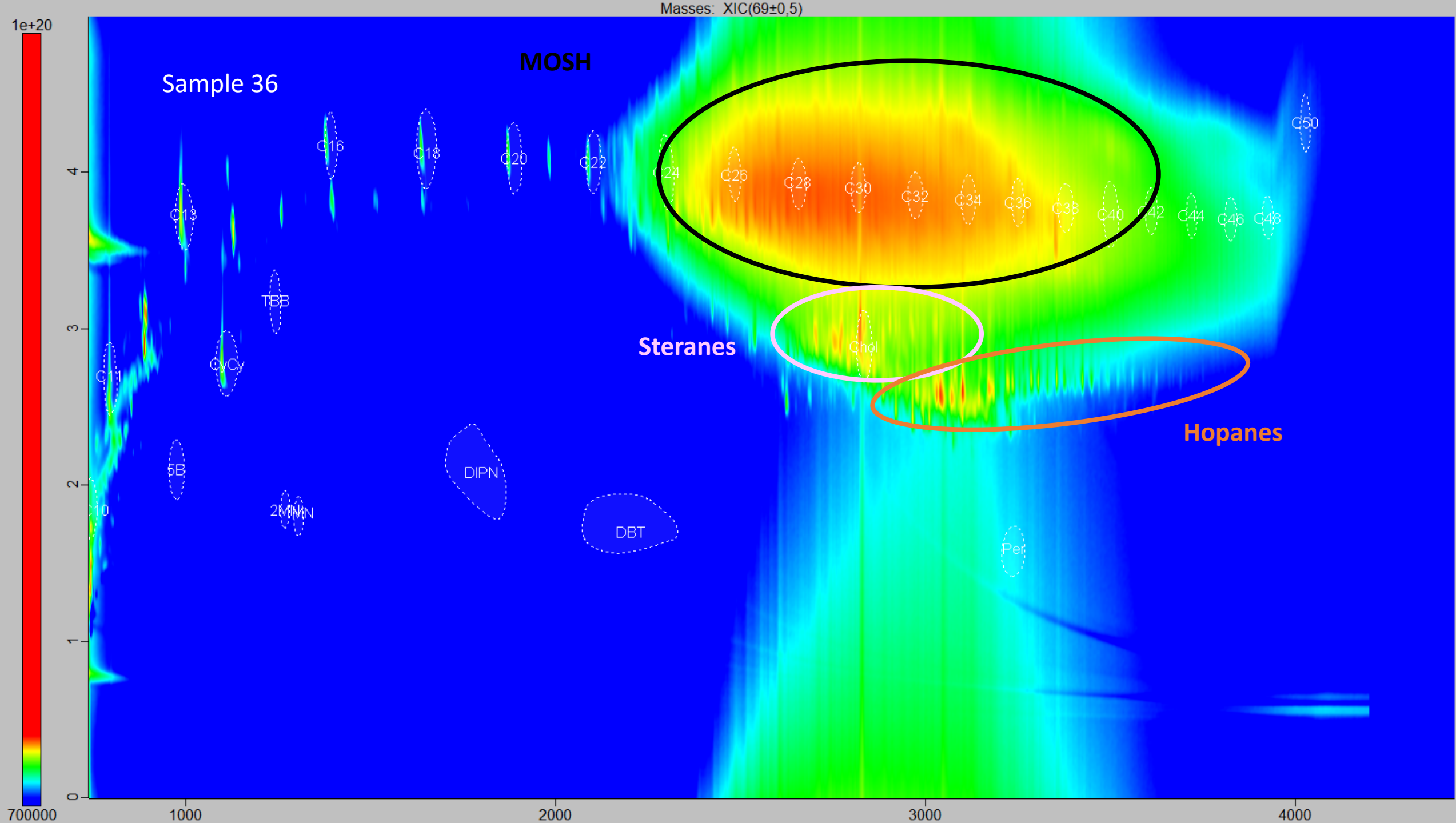

1e+20

700000

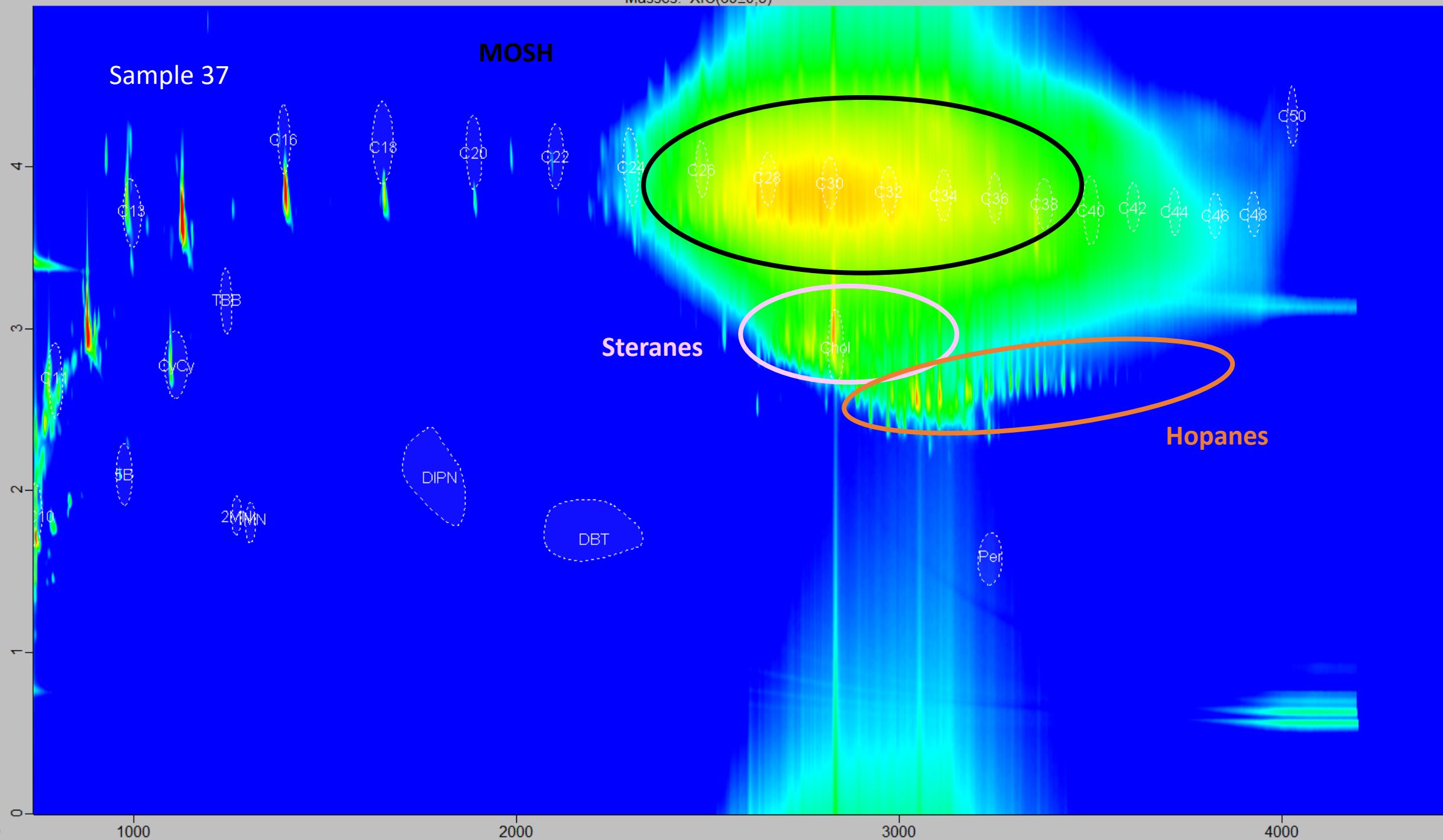

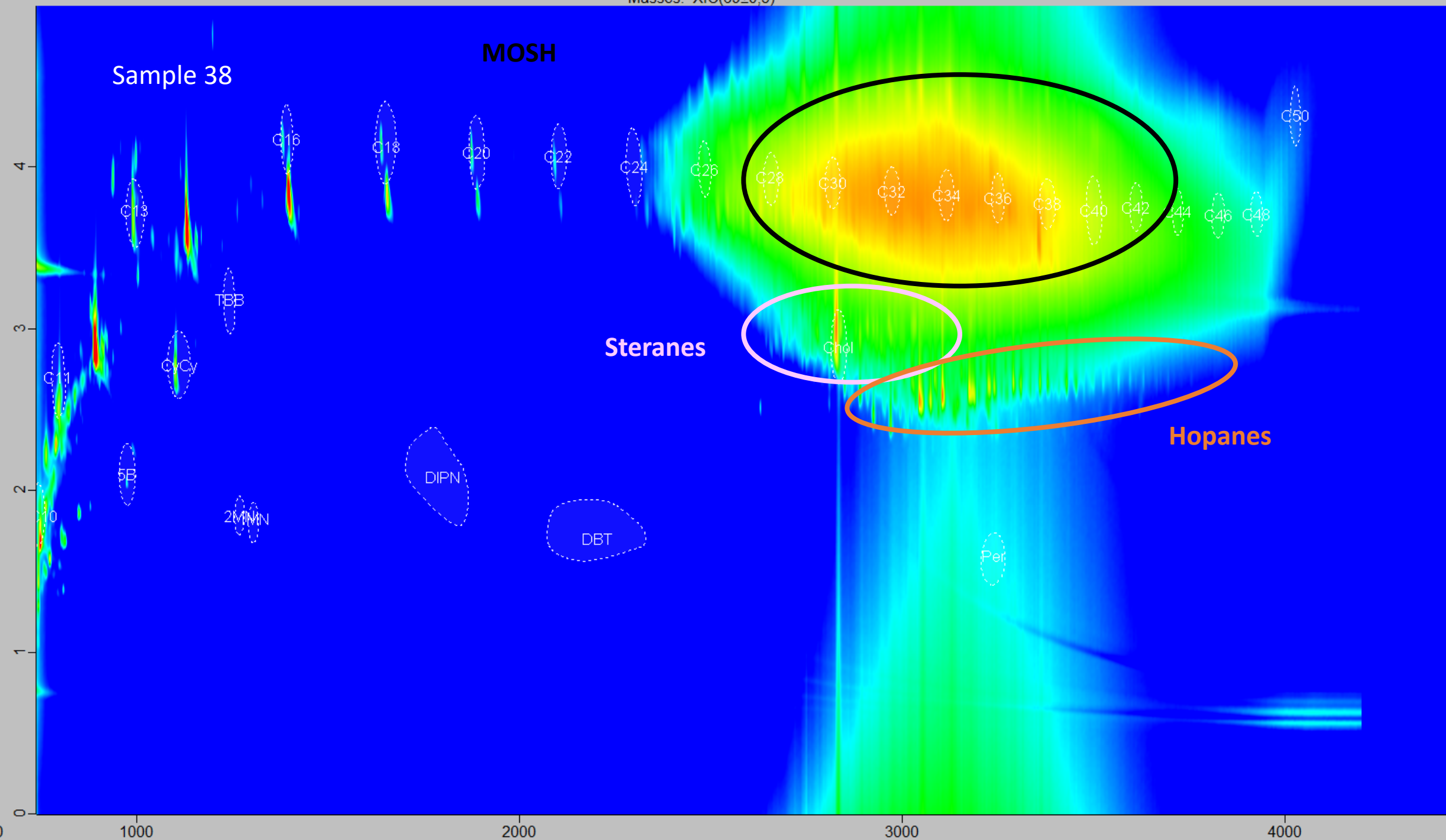

1e+20

700000

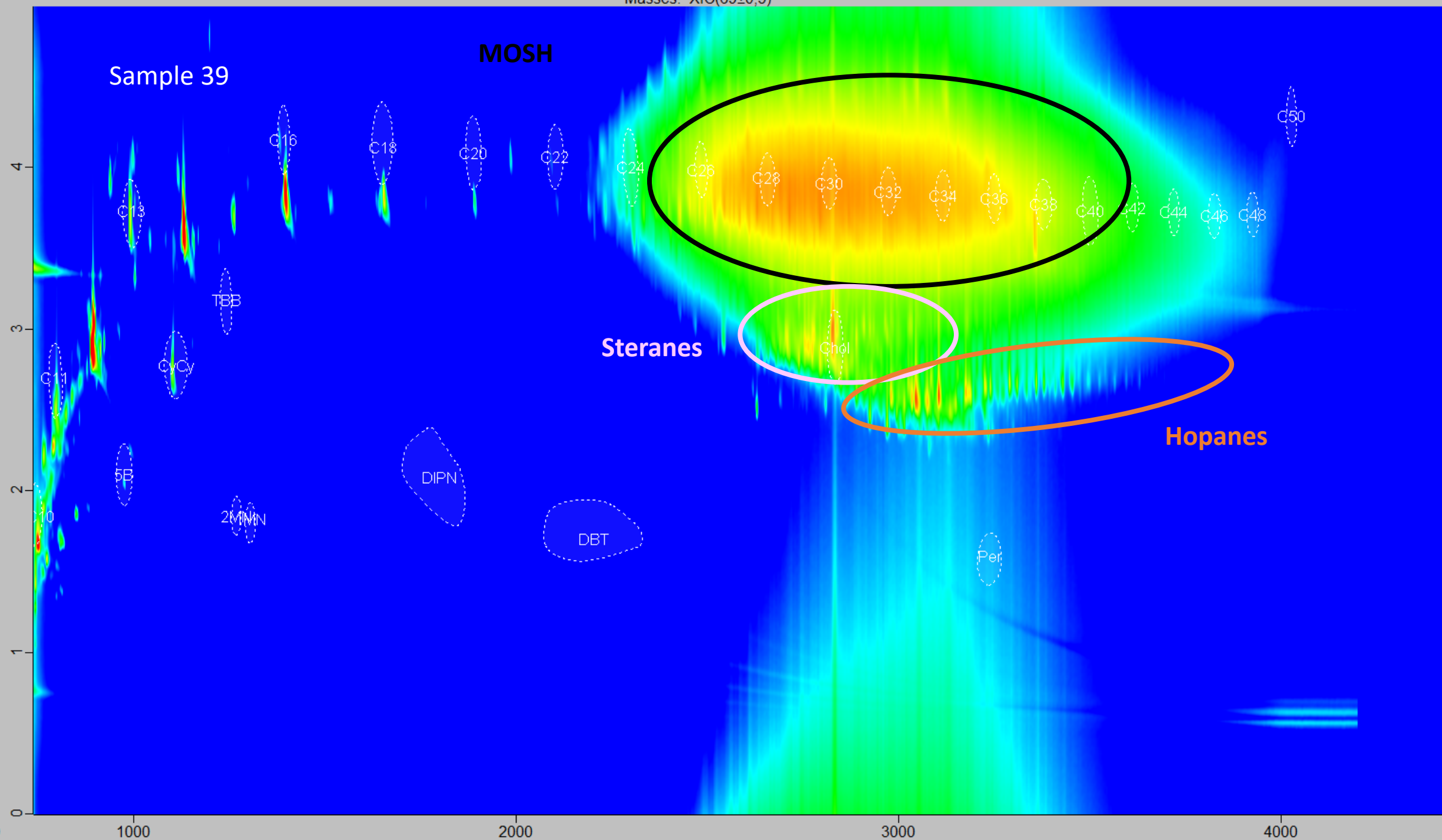

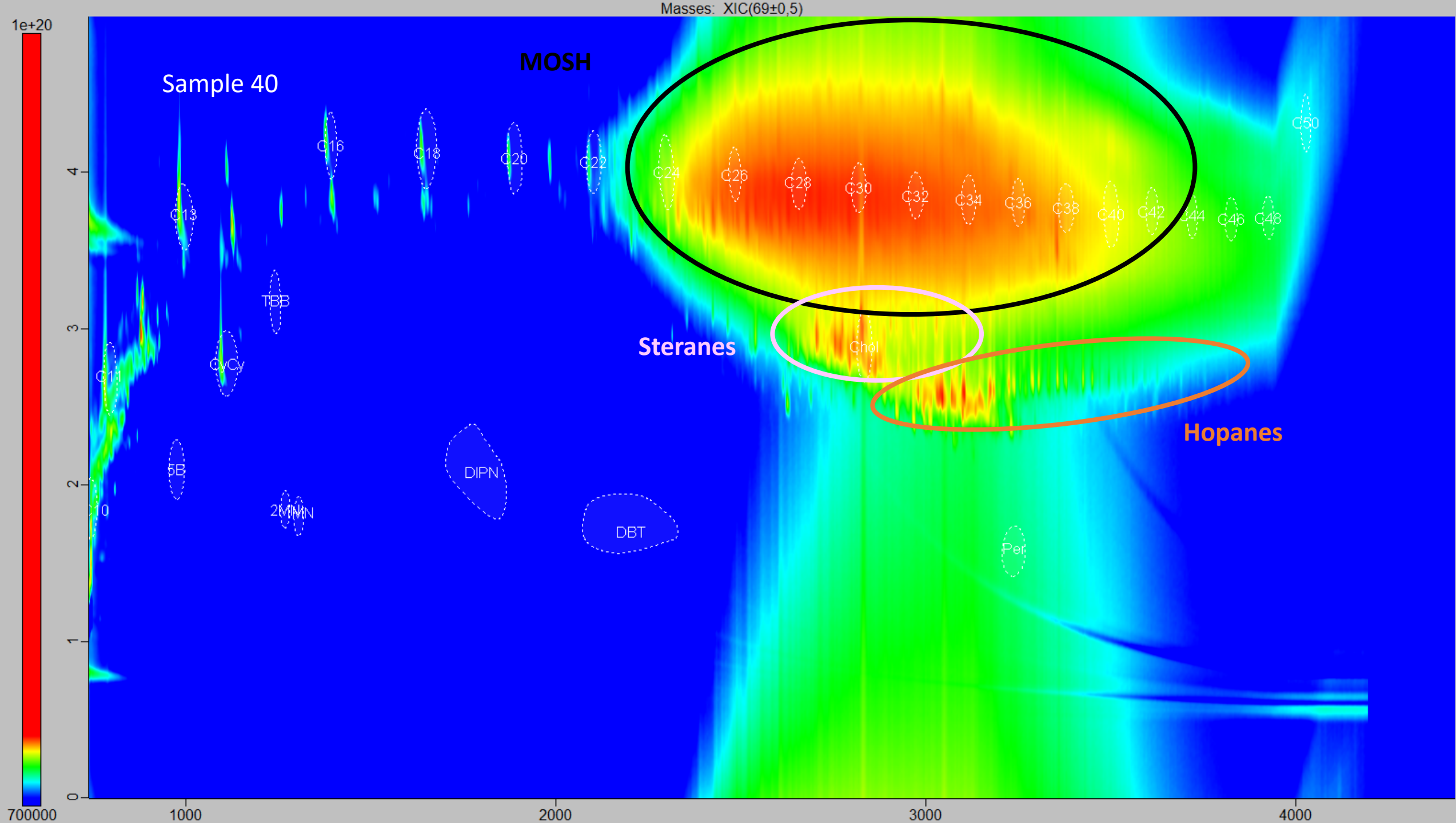

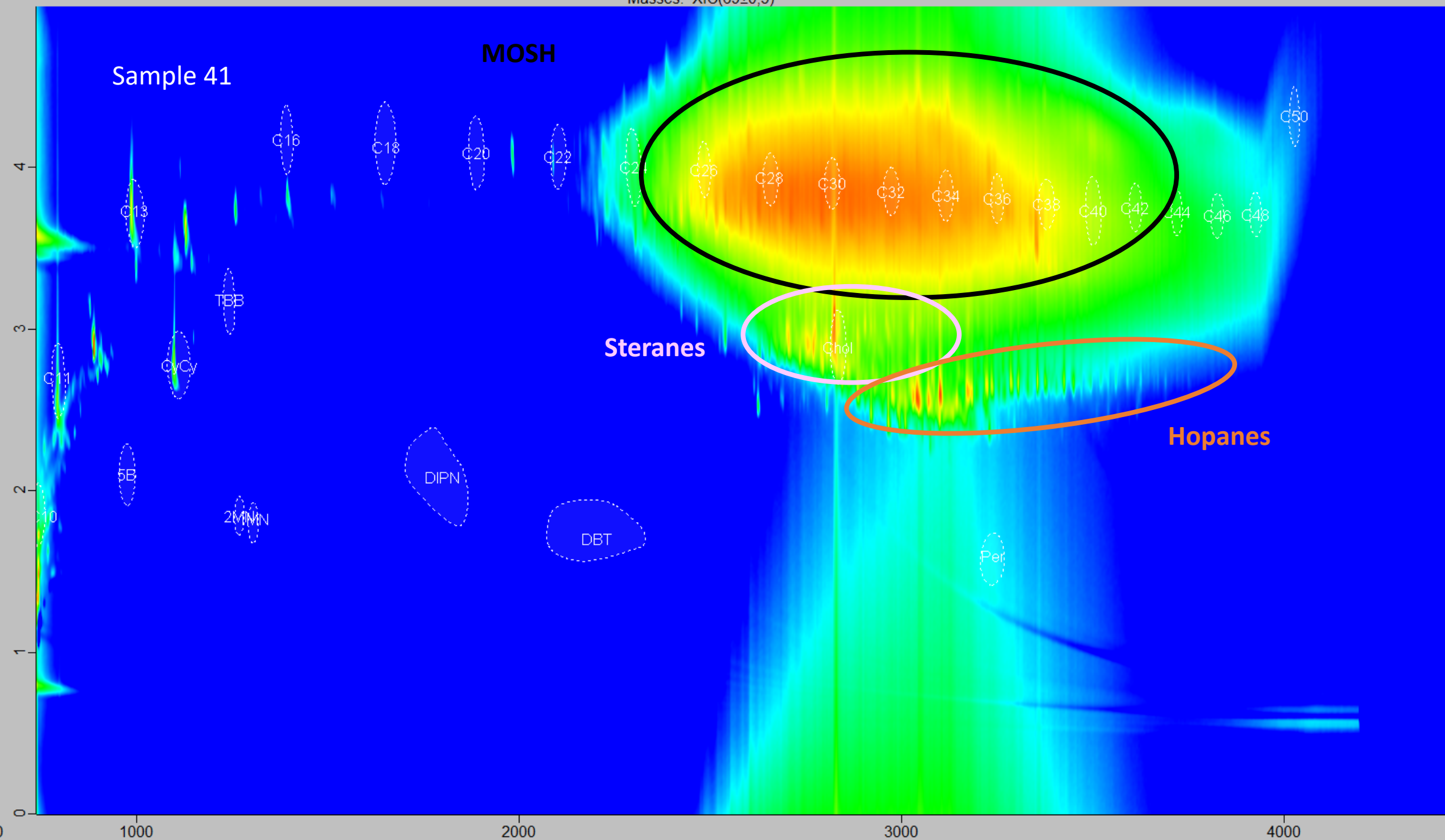

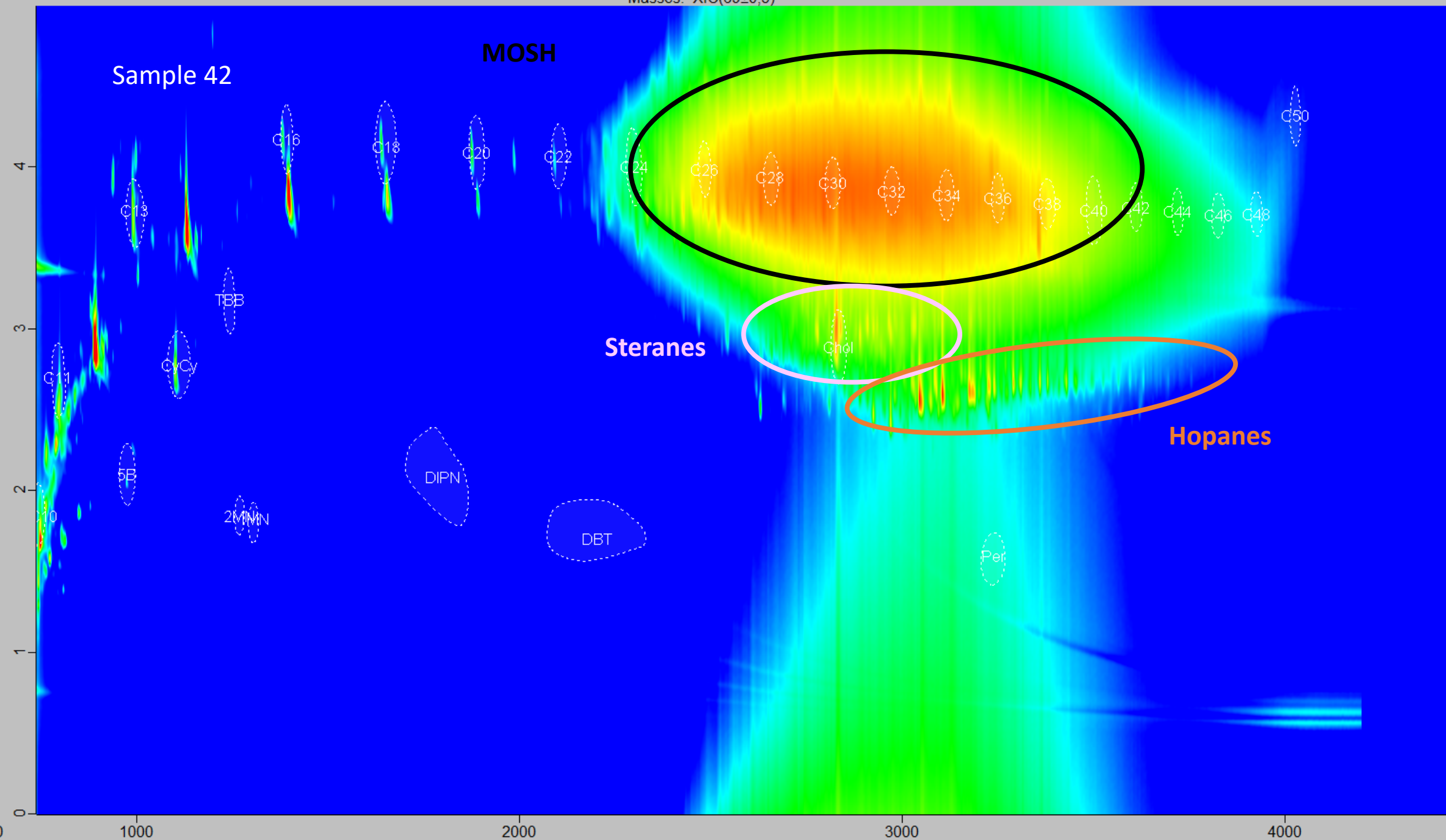

1e+20

700000

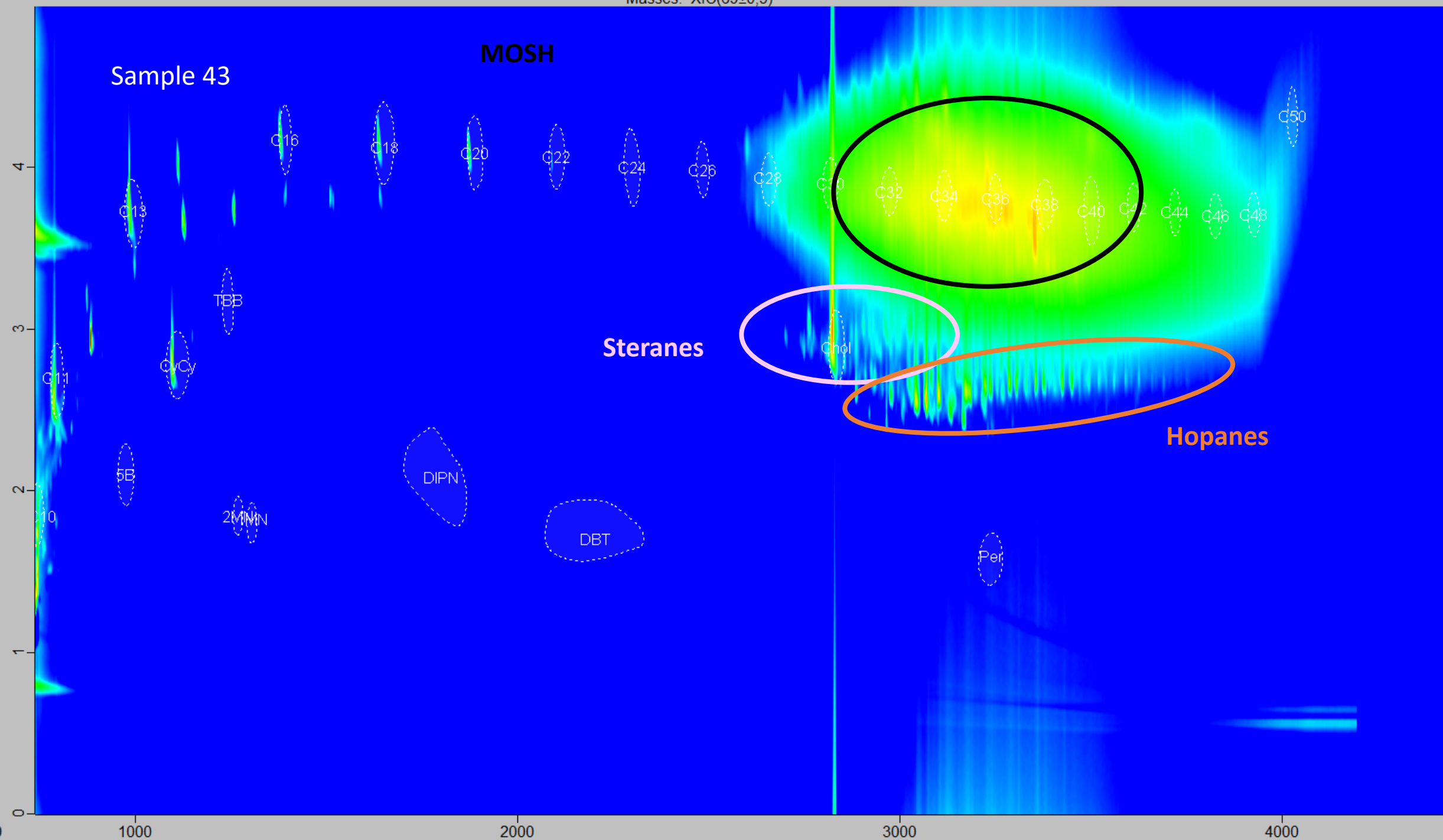

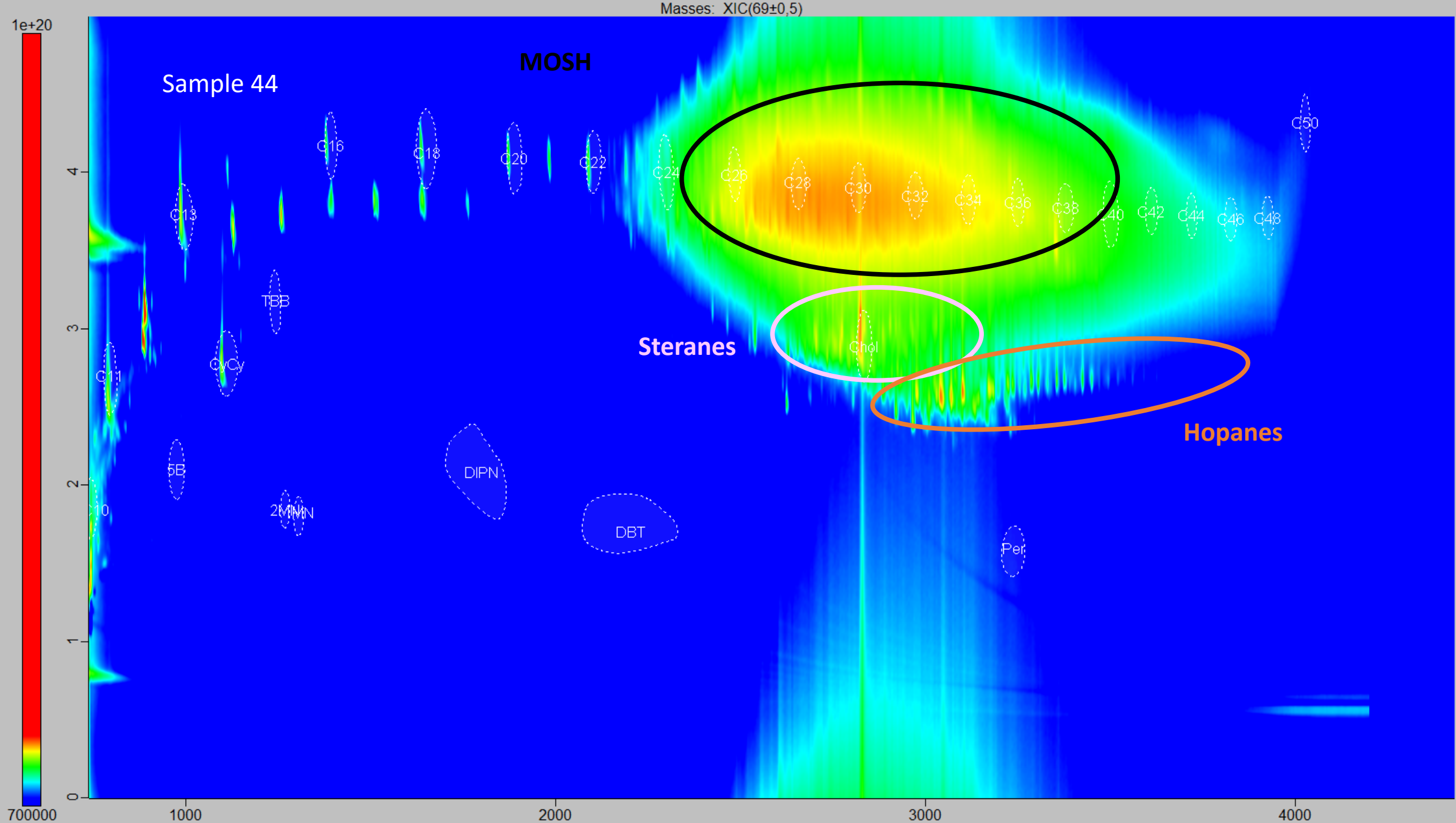

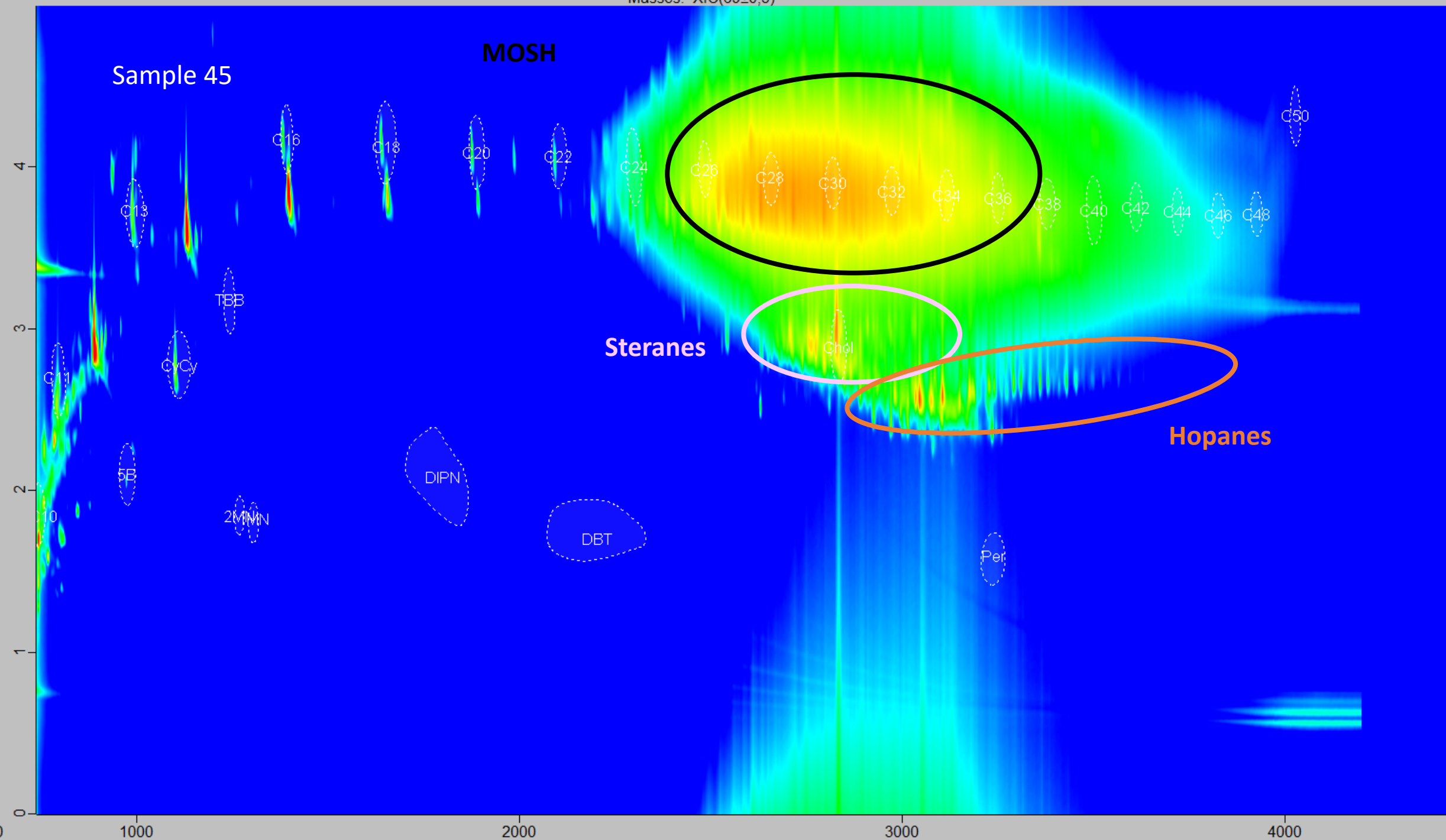

1e+20

700000

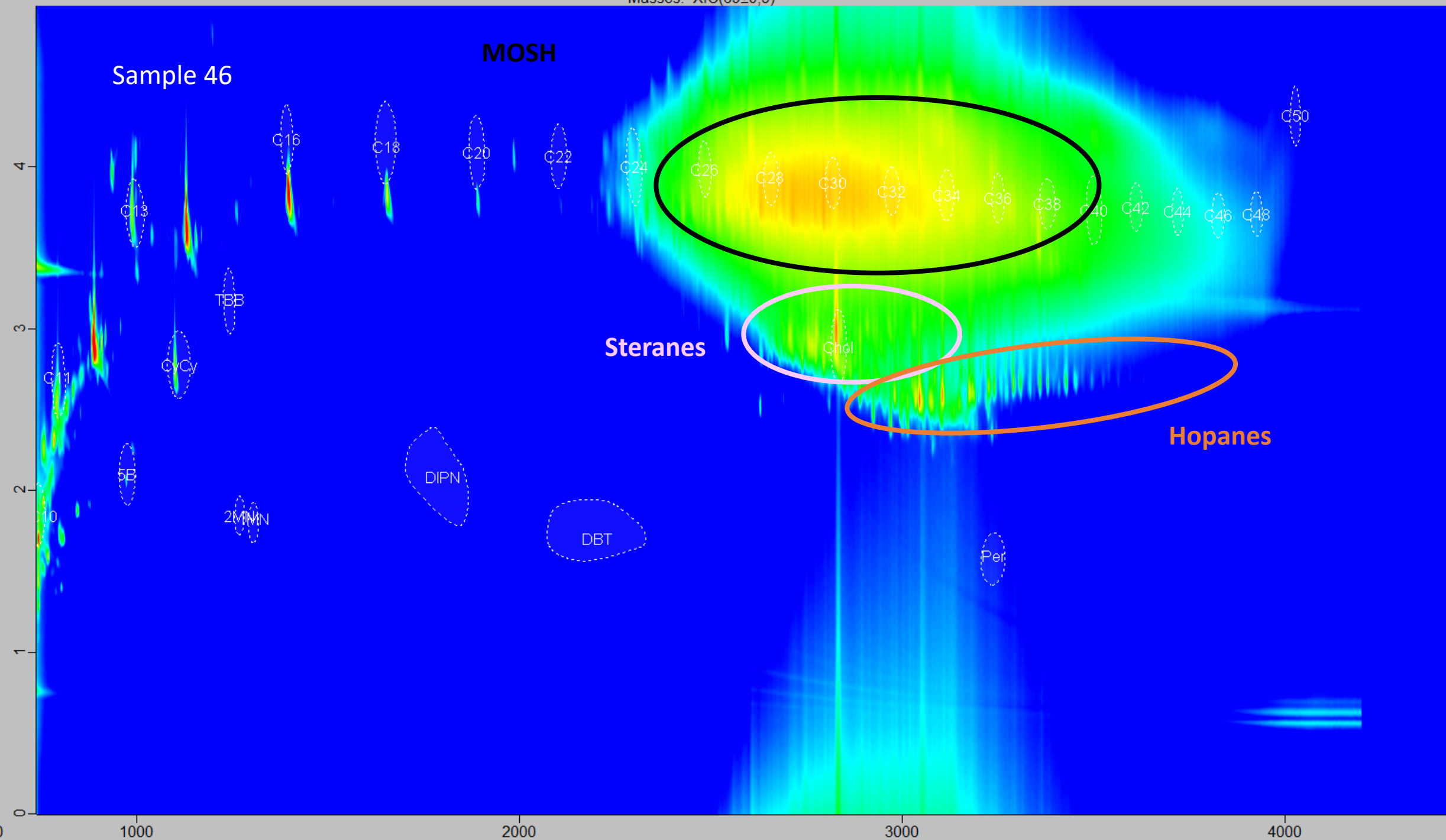

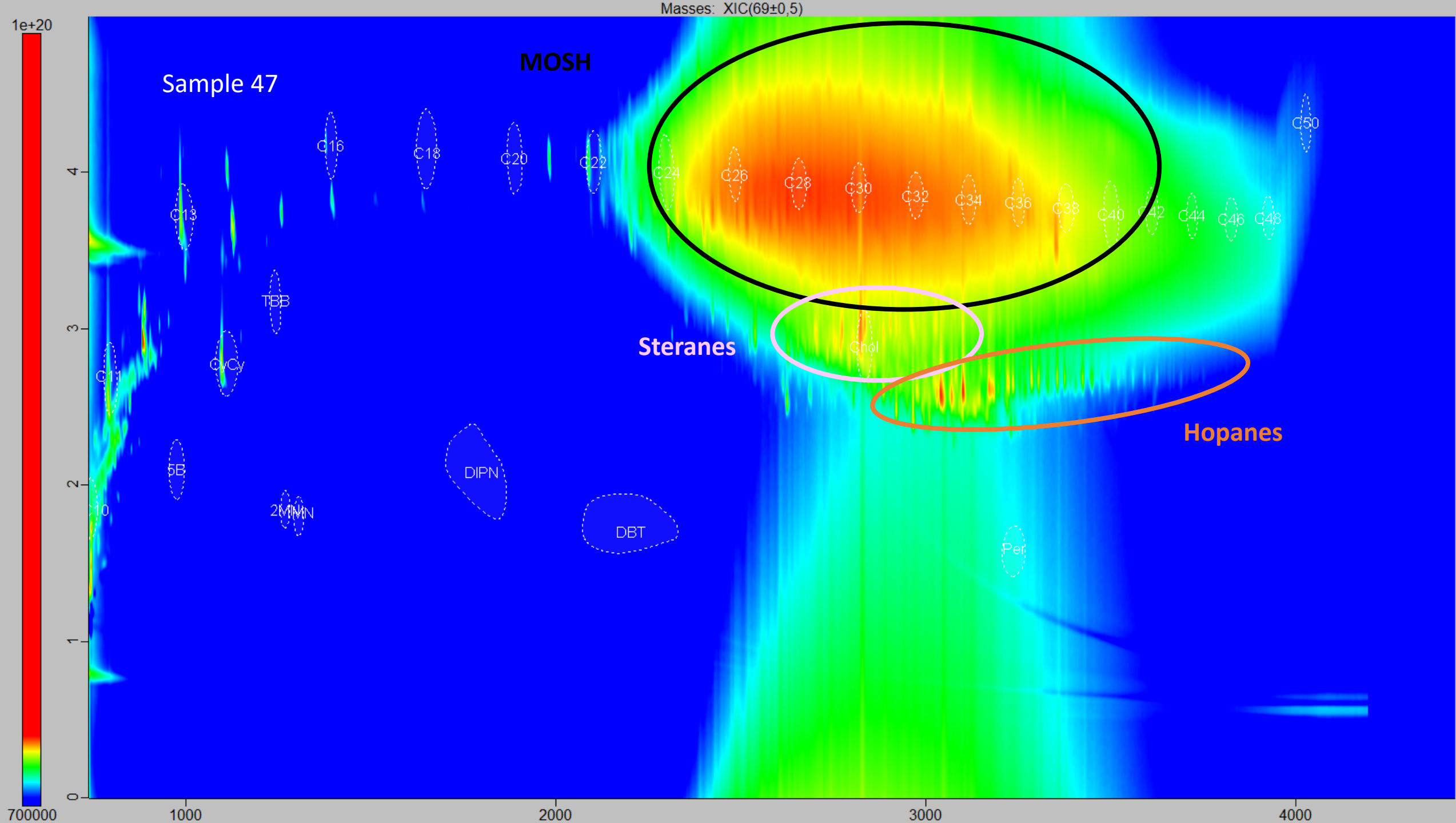

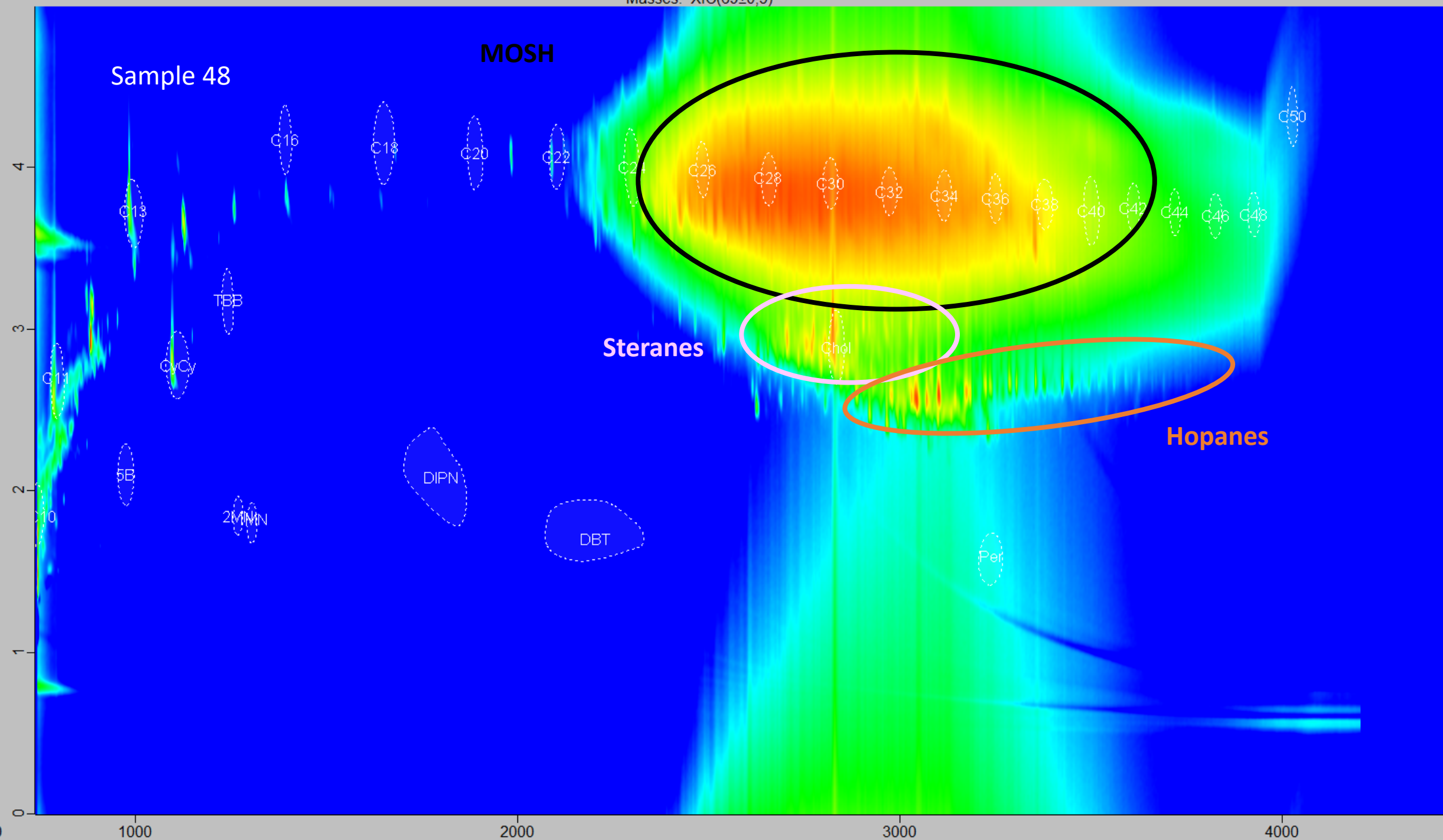

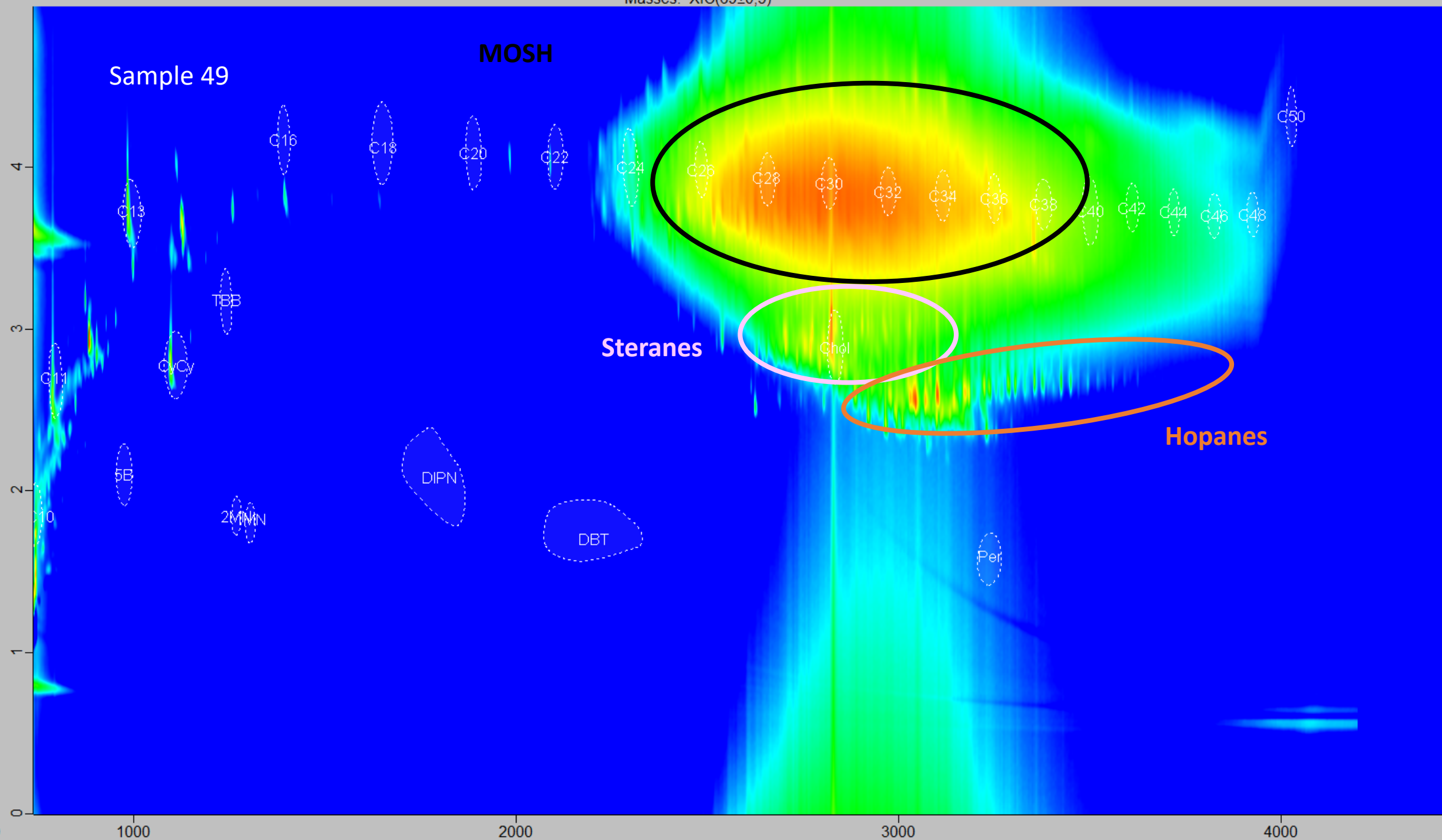

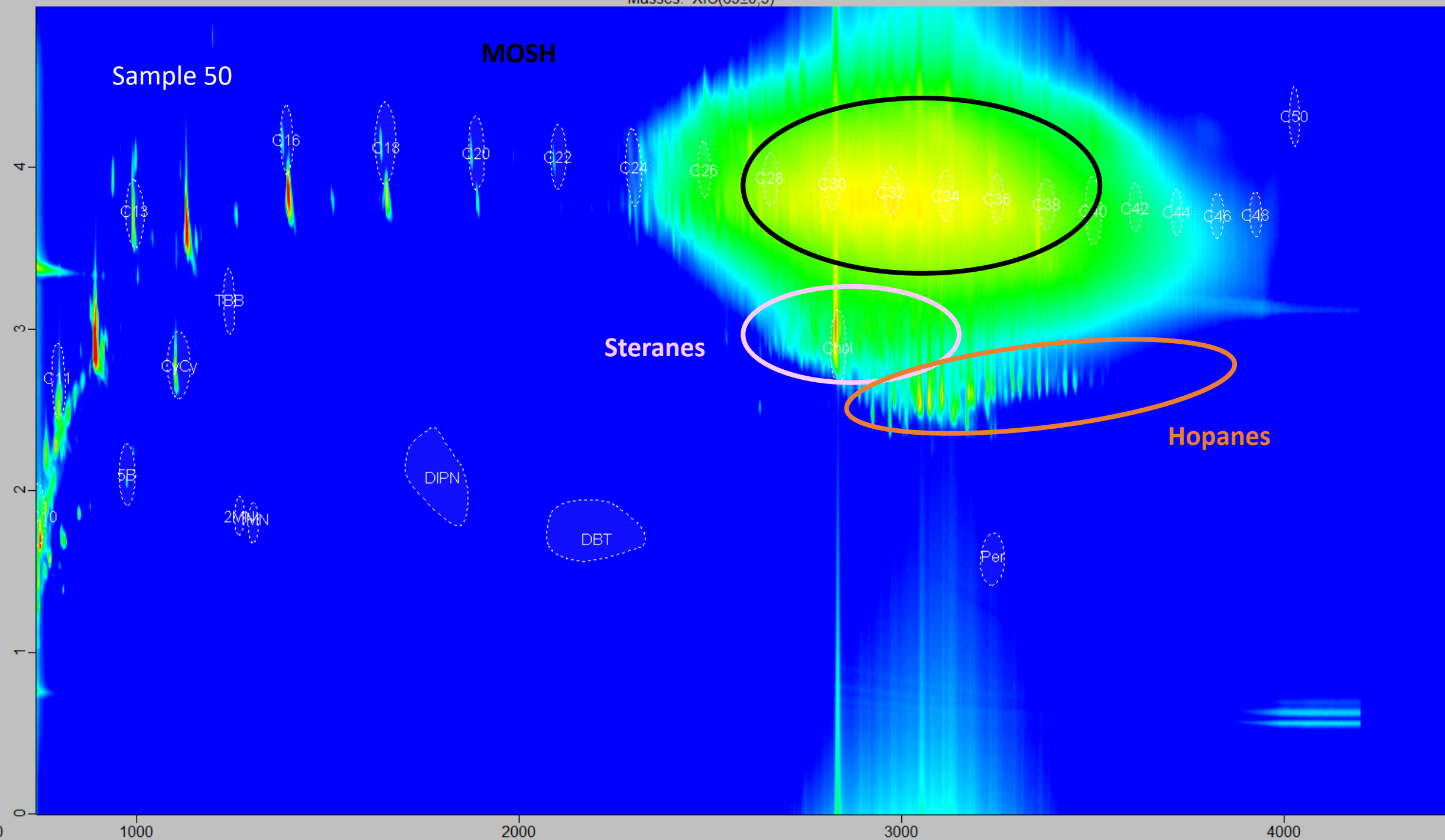

1e+20

700000

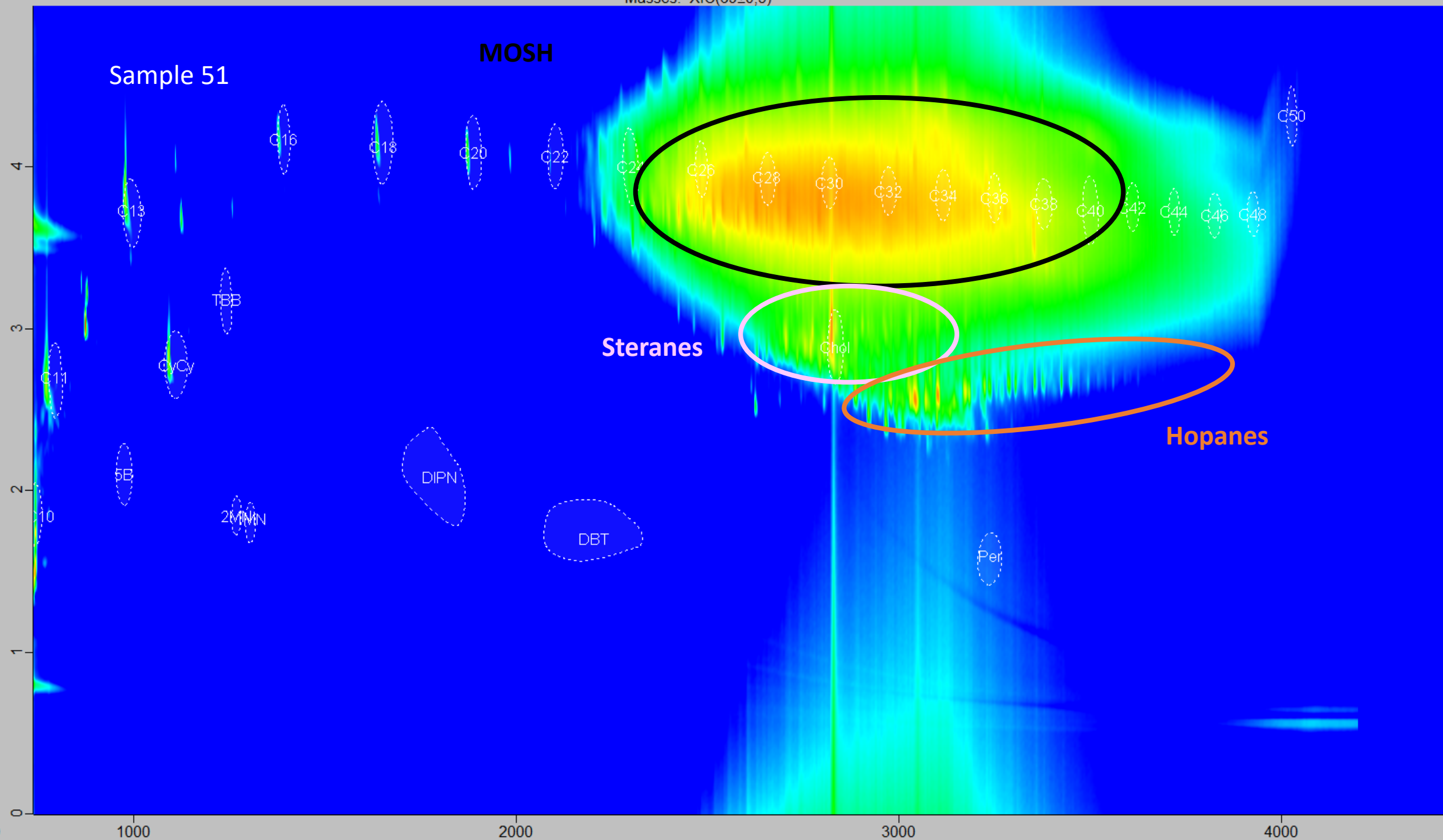

Supplement: Supplementary file 1 [file foods-12-00434-s001.zip › Figure S1.pdf]
